# The Fragmented Mitochondrial Ribosomal RNAs of *Plasmodium falciparum*

**DOI:** 10.1371/journal.pone.0038320

**Published:** 2012-06-22

**Authors:** Jean E. Feagin, Maria Isabel Harrell, Jung C. Lee, Kevin J. Coe, Bryan H. Sands, Jamie J. Cannone, Germaine Tami, Murray N. Schnare, Robin R. Gutell

**Affiliations:** 1 Seattle Biomedical Research Institute, Seattle, Washington, United States of America; 2 The Institute for Cellular and Molecular Biology, and the Center for Computational Biology and Bioinformatics, The University of Texas at Austin, Austin, Texas, United States of America; 3 Department of Biochemistry and Molecular Biology, Dalhousie University, Halifax, Nova Scotia, Canada; Newcastle University, United Kingdom

## Abstract

**Background:**

The mitochondrial genome in the human malaria parasite *Plasmodium falciparum* is most unusual. Over half the genome is composed of the genes for three classic mitochondrial proteins: cytochrome oxidase subunits I and III and apocytochrome *b*. The remainder encodes numerous small RNAs, ranging in size from 23 to 190 nt. Previous analysis revealed that some of these transcripts have significant sequence identity with highly conserved regions of large and small subunit rRNAs, and can form the expected secondary structures. However, these rRNA fragments are not encoded in linear order; instead, they are intermixed with one another and the protein coding genes, and are coded on both strands of the genome. This unorthodox arrangement hindered the identification of transcripts corresponding to other regions of rRNA that are highly conserved and/or are known to participate directly in protein synthesis.

**Principal Findings:**

The identification of 14 additional small mitochondrial transcripts from *P. falcipaurm* and the assignment of 27 small RNAs (12 SSU RNAs totaling 804 nt, 15 LSU RNAs totaling 1233 nt) to specific regions of rRNA are supported by multiple lines of evidence. The regions now represented are highly similar to those of the small but contiguous mitochondrial rRNAs of *Caenorhabditis elegans*. The *P. falciparum* rRNA fragments cluster on the interfaces of the two ribosomal subunits in the three-dimensional structure of the ribosome.

**Significance:**

All of the rRNA fragments are now presumed to have been identified with experimental methods, and nearly all of these have been mapped onto the SSU and LSU rRNAs. Conversely, all regions of the rRNAs that are known to be directly associated with protein synthesis have been identified in the *P. falciparum* mitochondrial genome and RNA transcripts. The fragmentation of the rRNA in the *P. falciparum* mitochondrion is the most extreme example of any rRNA fragmentation discovered.

## Introduction

The human malaria parasite *Plasmodium falciparum* has a most unusual mitochondrial (mt) genome. It consists of tandem repeats of a 5967 nt sequence which encodes open reading frames similar to the genes for cytochrome *c* oxidase subunits I and III (*cox1* and *cox3*) and apocytochrome *b* (*cob*) of other organisms [1]. Mt DNAs also characteristically encode the rRNAs needed for mt translation [2]. While sequences similar to regions of large subunit (LSU) and small subunit (SSU) rRNAs are encoded by the *P. falciparum* mt genome, they are scattered across both strands of the genome, interspersed with each other and the protein coding genes, and correspond to small RNAs [3], [4]. Fragmented rRNAs have been reported from other organisms [5]–[13] and in some instances, most notably for *Chlamydomonas reinhardtii* mitochondria [5], the fragments are encoded out of order with each other. However, the *Chlamydomonas* mt rRNA fragments correspond to the majority of the eubacterial rRNA structure while those of *Plasmodium* species map to a smaller percentage of the bacterial rRNA. The *Caenorhabditis elegans* mt rRNAs [14] and those of kinetoplastid protozoans [15]–[18] are very small but are composed of continuous LSU and SSU rRNAs. The combination of a high degree of fragmentation and the small size of the fragments makes the *P. falciparum* mt rRNAs among the most unusual described.

The features of a particularly small mt genome with just three protein coding genes and highly fragmented mt rRNAs appear to be conserved among the phylum Apicomplexa, to which *P. falciparum* belongs. The mt DNA sequence has been determined for numerous species of *Plasmodium*, which demonstrate strong overall conservation. The sequence and transcription of the mt genome of the rodent parasite *Plasmodium yoelii* have been examined in some depth [19], [20], demonstrating the same structural and genetic organization as for *P. falciparum*. Mt genomes of other *Plasmodium* species, while not as thoroughly analyzed, share these characteristics [21] (also described in this work), as do related species. *Theileria parva* is an apicomplexan cattle parasite having a ∼6.6 kb mt genome [22], [23]. The same three protein coding genes are present as in *P. falciparum* and a few rRNA fragments corresponding to domains IV and V of the LSU rRNA have been assigned by sequence analysis in *T. parva* [23]. The gene order for the *T. parva* mt genome differs from that of *Plasmodium*, however, as does the physical form of the genome. The *Plasmodium* mt genome occurs in tandem repeats but the *T. parva* mt genome consists of single linear copies of the genome bounded by inverted repeat sequences [23]. The mt genomes of additional *Theileria* species and of several *Babesia* species [24] have also been sequenced and are similar in character to that of *T. parva*, consistent with these genera both being in Piroplasmida. Those of *Eimeria tenella*, a coccidian, have much the same genes but show a third variation on mt gene order [25]. The mt genomes of *Hepatocystis*, *Leucocytozoon*, and *Hemoproteus* species, lesser known apicomplexans, closely resemble *Plasmodium* mt genomes in structure and content [26]; they and *Plasmodium* species are all members of Order Haemosporidia. Highly fragmented mt rRNAs thus appear to be a common feature of apicomplexans.

Assignment of fragmented rRNAs based on DNA sequence similarities alone can be misleading. Consequently, evidence that the *P. falciparum* mt rRNA-like sequences are transcribed is an important factor in establishing their potential as components of functional mt ribosomes. We have previously reported a total of 20 small transcripts mapping to the *P. falciparum* mt genome [4]. They range from 40 to 195 nt in length and many carry non-coded oligo(A) tails of unknown function [27], [28]. A majority of the small RNAs are similar to regions of rRNA sequences from other organisms [3], [4], although they are encoded in the mt genome out of linear order with respect to conventional rRNAs. Taken together, they comprise many of the highly conserved sequences in rRNAs. However, RNAs corresponding to many regions of the rRNAs were not previously detected, including the GTPase center in domain II of the LSU rRNA. The two candidate sequences initially thought to comprise that region were each predicted to be ∼100 nt long [3] but evidence for transcripts in that size range was not found [4]. With the recent proliferation of large-scale sequencing projects, small transcripts complementary to mt DNA have been reported from *P. falciparum* cDNA libraries, and the similarity of some of these to rRNA regions has been noted [29].

Detailed crystallographic data for ribosomes and ribosomal subunits have become available in recent years, greatly assisting our understanding of the functional correlates of rRNA and protein structures. In 2000, Ban *et al*. [30], Wimberly *et al*. [31], and Schuenzen *et al*. [32] published high-resolution three-dimensional crystal structures of the *Haloarcula marismortui* large subunit and the *Thermus thermophilus* small subunit of the ribosome, respectively. A year later, Yusupov *et al.* [33] published the structure of the assembled *T. thermophilus* 70S ribosome. More detailed structural analyses have followed, including lower resolution analyses of the mammalian [34], [35] and *C. elegans* [36] mt ribosomes and mammalian cytoplasmic ribosomes [37]. More recently, higher resolution three-dimensional structures of two eucaryotic cytoplasmic small ribosomal subunits were published, from *Saccharomyces cerevisiae* [38] and *Tetrahymena thermophila* [39]. The mechanism of action of antibiotics has been structurally probed [40]–[42], the mechanism of peptide bond formation has been evaluated [43]–[49], specific interactions between rRNA and protein components of ribosomes have been assessed [50]–[53], and the structure of the polypeptide exit tunnel has been revealed [54]. This wealth of structural data, augmented with comparative information that reveals patterns of sequence and structure conservation [55], provides a framework for further evaluation of the unusual *Plasmodium* rRNAs.

**Figure 1:**
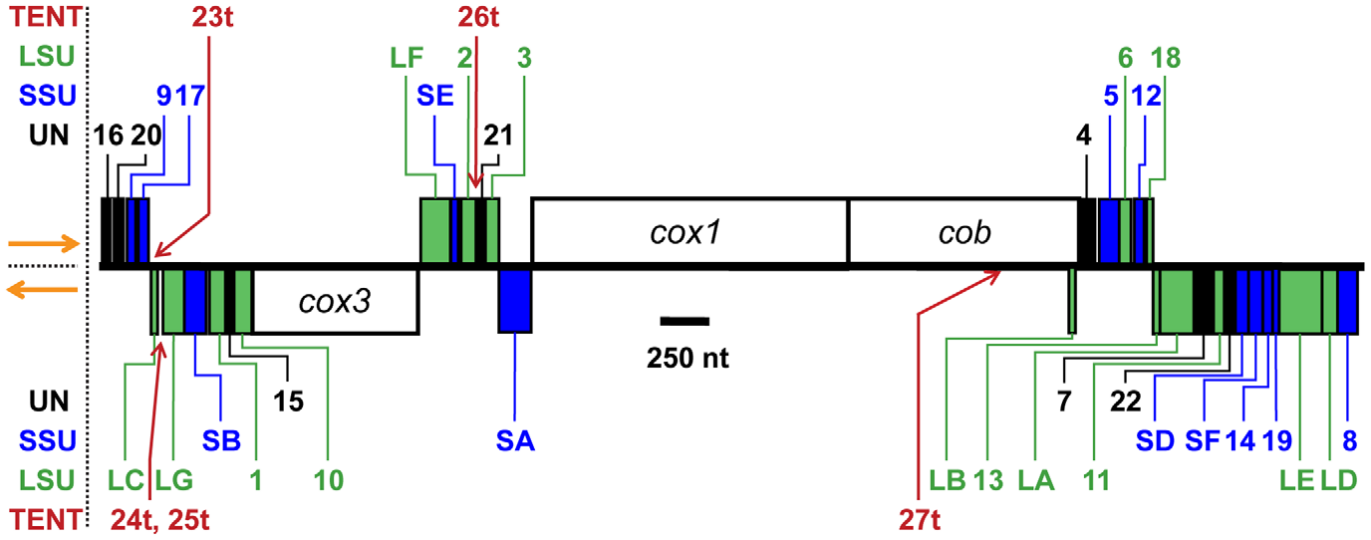
Schematic map of the *P. falciparum* mt genome. A schematic map of the 6 kb element is shown, with genes above or below the line depending on direction of transcription (orange arrows: left to right above the line and right to left below). Because the *P. falciparum* mt genome is tandemly repeated, the endpoints shown are the endpoints of Genbank submission M76611, rather than actual structure. Protein coding genes are indicated by white boxes, small mt rDNA sequences are shown with blue (SSU rRNA) and green (LSU rRNA) boxes and labels, and the locations of RNA23t-RNA27t are indicated with red arrows and labels. Gene abbreviations: *cox*1 and *cox*3, cytochrome *c* oxidase subunits I and III; *cob*, cytochrome *b*; LA-LG, LSU rRNA fragments; SA-SF, SSU rRNA fragments; 1–22, RNA1-RNA22; 23t-27t, RNA23t-RNA27t.

We here report mapping an additional 14 small transcripts to the *P. falciparum* mt genome, yielding a total of 34 such transcripts (Figure 1). Using multiple analyses, we have identified 27 of them as rRNA fragments assigned to specific regions of SSU and LSU rRNA. The regions represented are highly similar to the small but contiguous mt rRNAs of *C. elegans*, and are consistent with a functional role in mt protein synthesis for these highly fragmented rRNAs.

## Results

### Plasmodium mt DNA Conservation

Complete (or nearly complete) mt genome sequences are currently available for 25 species and one *Plasmodium* isolate collected from a mandrill, and for eight species in four related genera of hemosporidians (Table S1). All of them have the same order and orientation of the protein coding genes and the remaining sequence is highly conserved. We aligned representative sequences from each *Plasmodium* species, the *Plasmodium* isolate, and the related hemosporidians, later adding mt DNA sequences from another 184 *Plasmodium* mt DNAs (Table S1). Based on the alignments, the closest relatives of *P. falciparum* are parasites of chimpanzees: *P. reichenowi*, *P. billcollinsi*, and *P. billbrayi*, the latter two being recently suggested species [56]. (Gorillas harbor a parasite that appears even more closely related to *P. falciparum* [57] but a full mt DNA sequence is not available.) The mt DNA sequences of the three chimpanzee parasites are 97.4%, 94.2%, and 91.7% identical to that of *P. falciparum* (M76611), respectively. The mt DNAs of other primate malarias, including the human parasites *P. vivax, P. ovale, P. malariae, P. knowlesi,* and multiple species that parasitize monkeys, are 87.3%–89.1% identical to *P. falciparum*. Those of rodent, lizard, and avian malaria parasites are 87.8%–88.3% identical to *P. falciparum*. The conservation of the non-*Plasmodium* hemosporidian mt DNAs relative to that of *P. falciparum* is 82.6%–86.8%. Some of the variation reflects minor size differences among the mt genomes (Table S1). Much reflects silent or conservative substitutions in the three protein coding genes.

Variations between individual sequences may represent polymorphisms or sequencing errors instead of real differences. Mt genomes have been sequenced from numerous geographically distinct *P. falciparum* [58], [59] and *P. vivax* lines [60]. There are comparatively few polymorphic sites within these species, principally silent substitutions in the protein coding genes. There is far less redundancy of mt DNA data from other *Plasmodium* species; for some species, only a single sequence is available. However, some of those less-represented species are very closely related; for example, the mt DNA sequences of *P. cynomolgi*, *P. fieldi*, and *P. simiovale* are ∼99% identical to that of *P. vivax* so shared variation is likely real. Some of the sequence variation reflects single nt differences in just one of the species. Prudence is warranted when interpreting these as they may be idiosyncratic to a specific isolate or may reflect sequencing errors. Many of the mt DNAs were generated by PCR amplification, adding another possible source for variation. Additionally, the PCR-generated mt genomes typically lack a 50–100 nt region. Because the *P. falciparum* mt genome is tandemly repeated, PCR amplification with neighboring divergent primers yields a nearly complete genome, missing only the sequence between the primers. Sequences generated from different isolates or species in the same study lack the same region so these presumably reflect primer location. Such mt DNA sequences were included in our analyses, with the missing sequence ignored. We did not include other partial or incomplete mt genome sequences in our analyses.

The size of the complete *Plasmodium* mt genomes varies from 5864 nt (*P. billcollinsi and P. billbrayi*) to 6014 nt (*P. juxtanucleare*). The differences relate primarily to nt additions/deletions at the junctions between genes–both mRNAs and small RNAs–or in the 281 nt of apparently non-coding sequence. This is so marked that gaps in sequence alignments almost always correspond to the mapped locations of transcript ends (Table 1). Other hemosporidian mt genome lengths range from 5935 nt (*L. sabrasezi*) to 6684 nt (*L. majoris*), except for *Hepatocystis*, which has substantial insertions relative to the other mt genomes (discussed below). For each, most of the variation between sequences is due to differences at the junctions between genes. Many of the alignment gaps occur at an A run, with the number of 3′ A residues (or T for the complementary strand) in mt DNAs often differing between species. Nearly all of the *P. falciparum* small RNAs are oligoadenylated after transcription so differences in the number of encoded A residues at the 3′ end of an RNA may not matter. Addition of A residues post-transcriptionally may thus render the RNAs more similar among the species than are their genes.

**Table 1:** *P. falciparum* small mt RNA mapping and identification.

gene^a^	*P. falciparum* M76611^b^ coordinates	*T. parva* Z23263 coordinates	Raabe, *et al.* ^29^cDNAs^c^	size RNA/DNA(*P. falciparum*)^d^	subunit/order^e^
SSUA	2023-1916	3666-3561	mtR_6043/27	125/108	SSU/4
SSUB	505-390	4182-4074	none complete	116/116	SSU/6
SSUD	5446-5379	3800-3733	mtR_111/36	65/68	SSU/10
SSUE	1638-1680*	2095-2057	mtR_215/7	40/42	SSU/11
SSUF	5507-5447	1840-1900	mtR_11b/1105	74,58/61	SSU/12
LSUA	5201-5026	4073-3906	none complete	175/176	LSU/1
LSUB	4618*-4586	3667-3691	mtR_143/27	30/29	LSU/3
LSUC	225*-204	1824-1839	mtR_203/2	23/22	LSU/4
LSUD	5854-5772	3114-3196	mtR_5773a/31	85/83	LSU/8
LSUE	5771-5577	3199-3377	none complete	190/195	LSU/9
LSUF	1516-1630	3507-3395	none complete	125,110/115	LSU/11
LSUG	389-283	5667-5775	mtR_36a/221	110/107	LSU/12
RNA1	593-506	3827-3905	mtR_19/19	95/88	LSU/6
RNA2	1698-1763	3098-3032	mtR_69c/15	75/67	LSU/2
RNA3	1830-1910	5658-5584	mtR_5713a/91	85/81	LSU/7
RNA4	4625-4696		mtR_37a/410	75/72	
RNA5	4716-4802		mtR_5822/61	92/87	SSU/9
RNA6	4808-4865	1643-1584	mtR_92/41	58/58	LSU/15
RNA7	5283-5202	1678-1758	mtR_5837/69	94/82	
RNA8	5954-5855	3031-2940	mtR_41/38	115/100	SSU/5
RNA9	72-125	4211-4183	mtR_182b/7	62/54	SSU/8
RNA10	724-625	4359-4301	mtR_140a/29	100/100	LSU/13
RNA11	5340-5284	2300-2240	mtR_14a/12	78/57	LSU/5
RNA12	4887-4945		mtR_52b/35	68/59	SSU/2
RNA13	5025-4996	4401-4373	mtR_5844/63	48/30	LSU/10
RNA14	5546-5508	2039-1997	mtR_11c/62	40/39	SSU/1
RNA15	624-594	1759-1790	mtR_76b/163	40/31	
RNA16	3-33		mtR_60/21	?/31	
RNA17	126-165	2192-2154	mtR_18a/132	?/40	SSU/3
RNA18	4964-4988	1518-1494	mtR_104/23	?/25	LSU/14
RNA19	5576-5547		mtR_42/9	?/30	SSU/7
RNA20	34-71		mtR_86/15	54/38	
RNA21	1807*-1829		mtR_26/26	?/23	
RNA22	5378-5341		mtR_59/124	?/38	
RNA23t	171-203		mtR_21/182		
RNA24t	262-224		mtR_39/36		
RNA25t	283-262		mtR_40b/15		
RNA26t	1764-1806		mtR_49/72		
RNA27t	4138-4085		mtR_84/122		

a Naming convention reflects history of identification [4]. RNA23t through RNA27t are tentative assignments. We have not detected them in RNA blotting experiments, but sequence conservation and comparative abundance of cDNAs [29] suggest they are mature RNAs or abundant processing products.

b 5′ ends are based on primer extension data, except for RNA1 (RNase protection), RNAs 9 and 14–22 (5′ RACE), and RNA21 (Raabe, *et al.* [29] cDNAs and sequence conservation). 3′ ends were determined by 3′ RACE except for SSUE, LSUB, and LSUC (inferred from 5′ end location and transcript size). We have confirming RNase protection data for many of the 5′ and 3′ ends. *, coordinates inferred.

c cDNAs corresponding to mapped *P. falciparum* mt RNAs. Each designation is name/number of cDNAs.

d The observed size from denaturing polyacrylamide gels is noted for the RNA size, compared to the gene size (DNA) based on the location of 5′ and 3′ ends. ?, RNA identified by RACE, not blotting.

e Assignment of *P. falciparum* small mt RNAs to large and small subunit rRNAs; numbers indicate linear order relative to conventional rRNAs.

More substantial size variation occurs at some sites, creating gaps in the alignment (Table S2). The twelve largest gaps include six at the junction of genes transcribed from different strands and six with both genes on the same strand. The largest of the gaps is found between the *cox1* and *cob* ORFs, separated by 20 nt in *P. falciparum* but by 94 in *Hepatocystis*. Transcript mapping has shown that the *P. falciparum cox1* and *cob* transcripts are immediately adjacent [27]. If that is also the case in *Hepatocystis*, the untranslated regions (UTRs) for one or both transcripts would be larger than some of the small mt RNAs. A thirteenth gap is a 51 nt insertion in the *Hepatocystis cob* ORF toward the 3′ end. The insertion maps to the 3′ end of LSUB, and may or may not be included in the *Hepaptocystis* LSUB RNA. If included, it would be a 3′ tail for LSUB, and easily accomodated in the structure. The potential effect on COB is greater. The *Hepatocystis* amino acid sequence is well-conserved relative to that of *Plasmodium* prior to and following the insertion. The DNA sequence of the insertion contains two stop codons but it seems unlikely that it is an intron. These are not common in mt-encoded genes and there is no other evidence for introns in apicomplexan mt genomes. Alternate explanations are that *Hepatocystis* has a truncated but functional COB, or that the stop codons reflect sequence errors. (Since only one *Hepatocystis* sequence is available, the latter explanation seems quite plausible.) The inserted sequence, if translated, would have a hydrophobic character, consistent with the membrane protein COB, and the LSUB RNA would be unaffected, other than a possible tail.

In several cases, some or all of the gap sequence is highly conserved among species. A run of 13 nt in the gap between *cox3* and LSUF is conserved in all the species we examined, as are seven of the eight nt immediately adjacent to LSUF. Of 21 nt between RNA6 and RNA12, 15 are conserved in all the species we examined. Even when there is not broad sequence conservation, there tends to be conservation among closely related species, consistent with related hosts (Table S1). However, species with the same size of intergenic region (Table S2) do not necessarily have the same sequence.

A handful of sites have 4–7 nt discontinuities in the alignment; these are invariably located at the 3′ or 5′ end of a gene. Differences in nt sequence also tend to cluster near the ends of the small RNAs and in the intergenic regions. RNA10, which is assigned to the sarcin ricin loop (SRL) is the principal exception (Figure S1). The 3′ part of RNA10 contains the SRL as part of a 43 nt sequence block that is nearly identical among *Plasmodium* species. A 13 nt block at the 5′ end of RNA10 is identical among species. Between these sites, there is considerable variation, including additions/deletions. Overall, the mt DNAs of *Plasmodium* and related hemosporidians are very highly conserved.

### Identification of Additional P. Falciparum Small mt RNAs

The GTPase center of the ribosome plays a crucial role in translocation so assignment of corresponding RNA fragments is a necessity to postulate a functional mt ribosome. Our attempts to locate such fragments by northern blot analysis were initially based on the expectation from sequence analysis that these RNAs would be ∼100 nt long; these attempts were unsuccessful [4]. One alternate possibility was that other small RNAs from the mt DNA might encode the GTPase functions. However, none of the five RNAs that were unassigned to specific regions of rRNA at that time appeared similar to the GTPase center sequences [4]. Completion of the mt genome sequence from several other *Plasmodium* species provided another means to look for small RNAs. When we aligned the mt DNAs of *Plasmodium* species, we found 100% conservation in two short regions which were similar to the GTPase center: a region 26 nt long for helix H1057 and 16 nt long for helix H1087 (Figure S2A). We therefore re-examined transcription for these very small, highly conserved regions using oligonucleotide probes complementary to the conserved sequences. The result was detection in *P. falciparum* RNA of a ∼30 nt RNA (LSUB) and a ∼23 nt RNA (LSUC) for the 26 and 16 nt conserved regions, respectively (Figure S2B). We believe that, despite their very small size, these RNAs provide the GTPase center of the *Plasmodium* mt ribosome.

The difficulties we encountered in detecting the GTPase center RNAs suggested that other small RNAs might have been missed in prior studies. The nucleotide sequences of regions known to encode putative rRNA fragments are typically conserved among *Plasmodium* species at 90% or greater (Table S3). In comparison, while the amino acid sequences of the three protein genes are conserved at >95% among these species, the nucleotide sequence for the mRNAs is only ∼85% conserved (Table S3). These data provided a criterion for re-examination of *P. falciparum* mt transcripts. We analyzed all apparently non-coding mt sequences >15 nt long that were conserved at ∼90% or better among *Plasmodium* species. By RNA blotting and hybridization (Table S4), we found additional transcripts: RNA11 (assigned to LSU rRNA), RNA12 and RNA14 (both assigned to SSU rRNA), RNA15, and RNA16 (Figure S3). Comparison of the *P. falciparum* and *T. parva* mt DNAs suggested another possible LSU rRNA fragment, and RNA blotting confirmed the presence of a small RNA, now labeled RNA13 (Figure S3). We also evaluated small regions of very high conservation by 5′ and 3′ RACE (Table S4), confirming six additional RNAs under 40 nt long: RNA17 and RNA19 (both assigned to SSU rRNA), RNA18 (assigned to LSU rRNA), RNA20, RNA21, and RNA22 (Table 1).

After completion of our transcript mapping but prior to completion of our sequence analysis, Raabe *et al.* [29] published an analysis of non-protein coding RNAs identified from size-selected *P. falciparum* cDNA libraries. They reported >100 such RNAs corresponding to the mt genome. We analyzed their data with ours, comparing the location of transcript ends and, for those for which RNA blots were shown, whether the observed transcripts were consistent with the expected size of the small RNAs and their potential precursors. They found cDNAs corresponding to all the small RNAs we identified (Table 1), though their cDNAs for LSUE (190 nt), LSUA (175 nt), LSUF (125 nt), and SSUB (116 nt) are not full-length and cDNAs for some of the others have minor variance in 5′ and 3′ ends. Many of the remaining cDNAs lack 5′ and/or 3′ ends consistent with our mapped transcript ends, some include parts of adjacent small RNAs, and all were represented by just a few copies each (<10). In contrast, cDNAs corresponding to the RNAs we mapped typically were abundant (>10 to 1000 copies), except that some of the smallest RNAs were poorly represented (LSUC, SSUE, and RNA19 each have <10 cDNAs). The abundance of a particular cDNA is not reliably diagnostic of the corresponding RNA abundance. However, correlation of our mapped transcripts to the more abundant cDNAs [29] is suggestive. We believe that the less abundant cDNAs may be products of RNA degradation or breakage during experimental manipulation.

With identification of these new small RNAs, only 4.2% of the *P. falciparum* mt genome–251 of 5967 nt, spread across 15 sites–lacked mature transcripts. The two largest sites are the 38 nt 3′ and 58 nt 5′ flanks of LSUC. These include a 26 nt block and a 14 nt block, respectively, in which the sequence is identical among the *Plasmodium* species and the other hemosporidians. Although we did not find evidence for mature RNAs derived from these sequences, Raabe, *et al.* [29] reported small RNAs from both regions (mtR_21 on the 3′ flank, and mtR_39 and mtR_40b on the 5′ flank). Similarly, they reported a cDNA (mtR_49) corresponding to the 43 nt between RNA2 and RNA21; sequence conservation for this area is low (81.8%) but the variance is largely confined to the 5′ third of the region. A 28 nt region between *cox*3 and LSUF is well-conserved among the *Plasmodium* species (94.1%). The 3′ 15 nt of the region are included in an apparent long-lived precursor RNA for LSUF, as discussed below. The remaining intergenic regions range from 3–21 nt and conservation is typically 50–70% (Table S2). Lower conservation suggests a lack of (conserved) function.

Five of the cDNAs [29] map to well-conserved sequences where we did not find evidence for RNAs; they are represented by >10 cDNAs, and have transcripts of size consistent with the proposed coding region and with processing from precursor transcripts. As noted above, four flank mapped rRNAs and the fifth (mtR_84) is complementary to part of the *cob* gene. Although we did not detect these transcripts, their characteristics are consistent with them being at least long-lived processing intermediates and some may be mature mt RNAs. We have tentatively named them RNA23t through RNA27t (Table 1).

With the possible addition of several RNAs identified by Raabe, *et al.* [29], all of the small RNAs that derive from the *P. falciparum* mt genome have apparently been identified. The rRNA fragments total 2037 nt, the seven unassigned RNAs sum to 328 nt, and three mRNAs cover 3351 nt. Together, they account for 95.3% of the 5967 nt *P. falciparum* mt genome. (Overlapping sequences are only counted once.) However, it is difficult to be absolutely certain. Including the Raabe RNAs would raise coverage to 97.6% of the genome. Additionally, it is clear from the search for GTPase center rRNAs that transcripts can be as short as 23 nt; these are not easily detected. Further, the LSUB fragment overlaps completely the 3′ end of the *cob* gene (but not the previously mentioned *Hepatocystis* insertion) on the opposite strand of DNA (Figure 1). This opens the possibility of alternate strand coding for other small RNAs; Raabe *et al.* [29] report several of these but only one (mtR_84) appears to be plausible. While we have tested all regions of the *P. falciparum* mt genome for transcripts on both strands, not all have been subjected to the more rigorous scrutiny used to detect the GTPase center fragments. These caveats aside, we have mapped 34 small RNAs encoded by the *P. falciparum* mt genome, 27 of which have been assigned to specific regions of rRNA (Table 1).

### Transcript Mapping

A total of three protein coding genes and 20 small RNAs have been previously shown by RNA blotting to derive from the *P. falciparum* mt genome [3], [4], [27], [28], [61]; to these we have now added 14 more small RNAs. The genes on the *P. falciparum* mt genome are closely packed (Figure 1) and both DNA strands are polycistronically transcribed, with individual transcripts presumed to be cleavage products from the precursor [62]. To assist evaluation of the predicted secondary structure and potential interactions of the rRNA fragments, we mapped the 5′ and 3′ ends of the small transcripts. Identifying the 3′ end is especially important for the *P. falciparum* mt RNAs since most have non-encoded oligo(A) tails up to 25 nt long [27] and therefore the RNA size on blots often exceeds the size of the corresponding DNA sequence. We have previously reported the 3′ ends, including oligo(A) tails, of some of the small mt RNAs [4] and here identify the 3′ ends and oligoadenylation status for most of the remaining RNAs (Table S5).

The 3′ ends of the *P. falciparum* small RNAs were identified by 3′ RACE, augmented in some cases by RNase protection. The oligo(A) tails are often too short for priming cDNA synthesis with oligo(dT) so for 3′ RACE, we enzymatically added 3′ C tails to total RNA. This was followed by cDNA synthesis primed with oligo(dG), then PCR amplification with oligo(dG) and gene-specific 5′ primers [28]. The PCR products obtained were cloned and sequenced, revealing the presence of non-encoded As at the 3′ end of most of the small transcripts (Table S5). The length of the A tail is variable but tends to be generally characteristic for each transcript; that is, most cDNAs for specific small RNAs have short A tails while those for others have predominantly long A tails (Table S5). SSUB lacks added 3′ A residues [28] and, with the possible exceptions of SSUE, LSUB, and LSUC (see below), is the only small mt RNA that lacks an oligo(A) tail. The significance of oligo(A) tail presence and size is unknown. Oligoadenylation of mt rRNAs has been previously reported for mammals and insects [63]–[65] but not examined in detail. The phenomenon may simply reflect a promiscuous mt poly(A) polymerase activity.

We obtained 5′ end locations by primer extension, RNase protection, or 5′ RACE; in many cases, more than one mode was used. These data are straightforward with two exceptions. The LSUF rRNA, which corresponds to part of the peptidyltransferase domain of LSU rRNA, has two transcripts detected on RNA blots [4] and two primer extension products (Figure S4); the size difference in each case is 15 nt. The precise relationship between the two transcripts is unknown. The larger transcript could represent an abundant RNA processing intermediate or both sizes of the RNA could be functional, possibly in different roles. (We are unaware of any examples of the latter.) It is notable that the 15 nt 5′ extension of the larger transcript is less conserved among *Plasmodium* species (∼70%) than the remainder of the LSUF RNA (98%), due to considerable variability in the first seven nt (Figure S4). Sequence corresponding to the conserved section of LSUF has been identified in *T. parva* [23], but there sequence corresponding to the 15 nt extension is not conserved. On balance, these data better support the hypothesis that the larger RNA is an abundant precursor. (Probes for SSUF also detect two sizes of transcript on RNA blots [4] but neither primer extension nor 3′ RACE supports an extension such as that seen for LSUF. This may reflect that different oligonucleotides used for the RNA blot, 3′ RACE, and primer extension might have differential ability to hybridize to both RNAs; that hybridization dynamics differ for blot hybridization compared to solution hybridization; or that the RNA blot probe cross-hybridizes to another small RNA.) The second exception involves the 5′ end of RNA21, for which detection of apparent precursor transcripts by 5′ RACE both enlightens and complicates data interpretation, as discussed below.

**Figure 2:**
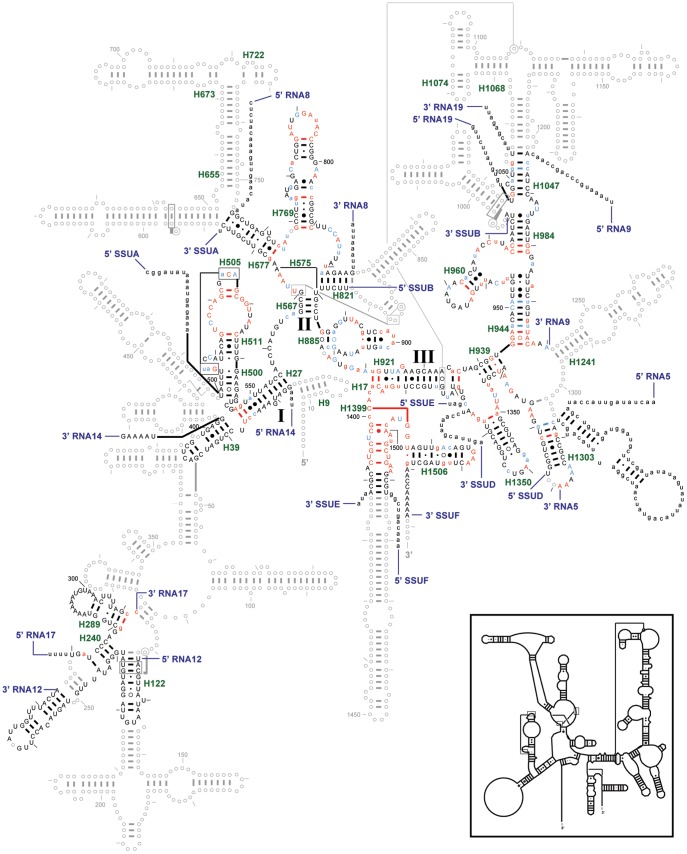
SSU rRNA secondary structure. The *P. falciparum* mt SSU rRNA secondary structure model is superimposed onto the *E. coli* SSU rRNA secondary structure model diagram. Regions where *P. falciparum* has no structure equivalent to *E. coli* are shown using gray circles and lines. Colored nucleotides compare the *P. falciparum* sequence to the Three Phylogenetic Domains/Two Organelles (aka 3P2O) consensus sequence from the Gutell lab’s Comparative RNA Web (CRW) Site [URL for CRW Site: http://www.rna.ccbb.utexas.edu/. URL for 16S rRNA conservation diagram: http://www.rna.ccbb.utexas.edu/SAE/2B/ConsStruc/Diagrams/cons.16.3.3DOM.pdf]. Upper case colored nucleotides are conserved in at least 98% of the sequences used. For the *P. falciparum* mt rRNA fragments: red nucleotides match the 3P2O consensus sequence, light blue nucleotides differ, and black nucleotides cannot be compared to the consensus (which is conserved at less than 90%). Base pair symbols are colored when both of the paired nucleotides have the same color. Each fragment is labeled at its 5′ and 3′ ends. Each helix present in *P. falciparum* is labeled with its helix number in green (*e.g.*, H500), based on the CRW Site’s helix numbering system [URL: 16S rRNA, http://www.rna.ccbb.utexas.edu/CAR/1A/Structures/h.16.b.E.coli.hlxnum.pdf]. **Inset:** *C. elegans* mt SSU rRNA secondary structure model.

Mapping for three of the small RNAs posed technical challenges that we were not able to overcome. The 40 nt SSUE RNA includes a near-universally conserved 16 nt sequence, GUACACACCGCUCGUC. Except for the underlined U, this exactly matches the corresponding sequences from the genome of the *P. falciparum* apicoplast (a relict plastid that encodes its own rRNAs [66]) and the nuclear encoded SSU rRNAs. The small size of the transcript limits options for positioning primers and attempts at 3′ and 5′ RACE for SSUE using total RNA have failed, likely due to cross-hybridization of the primers to these other rRNAs. Use of mt RNA would theoretically alleviate this problem. However, methods for isolation of *P. falciparum* mitochondria [67], [68] provide relatively crude fractionation. We have found RNA from such organelle preparations to be significantly contaminated with non-mt sequences, and were unable to obtain an SSUE 5′ RACE product from the “mt” RNA. Primer extension produced multiple products, some of which also may reflect cross-hybridization to other *P. falciparum* rRNAs. Consequently, the 5′ and 3′ ends of SSUE are predictions, based on the following observations:

- Radiolabeled probes complementary to SSUE detect a 40 nt transcript [4].
- SSUE size does not alter following digestion with RNase H in the presence of oligo(dT), suggesting that the transcript lacks a significant oligo(A) tail [28].
- 5′ RACE for RNA21 produced long products which appear to derive from partially processed precursor transcripts. Two clones have a 5′ end at 1631 and two at 1638. The LSUF 3′ end from 3′ RACE maps to 1630 [28] and the 41 nt conserved region starts at 1638 (Figure S5A).
- SSUE is encoded in a 64 nt region between LSUF and RNA2. A 41 nt portion of that region is 100% conserved among *Plasmodium* species and varies very little in the other hemosporidians. It differs from the other *P. falciparum* SSU rRNAs at just two sites in the 3′ half. The conserved region is flanked by seven slightly variable nt at the 5′ end and the 18 nt 3′ flank is poorly conserved among *Plasmodium* species.
- The 41 nt conserved sequence fits well into the expected secondary structure for this region of SSU rRNA, including maintenance of highly conserved sequence and capacity to form helices with portions of SSUB and SSUF, creating a reasonably conventional secondary structure (Figure 2). However, the final 18 nt of the 64 nt region would not be predicted to participate in a helix with other small mt RNAs.

Taking these points together, it is likely that SSUE is encompassed within the 41 nt highly conserved sequence, with its 5′ end at 1638 and the 3′ end at 1680, and few, if any, As added at its 3′ end.

Mapping LSUB and LSUC presented a different challenge. For RACE, at least one primer must be gene-specific and success is enhanced if nested gene-specific primers can be used. However, the LSUB and LSUC transcripts are only 30 and 23 nt, respectively, so the gene-specific primers are severely limited. That the primers must be small means in turn that temperatures in the initial rounds of PCR must be comparatively low, with accompanying loss of specificity. Despite multiple attempts, we have been unable to obtain RACE products for these RNAs. Given the relative size of the transcripts and their highly conserved sequence, any 3′ oligo(A) residues would likely be too few in number for successful RNase H analysis for A tails. Based on conservation of sequence between the *Plasmodium* species, between *P. falciparum* and *T. parva*, and the size of the transcripts, we have predicted the likely 5′ and 3′ ends as 4618 and 4586 for LSUB and 225 and 204 for LSUC, respectively (Table 1).

### Transcription and Processing

Mt genomes are often polycistronically transcribed, then cleaved to release mature RNAs [69]. We have previously reported evidence for extensive polycistronic transcription of the *P. falciparum* mt genome, including RNase protection data consistent with multigenic precursor transcripts and detection of overlapping, near-genome length RT-PCR products [62]. During RACE analysis of the *P. falciparum* mt small RNAs, we obtained additional evidence in support of polycistronic transcription. As noted above, 5′ RACE with a gene-specific primer for RNA21 generated products that assisted identification of the SSUE 5′ end. Additional products mapped to nt 1754, 1764, 1765, and 1766 of *P. falciparum* mt DNA (Figure S5A). The 3′ end of RNA21 maps to nt 1829 (Table 1) but we were unable to detect RNA21 by blotting so could not use transcript size to assist the analysis. Gaps in the mt DNA alignment, combined with cDNA data [29], suggest there are two transcripts between RNA2 and RNA3. One is RNA21 (mtR-26), a 23 nt transcript with only two variable sites, and the other is RNA26t (mtR_49), a 43 nt RNA that is >90% conserved at all but seven residues toward the 5′ end. RNA2, RNA26t, RNA21, and RNA3 are immediately adjacent to each other in *P. falciparum* but there are 1–2 nt gaps or insertions in many other species, located at the predicted junctions. Ironically, the primer chosen for RNA21 overlapped its 5′ end, but that end was identified from analysis of apparent precursors. The 5′ RACE products mapping to 1764–1766 likely derive from the cleavage that produces the 3′ end of RNA2/5′ end of RNA26t, followed by minor nibbling. (Position 1754 maps within RNA2 and we suspect it reflects an artifact.).

RNA16 and RNA20 abut each other and are transcribed from the same DNA strand (Figure 1). While performing 3′ RACE using a gene-specific primer for RNA16, we obtained two sizes of PCR product. One corresponded to RNA16, including its oligo(A) tail and the other included RNA16 with no A tail followed by RNA20, which had an oligo(A) tail (Figure S5B). Similarly, when performing 5′ RACE with a gene-specific primer for RNA20, we identified not only the 5′ end of RNA20 but also obtained longer products consisting of the expected RNA20 sequences plus all of RNA16 (Figure S5B). These data imply that 3′ cleavage and oligoadenylation of RNA20 can precede the corresponding step for RNA16 and that the 5′ end of RNA16 can form prior to its 3′ end.

The accumulated mapping data have allowed us to identify possible sites of cleavage in the putative precursor transcript. One caveat exists for this analysis; if the 3′ end of an RNA maps to an A residue, it is ambiguous whether that A derives from the DNA sequence or from addition of the oligo(A) tail. Unless there is data suggesting otherwise, we have assumed that 3′ end A residues with corresponding DNA sequence are encoded rather than added by poly(A) polymerase. Based on this criterion, many of the mtRNAs directly abut each other. Indeed, in almost every instance in which genomic As are found at the junction between genes, it appears most likely that they are part of the transcript of the upstream gene. We have identified one exception. At the junction of RNA14 and SSUF (Figure 1), 3′ RACE shows that RNA14 has an oligo(A) tail (Table S5) and there are three A residues at the junction between the two genes. Our default interpretation would assign those As to RNA14. However, 5′ RACE shows that SSUF starts at those A residues. Unless the genes overlap, which is not seen elsewhere in this genome for genes on the same DNA strand (but see below), all of the As at the 3′ end of RNA14 must be added rather than encoded. Similarly, the junction of RNA1 and SSUB lies in a run of four As. 5′ RACE for SSUB showed some transcripts starting with two As and some with three. The number of encoded As at the 3′ end of RNA1 is therefore uncertain, but addition of the A tail renders this moot.

Ribosomal RNAs are typically derived by cleavage from a polycistronic precursor RNA, often via sequential steps. We have previously demonstrated the existence of near-genome length polycistronic transcripts of the *P. falciparum* mt genome [62], suggesting a role for cleavage to produce individual *P. falciparum* mt RNAs. The mapping data indicate that there are four sets of abutted genes, with four to 12 members per set (Table S6). For these, a single cleavage event would suffice to create the 5′ end of one RNA and the 3′ end of its neighbor. Polycistronic transcription of close-packed genes, followed by cleavage to produce individual mRNAs, is a common feature of mt genomes. In vertebrates, most mt genes are flanked by tRNAs, and production of mature mRNAs and rRNAs goes hand-in-hand with tRNA processing [69]. A similar mechanism would be consistent with the accumulated *P. falciparum* data. However, the *P. falciparum* mt genome does not encode tRNAs so another mechanism must guide cleavages. The junctions between transcripts do not share any obvious specific features with each other or with the 5′ or 3′ end sequences immediately adjacent to other *P. falciparum* mt RNAs except for a tendency to have an A as the last encoded residue. Similarly, RNAs which do not directly abut their neighbors also show no conserved characteristics except the tendency to have a 3′ terminal A. Of the 31 small RNAs for which 3′ RACE data is available, the genes for 19 end with A, 2 with C, 3 with G, and 7 with T (Table S5). The composition of the mt genome is 32.39% A, 15.69% C, 15.90% G, and 36.01% T. The prevalence of A residues at the 3′ end of transcripts therefore strongly exceeds that expected by chance. (This point, of course, depends on our interpretation of which A residues are encoded and which are added.) Overall, the mechanism for recognition of putative cleavage sites remains unknown.

### Assignment of New rRNA Fragments

Prior analyses proposed that 15 of the small *P. falciparum* mt RNAs corresponded to specific regions of LSU or SSU rRNAs [4]. These assignments were based on conservation of sequence and/or the potential to form an appropriate secondary structure while maintaining some conserved sites. Assigning fragments which might correspond to less conserved regions of rRNA structure is more challenging, but can be assisted by cross-species comparisons between related organisms. The gene content and organization is identical in all *Plasmodium* species for which complete mt DNAs are available. Indeed, the overall nt conservation is so high (Table S1; Table S3) that comparisons between these are not particularly useful in assigning the small RNAs to positions in the SSU and LSU rRNAs. However, the mt genome of *T. parva* was useful for comparison.

**Figure 3:**
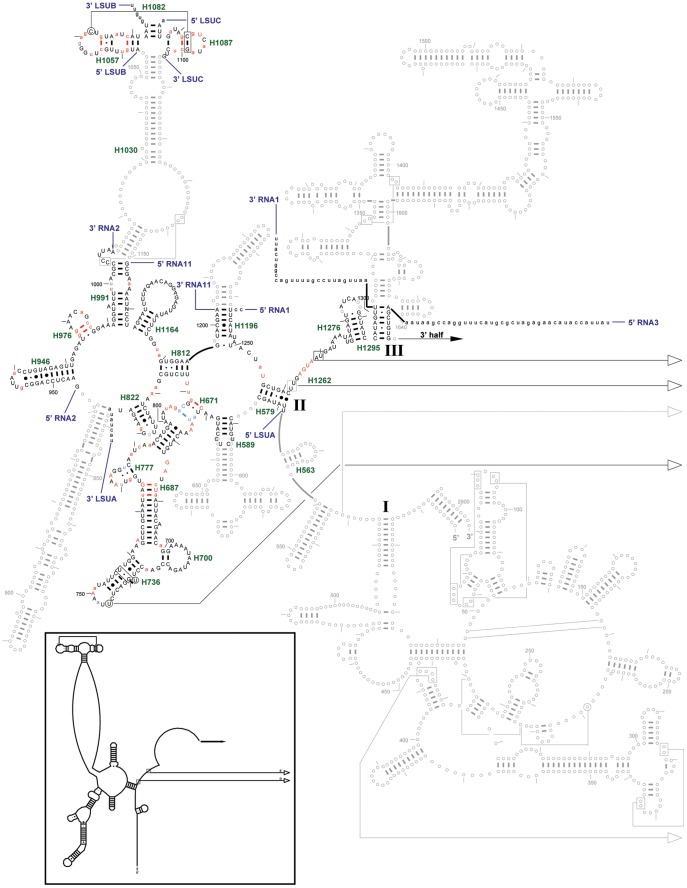
LSU rRNA secondary structure (5′ half.) The *P. falciparum* mt LSU rRNA (5′ half) secondary structure model is superimposed onto the *E. coli* LSU rRNA (5′ half) secondary structure model diagram. Regions where *P. falciparum* has no structure equivalent to *E. coli* are shown using gray circles and lines. Colored nucleotides compare the *P. falciparum* sequence to the Three Phylogenetic Domains/Two Organelles (aka 3P2O) consensus sequence from the Gutell lab’s Comparative RNA Web (CRW) Site [URL for CRW Site: http://www.rna.ccbb.utexas.edu/. URL for 23S rRNA (5′ half) conservation diagram: http://www.rna.ccbb.utexas.edu/SAE/2B/ConsStruc/Diagrams/cons.23.3.3DOM.5.pdf]. Upper case colored nucleotides are conserved in at least 98% of the sequences used. For the *P. falciparum* mt rRNA fragments: red nucleotides match the 3P2O consensus sequence, light blue nucleotides differ, and black nucleotides cannot be compared to the consensus (which is conserved at less than 90%). Base pair symbols are colored when both of the paired nucleotides have the same color. Each fragment is labeled at its 5′ and 3′ ends. Each helix present in *P. falciparum* is labeled with its helix number in green (*e.g.*, H500), based on the CRW Site’s helix numbering system [URL: 23S rRNA 5′ half, http://www.rna.ccbb.utexas.edu/CAR/1A/Structures/h.235.b.E.coli.hlxnum.pdf]. **Inset**: *C. elegans* mt LSU rRNA (5′ half) secondary structure model.

**Figure 4:**
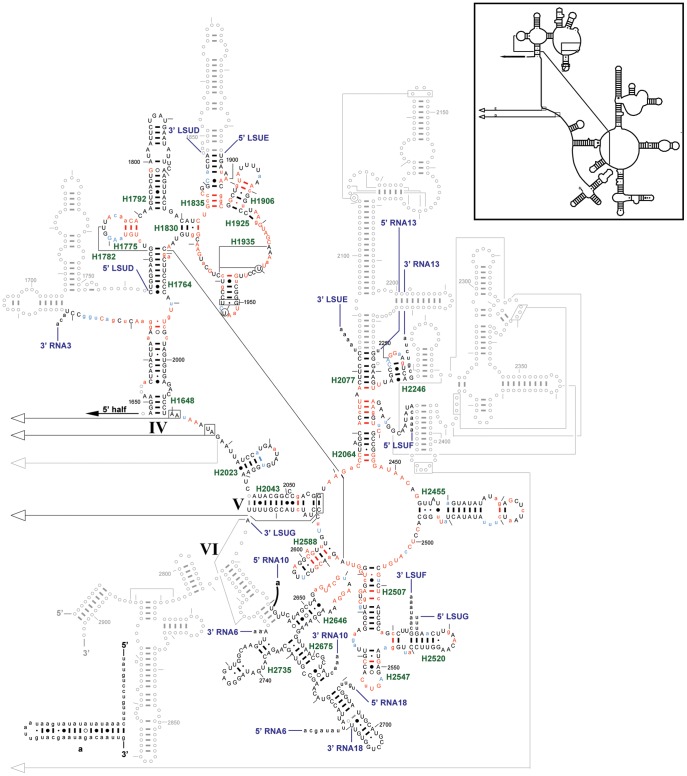
LSU rRNA secondary structure (3′ half.) The *P. falciparum* mt LSU rRNA (3′ half) secondary structure model is superimposed onto the *E. coli* LSU rRNA (3′ half) secondary structure model diagram. Regions where *P. falciparum* has no structure equivalent to *E. coli* are shown using gray circles and lines. Colored nucleotides compare the *P. falciparum* sequence to the Three Phylogenetic Domains/Two Organelles (aka 3P2O) consensus sequence from the Gutell lab’s Comparative RNA Web (CRW) Site [URL for CRW Site: http://www.rna.ccbb.utexas.edu/. URL for 23S rRNA (3′ half) conservation diagram: http://www.rna.ccbb.utexas.edu/SAE/2B/ConsStruc/Diagrams/cons.23.3.3DOM.3.pdf]. Upper case colored nucleotides are conserved in at least 98% of the sequences used. For the *P. falciparum* mt rRNA fragments: red nucleotides match the 3P2O consensus sequence, light blue nucleotides differ, and black nucleotides cannot be compared to the consensus (which is conserved at less than 90%). Base pair symbols are colored when both of the paired nucleotides have the same color. Each fragment is labeled at its 5′ and 3′ ends. Each helix present in *P. falciparum* is labeled with its helix number in green (*e.g.*, H500), based on the CRW Site’s helix numbering system [URL: 23S rRNA 3′ half, http://www.rna.ccbb.utexas.edu/CAR/1A/Structures/h.233.b.E.coli.hlxnum.pdf]. **Inset**: *C. elegans* mt LSU rRNA (3′ half) secondary structure model.

The *T. parva* mt genome is, at ∼6.6 kb, quite small and encodes the same three protein coding genes as the *Plasmodium* mt genomes, but they are aranged in a different order on a linear molecule, flanked with inverted repeats [23], [70]. The genome also encodes small RNAs which have similarity to rRNA sequences, some of which have been previously described [23]. Thus, although the genome organization is quite different from that of *Plasmodium* species, gene content is very similar. We undertook a detailed examination of the *T. parva* mt genome for sequences corresponding to the small RNAs mapped to the *P. falciparum* mt genome. *T. parva* matches were found for 24 of the 27 *P. falciparum* small mt rRNAs and for 2 of the unassigned RNAs (Table 1). Four of the five previously predicted *T. parva* LSU rRNAs [23] match well with our *P. falciparum* mapping data and predictions. The fifth, *T. parva* LSU5 RNA, has only a partial overlap with its *P. falciparum* counterpart, RNA3, as discussed below. With data from two genera, it was possible to identify compensatory changes in sequence which assisted assignment of additional *P. falciparum* RNAs to specific regions of rRNA. The *T. parva* analysis also assisted the identification of corresponding sequences in other apicomplexans (Table S7). The predicted secondary structures for fragments comprising the *P. falciparum* SSU and LSU rRNAs are shown in Figures 2, 3, 4; the corresponding predicted secondary structures for *T. parva* are shown in Figures S6, S7, S8. Conservation at each position is assessed by comparison to all available rRNA sequences from three phylogenetic domains (Archaea, Bacteria, nuclear-encoded Eucarya) and two organelles (mitochondria and chloroplasts), called 3P2O (three phylogenetic domains, 2 organelles).

The *P. falciparum* and *T. parva* mt rRNA secondary structure models are very similar to one another and to the central core of the *Escherichia coli* rRNAs with one notable exception. *E. coli* 23S rRNA positions 1906–1934 are contained within one of the most highly conserved regions of LSU rRNA [55] (http://www.rna.ccbb.utexas.edu/) and, as expected, this region (near the 5' end of LSUE) is conserved in the *P. falciparum* sequence/structure. Surprisingly, the same region of the *T. parva* homologue is much shorter, showing poor conservation [23]. This cannot be explained as a sequencing error because the corresponding mt DNA sequences from *Babesia* and *Theileria* species (Table S1) are also truncated in this region.

Additional evidence to support the placement of RNA fragments to the SSU and LSU rRNAs comes from analysis of covariation, evaluating whether the two positions that form a base pair have similar patterns of variation to maintain this base pair. [71] Three categories of covariation are used to gauge the amount of support for the placement of the RNA fragments. Category 1 base pairs contain only pure covariations, *i.e.*, the patterns of variation in the two columns in the alignment are identical; all of the base pair types (e.g., A:U, G:C, U:A, C:G) covary with the other base pair types. Category 2 base pairs have covariation between the two paired nucleotides, but they also have a low frequency of the two paired positions with different patterns of variation (*e.g.*, base pair types A:U, G:C, with G:U less frequent, or A:U, G:C, with A:A less frequent). Category 3 contains those positions that are either invariant or the patterns of variation at the two paired positions are not the same (*e.g.*, G:C <-> G:U). We analyzed the base pair types present for the mt rRNA fragments from 218 apicomplexans (species of *Plasmodium* and other hemosporidians, the coccidian *Eimeria*, and the piroplasms *Babesia* and *Theileria*). In total, 27 of the 34(39) transcripts for hemosporidians, 20 predicted RNAs for the coccidian, and 24 predicted RNAs for the piroplasms were assigned to rRNA. The fragments that were compared are indicated in Table S7; the category 1 and 2 base pair types are given in Table S8 and shown in Figures S9, S10, S11).

RNA14 is the best candidate among the small RNAs for the 5′ end of the SSU rRNA, being the only fragment which can form an appropriate helix (H27) with SSUA (Figure 2). The *T. parva* sequence predicted to correspond to RNA14 has little sequence similarity to it but has capacity to pair with the *T. parva* SSUA-equivalent and can form a similar predicted structure. The base-pairing potential for the *T. parva* sequence is better than that proposed for *P. falciparum*, thus providing more confidence in our assignment of RNA14 as the 5' end of SSU rRNA. Of the 17 base pairs in the RNA14 fragment, three are category 1 and four are category 2 (Figure S9; Table S8). RNA8 was previously proposed to correspond to the 5′ terminal domain of SSU rRNA (prior to the identification of the RNA14 transcript) [4]; we now show that RNA8 contains the highly conserved 790 loop and interacts with both the 3′ end of SSUA and the 5′ end of SSUB (Figure 2). It has five category 2 base pairs.

Assignment of RNA17 to SSU rRNA was based upon the facts that RNA17 and the *T. parva* homologue both contain a sequence that is very similar to *E. coli* SSU rRNA positions 282–294 and that they contain H289 (Figure 2). RNA12 has the potential to interact with RNA17, providing the remainder of H240 and a truncated H122 (Figure 2). We have paired the central region of RNA19 with RNA9 to form the lower half of H1047. RNA17 has one category 2 covariation; RNA12 and RNA19 have no covariation. The 3′ portion of RNA5 has the potential to basepair with SSUD and together they may comprise H1303. The 5′ portion of RNA5 has the capacity to form an extended helix and large loop; sequences in the proposed helical regions are well-conserved among *Plasmodium* species but the 22 nt loop region varies in both size and sequence (Table S3). Note that since we have not yet found *T. parva* homologues for RNA5, RNA12, or RNA19, assignment of these transcripts to SSU rRNA should be considered tentative.

RNA2 and RNA11 correspond to portions of the 5′ half of LSU rRNA (Figure 3). RNA2 approximately spans *E. coli* LSU positions 944 through 1010, encompassing helices H946, H976 and the 5′ strand of H991. RNA11 pairs with RNA2, providing the 3′ strand of H991. It also forms helix H1164 and pairs with LSUA in H812. RNA2 consists of two hairpins with loop sequences that are very similar to their *E. coli* counterparts. The *T. parva* homologue was detected because the single-stranded loop sequences are identical to those of *P. falciparum*. Assignment of RNA11 was accomplished by searching the mt genomes for the conserved sequence GGUA between H1164 and H812, followed by a sequence that could pair with the appropriate region of LSUA. Candidate sequences were then manually screened for the presence of a hairpin immediately upstream of the GGUA and for the potential to interact with RNA2. Here again the base-pairing potential for these two RNAs was better for the *T. parva* homologues than for *P. falciparum*, providing confirmation of our structure predictions for the known *P. falciparum* transcripts. RNA1 was previously assigned [4] but we have revised its interactions. The 5′ end of RNA1 basepairs with the 3′ end of RNA11, forming H1196. RNA1 also forms H579 with the 5′ end of LSUA, encompasses H1276, and basepairs with RNA3. It was not possible to predict a structure for the 3′ end of RNA1 or the 5′ end of RNA3. Of the 34 inter and intra base pairs in RNA2, RNA11, and RNA1, six are category 1, and six are category 2 (Figure S10; Table S8).

Part of the 5′ half of RNA3 forms a helix with the 3′ half of RNA1 to form H1295 (Figure 3). RNA3 also bridges the 5′ half to the 3′ half of the LSU rRNA (Figures 3, 4). Part of the 3′ half of RNA3 forms the 5′ strand of the H1648 helix, while the middle section of LSUE forms the 3′ half of this helix (Figure 4). RNA3 is highly conserved among the *Plasmodium* species, varying in only 3–5 sites, and the helical region is 100% conserved for both RNA3 and LSUE. The corresponding *T. parva* sequence (Table 1) partially overlaps the previously predicted LSU5 rRNA of Kairo *et* *al.* [23] and provides compensating base change evidence in support of the proposed interactions. While no covariations exist in the seven basepairs in H1295 (RNA3/RNA1), two category 1 and three category 2 basepairs occur in the 15 basepairs in H1648 (Figures S10, S11; Table S8).

RNA13 is also assigned to the 3′ half of LSU rRNA and corresponds to the previously proposed LSU2 RNA in *T. parva* [23]. This is a short but highly conserved sequence with only one difference among the *Plasmodium* species, at the 3′ end. The fragment corresponds to *E. coli* sequences from ∼2236–2264. The 5′ end forms a shortened helix H2077 with the 3′ end of LSUE and also encompasses helix H2246 (Figure 4). The sequence of H2246 is identical among the *Plasmodium* species, including departure from the consensus sequence at three of six highly conserved positions in and adjacent to the loop. One category 2 base pair occurs in the H2077 helix, and no covariations exist in H2246 (Figure S11; Table S8).

Pairwise comparisons between the *P. falciparum* and *T. parva* mt genomes and the *E. coli rrnB* operon also identified another short conserved sequence (positions 4964–4981 in *P. falciparum*, 1518-1501 in *T. parva*) that resembles *E. coli* 23S rRNA positions 2687-2710, comprising a portion of H2675 (Figure 4). This 27 nt region is identical in all 200 *Plasmodium* species except for one position each for *P. juxtanucleare* and *P. fragile*. RNA18 has been mapped to this region by 5′ and 3′ RACE. No covariations occur from that portion of RNA18 that forms a five base pair helix (Figure S11; Table S8).

RNA6 is virtually identical in *Plasmodium* species (Table S3). The 5′ portion of RNA6 is assigned to part of the 3′ strand of H2675, basepairing with RNA18 and RNA10 (Figure 4). Of the 20 basepairs in RNA6 that interact with RNA18, RNA10, and itself, three are category 2 covariations (Figure S11; Table S8).

We were unable to assign all of the RNA transcripts to specific regions of the SSU and LSU rRNAs. The 34 transcripts identified here in *P. falciparum* (and previous analysis by Feagin and colleagues [3], [4], [27], [28], [62]), plus the five additional transcripts determined by Raabe *et al.* [29] are listed in Table 1. In total, for *P. falciparum,* 27 of the 34(39) transcripts have been assigned to specific regions of the rRNAs. The other hemosporidians have such well-conserved mt genomes relative to *P. falciparum* that we predict they will have corresponding RNAs. Twenty-six RNAs have been predicted for *T. parva* (Table 1) and similar RNAs are predicted to exist for the other piroplasms. Similarly, 20 regions corresponding to RNA fragments have been identified in the mt genome of the coccidian, *Eimeria* (Table S7).

### Composition of the P. falciparum mt SSU rRNA

The rRNA secondary structures are typically compared to those of *E. coli*, as those are the most studied examples. Significant portions of the SSU and LSU rRNAs primary, secondary, and three-dimensional structural elements are conserved in all known rRNAs. The conservation secondary structure diagrams available at the Gutell lab’s Comparative RNA Web (CRW) Site [http://www.rna.ccbb.utexas.edu/SAE/2B/ConsStruc/] reveal that the mt rRNAs are, as a group, much more variable than the rRNAs within the Archaea, Bacteria, nuclear-encoded Eucarya, and chloroplasts. The mt SSU and LSU rRNAs usually have fewer nucleotides than bacterial rRNAs (Table 2). The mt rRNAs of *C. elegans* are contiguous and highly minimized [36], [55], with its SSU rRNA and LSU rRNAs being just slightly smaller than the aggregate size of the *P. falciparum* mt rRNAs. The correspondence of the *P. falciparum* and *C. elegans* mt rRNAs to each other, and to *E. coli* rRNAs, is indicated in Table S9.

**Table 2:** Relative sizes of mt rRNAs.

Organism	SSU^a^	LSU^a^	total	Accession
*Caenorhabditis elegans* mt	697	953	1650	NC_001328.1
*Leishmania tarentolae* mt	610	1173	1783	NC_000894.1
*Plasmodium falciparum* mt^b^	804	1233	2037	M76611.1
*Homo sapiens* mt	954	1559	2513	NC_012920.1
*Polytomella* mt^b^	979	1556	2535	NC_010357.1
*Chlamydomonas reinhardtii* mt^b^	1200	2419	3619	NC_001638.1
*Reclinomonas americana* mt	1595	2751	4346	NC_001823.1
*Escherichia coli*	1541	2904	4445	NC_000913.2

a sizes are cited in nt.

b rRNAs are fragmented.

Twelve RNA fragments have similarity to regions of SSU rRNA (Table 1, Figure 2). The assignments differ slightly from what we previously proposed [4], reflecting the refinement possible with mapped as opposed to predicted RNA ends, as well as addition of new transcripts identified in this study. RNA8 better matches the 790 loop than did RNA4, which is now unassigned and RNA5 rather than RNA9 is assigned to the 5′ strand of H1303. Together, the twelve fragments assigned to SSU rRNA total 804 nt.

The *P. falciparum* mt rRNAs and the *C. elegans* mt rRNAs are truncated in all of the major structural domains in the SSU and LSU rRNAs. *P. falciparum* SSU rRNA contains helices (H27, H39), the 530 loop region (helices H500, H505, H511), and H122, H240, and H289 in domain I (Figure 2; Table S9), though much of domain I is missing. The *C. elegans* mt SSU rRNA also lacks a large portion of domain I. It shares with *P. falciparum* the well-conserved helices in H27, H39, H500, H505 and H511. It lacks H122, H240, and H289 but, unlike *P. falciparum*, the *C. elegans* mt SSU rRNA has helices H9 and H17. The apex of the 530 loop forms a pseudoknot helix with a few nucleotides in the bulge loop between helices H500 and H511 [72], [73], but the pseudoknot in both *P. falciparum* and *C. elegans* lack strong basepairing potential. This is common among mt rRNAs, however [55]. The 530 loop is one of the most conserved regions of rRNA in both sequence and higher order structure. It is well conserved in the mt rRNA of *Plasmodium*, and closely related species of other hemosporidians, coccidians, and piroplasms.

*P. falciparum* and *C. elegans* SSU rRNAs both include helices H567, H577, H769, and H885 in domain II of the SSU rRNA (Figure 2; Table S9). While *P. falciparum* has H821, *C. elegans* does not, and *C. elegans* has part of H673 and H722 while *P. falciparum* apparently does not have these helices. The 790 loop, the cap of the helix H769, is highly conserved in all known rRNAs, including *P. falciparum.*

Domain III is the most well-represented part of the *P. falciparum* mt SSU rRNA, and there is good correspondence between *P. falciparum* and *C. elegans* (Figure 2; Table S9). This domain contains helices H921, H939, H944, H960, H984, H1047 (only part of it is present in *P. falciparum*), H1303 (with a putative extra helix in *P.* *falciparum*), H1350, H1399 (truncated in both *P. falciparum* and *C. elegans*), and H1506.

Despite roughly similar sizes, the *C. elegans* mt SSU rRNA includes portions of domains I and II that are not represented in the *P. falciparum* mt SSU rRNA model. Two of these helices, H9 and H17, are present in all known SSU rRNAs. This suggests that some of the unassigned small RNAs may correspond to those regions. However, while the *Plasmodium* RNA sequences are highly conserved relative to each other, there is insufficient conservation to the specific regions of the SSU rRNA to assign the remaining *P. falciparum* RNAs with confidence.

### Composition of the P. falciparum mt LSU rRNA

Seven LSU rRNA fragments, LSUA-LSUG, were originally predicted based on sequence similarities to other rRNAs [3]. Later, two additional LSU rRNA fragments were identified, RNA1 and RNA10. [4] In this paper, we report identification of transcripts for LSUB and LSUC, and assignment of RNA2, RNA3, RNA6, RNA11, RNA13, and RNA18 to LSU rRNA. All of these fragments have corresponding sequences in the *T. parva* mt genome (Table 1). The *P. falciparum* mt LSU rRNA currently has 15 small RNAs assigned, ranging from 23 to 190 nt long. Together, they total 1233 nt.

The 5′ half of the LSU rRNA secondary structure is significantly reduced in both *P. falciparum* and *C. elegans* relative to *E. coli* (Figure 3; Table S9). Domain I is missing from both. Helix H563 is between domains I and II in the LSU rRNA and is present in *C. elegans.* Domain III is truncated and unmodelled in *C. elegans* while *P. falciparum* has the domain III helix H1295, and H1276, a helix between domains II and III.

LSUA, LSUB, LSUC, RNA2, and RNA11 are all assigned to domain II of LSU rRNA, as is part of RNA1. As noted above, LSUB and LSUC are assigned to the GTPase center, forming helices H1057, H1087, and H1082 respectively. While nearly all of the sequence deletions relative to the *E. coli* rRNAs occur at the peripheral ends of an existing structural domain, two deletions, both in the LSU rRNA, occur internally. One example of this type of deletion occurs in the helix that links the H991 helix to the GTPase center (Figure 3). Helices H1011 and H1030 are significantly truncated in animal mt genomes [55]. For the *C. elegans* mt LSU rRNA, a few base pairs are predicted for H1030 at the top of the stem, with unpaired nucleotides bridging the gap between the GTPase center and the multi-stem loop below H991. In *P. falciparum,* RNA2 and RNA11 basepair to form the basal helix in the stem (H991) but there is no interaction (*i.e.*, noticeable basepairing potential) between these RNAs and the GTPase center RNAs, LSUB and LSUC. Indeed, while each of the latter can form an intramolecular helix and can potentially interact with each other by forming an extended helix H1082 (not present in *C. elegans*), they are so small that there is no obvious capacity for them to interact with any of the other small RNAs. We hypothesize that the GTPase center RNAs are maintained in proper position by interactions with ribosomal proteins. RNA2 is also assigned to H946 and H976, neither of which is included in the *C. elegans* structure. RNA11 is also assigned to H1164 and provides the 3′ half of the H812 helix while the 5′ half of H812 is derived from LSUA. H1196 is shortened in *P. falciparum*. In contrast, *C. elegans* lacks H1164 and H1196 is also truncated. RNA1 is assigned at the junction of domains II and III, having a strongly conserved AGUA sandwiched between dinucleotides that conserve long-distance basepairing to domain IV.

The 3′ half of the LSU rRNA secondary structure is the most fully represented region, compared to *E. coli*, for both *C. elegans* and *P. falciparum* (Figure 4; Table S9). Parts of Domain IV interact with tRNA and the 30S ribosomal subunit. LSUD and LSUE provide most of domain IV, with RNA3 assigned to one side of helix H1648, the other side being provided by LSUE. There is no *C. elegans* sequence corresponding to RNA3. A set of adjacent helices (H1682, H1707, and H1752) are missing from both species and helix H1835 is reduced in *C. elegans* and *P. falciparum* to a third the size in *E. coli*. Helix 1792 is similar in size in *E. coli* and *P. falciparum* but reduced by half in *C. elegans*. Other features of domain IV are maintained in structure and sequence conservation. LSUD and LSUE are separate RNAs in *P. falciparum* despite the fact that they directly abut each other in the *P. falciparum* mt genome. These two sequences have been found on the same transcript in *T. parva* (RNA1 of Kairo *et al*. [23]), but it is possible that this represents a precursor of LSUD and LSUE. Such a precursor RNA containing LSUD and LSUE is detected in *P. falciparum* by RNase protection (Mericle and Feagin, unpublished results) and by RT-PCR [62].

Structural features of domain V are also strongly conserved between *P. falciparum* and *C. elegans*, which show similar variance from *E. coli* (Figure 4). Both are missing the L1 binding domain (H2112, H2113, H2117, H2120, and H2127) and the underlying H2093 and H2200 helices. The 5S rRNA binding domain (helices H2283–H2372) is deleted in both *C. elegans* and *P. falciparum.* The bulk of domain V, including the peptidyltransferase center, is intact and well-conserved, comprised of LSUE, LSUF, and LSUG.

The most conserved feature of domain VI is the sarcin ricin loop at the tip of the H2646 helix. This hairpin loop is conserved across all 3P2O LSU rRNA genes, including the *P. falciparum* mt RNA10 fragment (Figure 4). The initial 13 nt of RNA10 are identical among *Plasmodium* species and hemosporidians but have not been assigned to a structure. The next 38 nt are variable across *Plasmodium* species and include alignment gaps but within the more closely related subgroups, conservation is strong (Figure S1). Regardless of the variation, this sequence can form a long helix (Figure 4; 17 basepairs with an AAUU tetraloop in *P. falciparum*). There is no corresponding structure in *C. elegans* or *E. coli* but one has been proposed in the mt rRNA of the dinoflagellate *Karlodinium micrum* [74]. If a similar hairpin exists in *T. parva* RNA10, it is much shorter and significantly less stable. The 3′ end of RNA10 and the 5′ end of RNA18 comprise the 5′ strand of H2675. The 3′ end of RNA18 continues to begin the 3′ strand of H2675, and the rest of this strand is present in the 5′ end of RNA6 which continues to form H2735. Domain VI is also truncated in *C. elegans*, with strong correspondence in the regions that are present and absent in *P. falciparum*.

As with the SSU rRNA, it is tempting to speculate that the regions in *C. elegans* mt LSU rRNA that are missing from the assigned RNAs for the *P. falciparum* mt LSU rRNA are among the unassigned RNAs.

As noted above, our confidence in the placement of the RNA fragments onto the SSU and LSU rRNA secondary structures is augmented with covariations between the two nucleotides that form basepairs. Covariation analysis facilitates the identification of the base pairs in an RNA secondary structure that are similar among the sequences in the alignment dataset. When the alignments juxtapose similar structural units in each column, and they are properly interpreted with covariation analysis, the predicted structure is nearly 100% accurate [75]. Two different *P. falciparum* datasets were analyzed for positional covariations. The first compares the RNAs with conservation of the 3P2O dataset. Nucleotides that are conserved in 90–98% of the 3P2O dataset in Figures 2, 3, 4 are shown as a lower case red or blue letter, and those nucleotides conserved in 98–100% of the same 3P2O dataset are shown as an upper case red or blue letter. Red indicates that the *P. falciparum* sequence is the same as the conserved 3P2O sequence while blue indicates that it is different. Seven base pairs in the SSU and LSU rRNAs secondary structure diagrams have both nucleotides colored blue indicating that the *P. falciparum* sequence has covariation at some of the most conserved nucleotides in the rRNA (Figures 2, 3, 4).

The second dataset contains the predicted mt rRNAs of 218 species of *Plasmodium* species and related apicomplexans. These covariations are shown on the SSU and LSU secondary structure diagrams (Figures S9, S10, S11; Table S8). The total number of base pairs that have a covariation between the two paired nucleotides are: SSU rRNA, 10 category 1 and 21 category 2; LSU rRNA, 42 category 1 and 39 category 2; total rRNA, 52 category 1 and 60 category 2. While several helices do not have any covariations, the majority of the helices contain at least one. Thus, we are very confident that the majority, if not all, of the *P. falciparum* fragments, have been accurately mapped to the rRNA secondary structure. An analysis of a more diverse collection of apicomplexan mt genomes and their RNA transcripts would further evaluate the accuracy of fragment mapping to the rRNAs in this manuscript, and likely facilitate the placement of a few more fragments.

### Predicted Structure of the P. falciparum mt Ribosome

In recent years, the crystal structures of large and small ribosomal subunits and of whole ribosomes have been obtained from several sources, greatly refining our understanding of ribosome function during protein synthesis. Of particular interest to the work reported here is structural data from the *C. elegans* mt ribosome [36], [55]. Like *P. falciparum*, *C. elegans* has highly minimized rRNAs and as discussed above, the *P. falciparum* mt rRNA fragments correspond closely to the *C. elegans* mt rRNAs. Mt ribosomes have a larger protein to RNA ratio but the polypeptide exit tunnel is still lined entirely with RNA [34]. While nothing is specifically known about the protein content of *P. falciparum* mt ribosomes, all of the RNA sequences that comprise the lining of the *C. elegans* mt ribosome polypeptide exit tunnel are represented in the RNAs that have been mapped to the 3′ portion of *P. falciparum* mt LSU rRNA.

**Figure 5:**
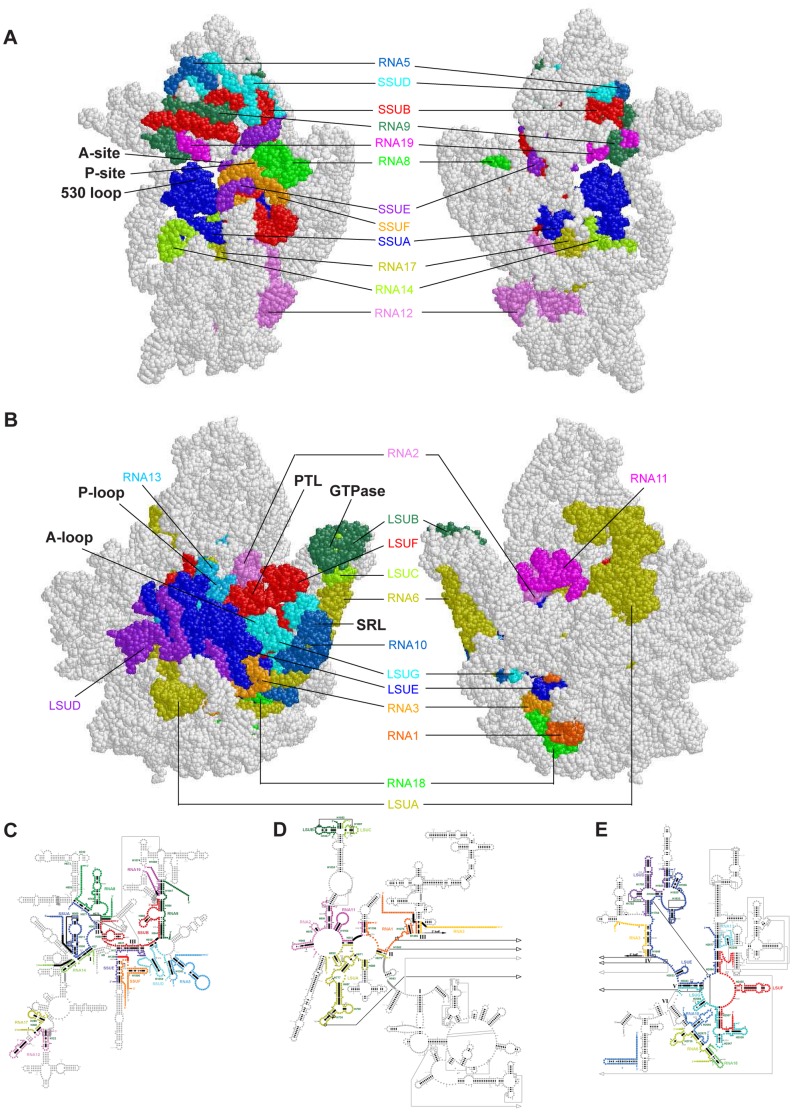
rRNA tertiary structure. The *P. falciparum* mt rRNAs are superimposed on a space-filling model of the three dimensional rRNA structure. Each individual *P. falciparum* rRNA fragment is colored and labeled; regions of the model with no *P. falciparum* equivalent are colored gray. Functional regions of the rRNAs are labeled in black. Full-page versions of each panel are available as Figures S12–S16. (**A**) *P. falciparum* SSU rRNA superimposed on *Thermus thermophilus* (PDB ID 1J5E; left  =  front/interface side, right  =  back); (**B**) *P. falciparum* LSU rRNA superimposed on *Haloarcula marismortui* (PDB ID 1S72; left  =  crown/interface side, right  =  back); (**C–E**) secondary structure diagrams for SSU, 5′ LSU, and 3′ LSU rRNAs, respectively, with each *P. falciparum* mt rRNA fragment color-coordinated with the fragment colors in the three-dimensional structure.

The high-resolution three-dimensional crystal structures of the 30S [31] and 50S [30] ribosomal subunits revealed that the comparative secondary structure models for the SSU and LSU rRNA were 97–98% accurate [75]. These crystal structures also revealed the detailed three-dimensional folding of the secondary structure. We wanted to determine if the fragments of *P. falciparum* rRNA are close together in three-dimensional space and if they were at the known functional sites of the ribosome in protein synthesis. All of the fragments that have been assigned to the SSU and LSU rRNA secondary structure (Figures 2, 3, 4) were mapped onto the three-dimensional structure of the ribosome (Figure 5A–E; full-page versions in Figures S12, S13, S14, S15, S16). These figures reveal that indeed nearly all of the *P. falciparum* rRNA fragments map to the regions of the ribosome known to be directly involved in protein synthesis.

## Discussion

The *P. falciparum* mt genome encodes 34 small RNAs, 27 of which have been assigned as parts of fragmented LSU and SSU rRNAs. Compared to other fragmented rRNAs, the number of separate RNAs is much larger while the fragments are far smaller, some containing only 23 nt. Are these tiny RNAs in fact part of functional ribosomes? Considerable data now supports this hypothesis.

Some of the *Plasmodium* mt rRNA fragments were first proposed because of sequence similarities to rRNA regions [3], [20], [76]. A key issue became whether the predicted rRNAs corresponded to real transcripts. For a few, the answer was no [4], but the small RNAs discussed here are, by definition, transcribed. The mt genome is polycistronically transcribed [62]; larger presumed precursor RNAs can be detected by RNase protection [62], RT-PCR [62], and in 3′ and 5′ RACE analysis (this work). The small RNAs persist in the cell while non-coding mt sequences appear to be rapidly degraded, based on our inability to detect them other than by RT-PCR of presumed precursor transcripts.

The small RNAs are highly conserved across species and between genera. Comparison of the mt genomes of 26 *Plasmodium* species shows strong conservation of nucleotide sequence for the small RNAs, to the degree seen for amino acid sequences of the three mt protein coding genes (Table S3). Mt genome size differences between the *Plasmodium* species predominantly reflect addition or deletion of sequences in non-coding regions or at junctions between RNAs. Sequences corresponding to many of the *P. falciparum* rRNA fragments can be found in mt genomes of other apicomplexans (Table 1; Table S3); some of them have been confirmed as transcripts for *T. parva* [23]. Analysis of dinoflagellate mt sequences has provided evidence that they also have highly fragmented mt rRNAs [74], [77], [78]. It has so far been impossible to strictly define the genetic content of the dinoflagellate mt genome. It typically exists as multiple genome molecules carrying differing combinations of the mt genome, including different pseudogenes for the protein coding genes *cob*, *cox1* and *cox3*. These often show terminal deletions as if the individual DNAs are subject to a high rate of recombination. The complex nature of dinoflagellate mt genomes means that none can be considered complete. However, sequences corresponding to rRNAs have been reported for several species. Those described to date correspond to some of the best-conserved *P. falciparum* mt rRNA fragments, not only in the general identity of the region but also the size and fragmentation pattern. Dinoflagellates are close relatives to apicomplexans so these data suggest that the alveolate that is ancestral to both groups also possessed fragmented mt rRNAs.

Seven of the small RNAs have not been assigned to regions of rRNA. Their characteristics are otherwise similar to the rRNA fragments, however. They are highly conserved among *Plasmodium* species, are in a similar size range, and have non-encoded oligo(A) tails. We hypothesize that at least some, perhaps all, of them correspond to regions that are less well-conserved among rRNAs, especially regions present in the *C. elegan*s mt rRNA but missing from *P. falciparum* rRNAs. The greatest discrepancies between *P.* *falciparum* and *C. elegans* lie in SSU rRNA, suggesting that a number of the unassigned RNAs have a role there. However, alternative scenarios cannot be ruled out, including possible non-rRNA functions. In that regard, it is important to note that none of the small RNAs has a size or potential secondary structure suggestive of tRNAs.

Mt rRNAs are often small compared to bacterial rRNAs but contain key regions important to function. When arrayed in positions corresponding to the secondary structure of canonical rRNAs, the *P. falciparum* mt rRNA fragments correspond quite well to regions present in other small mt rRNAs. In particular, correspondence to the very small *C. elegans* mt rRNAs is quite high. All of the rRNA fragments are predicted to basepair with at least one additional rRNA, creating interactions that should help keep the individual RNAs properly located and oriented. Mt ribosomal proteins likely play a key role in maintaining the relationships of the RNAs to each other since the interactions expected between some fragments are limited.

Arraying the rRNA fragments in a 3-D model further substantiates the picture of a small but functional ribosome with the rRNA fragments clustered mostly on the interface between subunits. The critical point is that nearly all regions of the rRNAs that are associated with protein synthesis/function are present in the *Plasmodium* mt rRNAs. Despite their unconventional characteristics, they appear likely to function conventionally once assembled into ribosomes. How that is mediated and the role of the unassigned fragments both remain to be determined.

## Materials and Methods

### Parasites

The C10 clone of *P. falciparum* was grown *in vitro* by the method of Trager and Jensen [79]. Parasites were harvested by saponin lysis of infected red cells, followed by centrifugation and washing. Harvested cells were quick-frozen in liquid nitrogen and stored at −80°C until use.

### DNA Analysis

Complete mt DNA sequences from 25 *Plasmodium* species and an isolate from a mandrill, plus eight other hemosporidians (Table S1) were aligned in the AlignX program of Vector NTi X10 using the Clustal W function, and manually revised as needed to conform to the mapped 5′ and 3′ ends of *P. falciparum* RNAs. Using this alignment, the sequence conservation relative to *P. falciparum* was determined in each species for each of the small RNAs and for the intergenic regions. Intergenic regions showing an average conservation of ∼90% or better were examined on both DNA strands for the presence of additional small RNAs by RNA blotting.

### RNA Preparation and Analysis

Total RNA was prepared as previously described [4] by the acid-guanidinium-phenol-chloroform method of Chomzynski and Sacchi [80] or with Trizol (Invitrogen). RNA blots were prepared and analyzed as previously described [4], [61]. Briefly, total RNA was electrophoresed on 12%, 16%, or 20% acrylamide, 7 M urea gels in TBE (0.1 M Tris-borate, 0.9 mM EDTA, pH 8.3) and electrophoretically transferred to nylon membrane in TAE (40 mM Tris-acetate, 1 mM EDTA, pH 8.2). Probes for the RNA blots were [^32^P]-labeled in vitro transcripts complementary to the gene or [^32^P]-end-labeled oligonucleotides, with hybridization and wash conditions as previously described for similar probes [4]. Primer extension experiments were performed as previously described [81], using unlabeled oligonucleotide primers (Table S4) and Superscript reverse transcriptase (Invitrogen) in the presence of [α-^32^P] dATP. 3′ RACE was employed to determine the 3′ end of the transcripts. Total RNA was treated with DNase I (Pharmacia/Amersham) in the presence of RNasin (Promega), followed by the addition of a 3′ C tail with poly(A) polymerase (Invitrogen), as previously described [28]. First strand cDNA was prepared using an oligo(dG) primer (CGGGATCCGGGGGGGGGGGG) and Superscript reverse transcriptase. It was then PCR amplified using Pfx Platinum polymerase (Invitrogen), with the oligo(dG) primer and a gene-specific 5′ primer (Table S4) for each rRNA fragment. PCR conditions were 2 min at 95°C, followed by 4 repetitions of 30 seconds at primer-specific annealing temperature, one min at 72°C, 15 sec at 95°C, then 29 repetitions of the same with the annealing temperature raised to 58°C, and finally 10 min at 72°C. For 5′ RACE, first strand cDNA was prepared with gene-specific primers, a C tail was added to the cDNA using terminal deoxynucleotidyl transferase, and PCR was carried out using a nested gene-specific primer and the oligo(dG) primer. For some RNAs, the size or sequence of the transcripts precluded making a nested primer so the cDNA primer was used for PCR as well, with mixed success. The PCR products were cloned into the pGEM T-EZ vector (Promega) and sequenced using an Applied Biosystems 3730XL Genetic Analyzer.

### Assignment of P. falciparum mt Transcripts to Regions of rRNA Secondary Structure Models

The *P. falciparum* and *T. parva* mt genome sequences were re-analyzed by performing pairwise BLAST comparisons [82] between these two sequences and between each of these and the *E. coli rrnB* operon [83]. Mt rRNA homologs detected in this way were then manually folded to fit the *E. coli* secondary structure models (http://www.rna.ccbb.utexas.edu/) [55], taking the *P. falciparum* transcript mapping data into consideration. The Spin program of the Staden package [84] was then used to assign transcripts to "missing" regions of the secondary structures; we searched for short stretches of sequence that are known to be conserved among other rRNAs (see structure conservation diagrams at the CRW Site). In some searches, we also included query sequences that were predicted by the base pairing partner sequence contained within other rRNA fragments that were already assigned to the secondary structure models.

### 3D Model

The computer program RasMol [85] and its scripting language were used to color the regions of the SSU and LSU rRNA that are associated with the *P. falciparum* rRNA fragments.

### Accession Numbers

Accession numbers for sequences are found in Table S1. All are mitochondrial genome sequences from apicomplexan parasites.

## Supporting Information

Figure S1 **RNA10 variation among** ***Plasmodium*** **species.** The sequence of *P. falciparum* RNA10 is shown in the top line, and the sarcin ricin loop sequence is indicated with a horizontal bracket. For the other *Plasmodium* species, positions that differ from *P. falciparum* are indicated with letters. Bases shown in red are conserved in all *Plasmodium* species., conserved relative to *P. falciparum*; -, no corresponding nucleotide.
(TIF)

Figure S2 **Identification of** ***P. falciparum*** **mitochondrial GTPase center rRNAs. (A)** The predicted secondary structures of the most conserved portions of the *P. falciparum* mt sequence corresponding to the GTPase center are shown. Strongly conserved nt are shown in color, with lower case indicating 90%+ and upper case indicating 95%+ conservation among three domains and two organelles. Red nt in the *P. falciparum* sequence match the consensus and the blue nt differs from consensus. Open circles are placeholders to indicate the structure of a conventional GTPase center. These sequences are identical among 26 different species of *Plasmodium,* with the exception of the single position in LSUB that varies from overall consensus. **(B)** Total *P. falciparum* RNA was electrophoresed on denaturing 7M urea, 20% acrylamide gels, electrophoretically transferred to nylon membrane, and probed with radiolabeled oligonucleotides complementary to LSUB and LSUC (Table S4). Transcript sizes were determined using a ladder of small *in vitro* transcripts as markers.
(TIF)

Figure S3 **Small mitochondrial RNAs of** ***P. falciparum*** **.** Total *P. falciparum* RNA was electrophoresed on denaturing 7M urea, 12% acrylamide gels, electrophoretically transferred to nylon membrane, and probed with radiolabeled sequences (Table S4) complementary to rDNA regions from the *P. falciparum* mt genome. Transcript sizes were determined using a ladder of small *in vitro* transcripts as markers. The identities of transcripts shown on the same panel (RNA11, SSUD) were separately established with oligonucleotide probes. Abbreviations are as shown for Figure 1.
(TIF)

Figure S4 **LSUF analysis. (A)** Total *P. falciparum* RNA was electrophoresed on denaturing 7M urea, 12% acrylamide gels, electrophoretically transferred to nylon membrane, and probed with a radiolabeled *in vitro* transcript complementary to LSUF. Transcript sizes, given in nt, were determined using a ladder of small *in vitro* transcripts as markers. **(B)** Total *P. falciparum* RNA (10 ng/lane) was incubated with an unlabeled oligonucleotide primer and Superscript reverse transcriptase (Invitrogen) in the presence of [α-^32^P] dATP. The products were electrophoresed on 7M urea, 12% acrylamide gels and exposed to film. Lanes 1–3, extensions were performed at 50°C, 55°C, and 60°C, respectively. The position of the transcript ends is indicated relative to the *P. falciparum* mt genome (Genbank M76611). **(C)** The *P. falciparum* mt sequence for nt 1501–1530 is shown. Vertical arrows indicate the two mapped 5′ ends for LSUF. Lower case letters indicate positions that vary among 200 *P. falciparum sequences*. The number of hemosporidian species exhibiting the specific nt at each position is given.
(TIF)

Figure S5 **Mapping** ***P. falciparum*** **mt RNAs.** Schematic representations are shown for two regions of the *P. falciparum* mt genome, with mapped RNAs indicated by boxes and the DNA sequence for transcript ends shown above the boxes. Transcript termini are in bold, with the 5′ nt italicized and the 3′ nt underlined. The nt position in the genome is located above each terminus.
(TIF)

Figure S6 **Proposed secondary structure for** ***T. parva*** **mt SSU rRNA.** The *T. parva* mt SSU rRNA secondary structure model is superimposed onto the *E. coli* SSU rRNA secondary structure model diagram. The transcript ends shown are artificial, based on the extent of sequence similarity between the mapped *P. falciparum* RNAs, and the *T. parva* mt DNA sequence, or the capacity to form expected secondary structure.
(TIF)

Figure S7 **Proposed secondary structure for** ***T. parva*** **mt LSU** **rRNA (5′ half).** The *T. parva* mt LSU rRNA (5′ half) secondary structure model is superimposed onto the *E. coli* LSU rRNA (5′ half) secondary structure model diagram. The transcript ends shown are artificial, based on the extent of sequence similarity between the mapped *P. falciparum* RNAs, and the *T. parva* mt DNA sequence, or the capacity to form expected secondary structure.
(TIF)

Figure S8 **Proposed secondary structure for** ***T. parva*** **mt LSU** **rRNA (3′ half).** The *T. parva* mt LSU rRNA (3′ half) secondary structure model is superimposed onto the *E. coli* LSU rRNA (3′ half) secondary structure model diagram. The transcript ends shown are artificial, based on the extent of sequence similarity between the mapped *P. falciparum* RNAs, and the *T. parva* mt DNA sequence, or the capacity to form expected secondary structure.
(TIF)

Figure S9 **Location of type 1 and 2 covariations in SSU rRNA.** The *P. falciparum* mt SSU rRNA secondary structure model, shown in thick gray lines, is superimposed onto the *E. coli* SSU rRNA secondary structure model diagram. Regions where *P. falciparum* has no structure equivalent to *E. coli* are shown using gray circles and lines. Category 1 covarying pairs are indicated with red and category 2 with blue.
(TIF)

Figure S10 **Location of type 1 and 2 covariations in LSU rRNA (5′ half).** The *P. falciparum* mt LSU rRNA (5′ half) secondary structure model, shown in thick gray lines, is superimposed onto the *E. coli* LSU rRNA (5′ half) secondary structure model diagram. Regions where *P. falciparum* has no structure equivalent to *E. coli* are shown using gray circles and lines. Category 1 covarying pairs are indicated with red and category 2 with blue.
(TIF)

Figure S11 **Location of type 1 and 2 covariations in LSU rRNA (3′ half).** The *P. falciparum* mt LSU rRNA (3′ half) secondary structure model, shown in thick gray lines, is superimposed onto the *E. coli* LSU rRNA (3′ half) secondary structure model diagram. Regions where *P. falciparum* has no structure equivalent to *E. coli* are shown using gray circles and lines. Category 1 covarying pairs are indicated with red and category 2 with blue.
(TIF)

Figure S12 **SSU rRNA tertiary structure.** The *P. falciparum* mt SSU rRNAs are superimposed on a space-filling model of the three dimensional rRNA structure of *Thermus thermophilus* (PDB ID 1J5E; left  =  front/interface side, right  =  back). Each individual *P. falciparum* rRNA fragment is colored and labeled; regions of the model with no *P. falciparum* equivalent are colored gray. Functional regions of the rRNAs are labeled in black. (Full page version of Figure 5A.)
(TIF)

Figure S13 **LSU rRNA tertiary structure.** The *P. falciparum* LSU rRNA is superimposed on a space-filling model of the three dimensional rRNA structure of *Haloarcula marismortui* (PDB ID 1S72; left  =  crown/interface side, right  =  back). Each individual *P. falciparum* rRNA fragment is colored and labeled; regions of the model with no *P. falciparum* equivalent are colored gray. Functional regions of the rRNAs are labeled in black. (Full page version of Figure 5B.)
(TIF)

Figure S14 ***P. falciparum*** **mt SSU rRNAs color-coded to tertiary structure.** Secondary structure diagram for *P. falciparum* mt SSU rRNA with each *P. falciparum* mt rRNA fragment color-coordinated with the fragment colors in the *Thermus thermophilus* three-dimensional structure (Figure S12). (Full page version of Figure 5C.)
(TIF)

Figure S15 ***P. falciparum*** **mt LSU rRNAs (5′ half) color-coded to tertiary structure.** Secondary structure diagram for *P. falciparum* mt LSU rRNA (5′ half) with each *P. falciparum* mt rRNA fragment color-coordinated with the fragment colors in the *Haloarcula marismortui* three-dimensional structure (Figure S13). (Full page version of Figure 5D.)
(TIF)

Figure S16 ***P. falciparum*** **mt LSU rRNAs (3′ half) color-coded to tertiary structure.** Secondary structure diagrams for *P. falciparum* mt LSU rRNA (3′ half) with each *P. falciparum* mt rRNA fragment color-coordinated with the fragment colors in the *Haloarcula marismortui* three-dimensional structure (Figure S13). (Full page version of Figure 5E.)
(TIF)

Table S1 **Mt genomes from** ***Plasmodium*** **species and related apicomplexans.**
(PDF)

Table S2 **Intergenic size variation among hemosporidian mt genomes.**
(PDF)

Table S3 **Similarity of individual mt genes from** ***Plasmodium*** **species relative to** ***P. falciparum.***
(PDF)

Table S4 **Oligonucleotides used in this study.**
(PDF)

Table S5 ***P. falciparum*** **mt rRNA 3′ end sequences.**
(PDF)

Table S6 **Abutted** ***P. falciparum*** **mt gene sets.**
(PDF)

Table S7 **Fragment placement in rRNA alignments for covariation analysis.**
(PDF)

Table S8 **Prospective covariations found in fragmented apicomplexan mt rRNA genes.**
(PDF)

Table S9 **Correspondence of** ***P. falciparum*** **and** ***C. elegans*** **mt rRNAs.**
(PDF)
