# Supplementary figures and images for: The Fragmented Mitochondrial Ribosomal RNAs of Plasmodium falciparum
Source: PLoS One. 2012 Jun 22;7(6):e38320. doi: 10.1371/journal.pone.0038320 (PMC3382252; doi:10.1371/journal.pone.0038320)

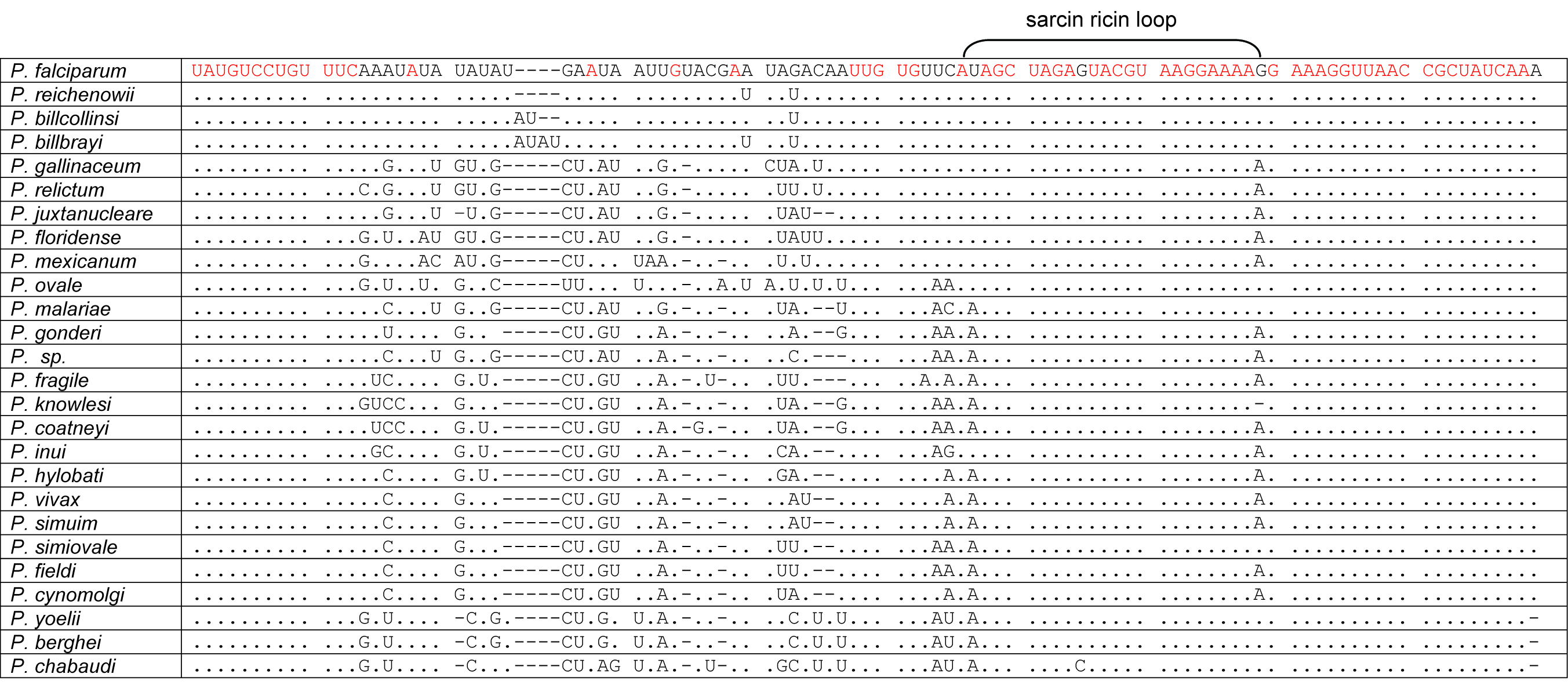

Supplement: Figure S1 — RNA10 variation among Plasmodium species. The sequence of P. falciparum RNA10 is shown in the top line, and the sarcin ricin loop sequence is indicated with a horizontal bracket. For the other Plasmodium species, positions that differ from P. falciparum are indicated with letters. Bases shown in red are conserved in all Plasmodium species., conserved relative to P. falciparum; -, no corresponding nucleotide. (TIF) [file pone.0038320.s001.tif]

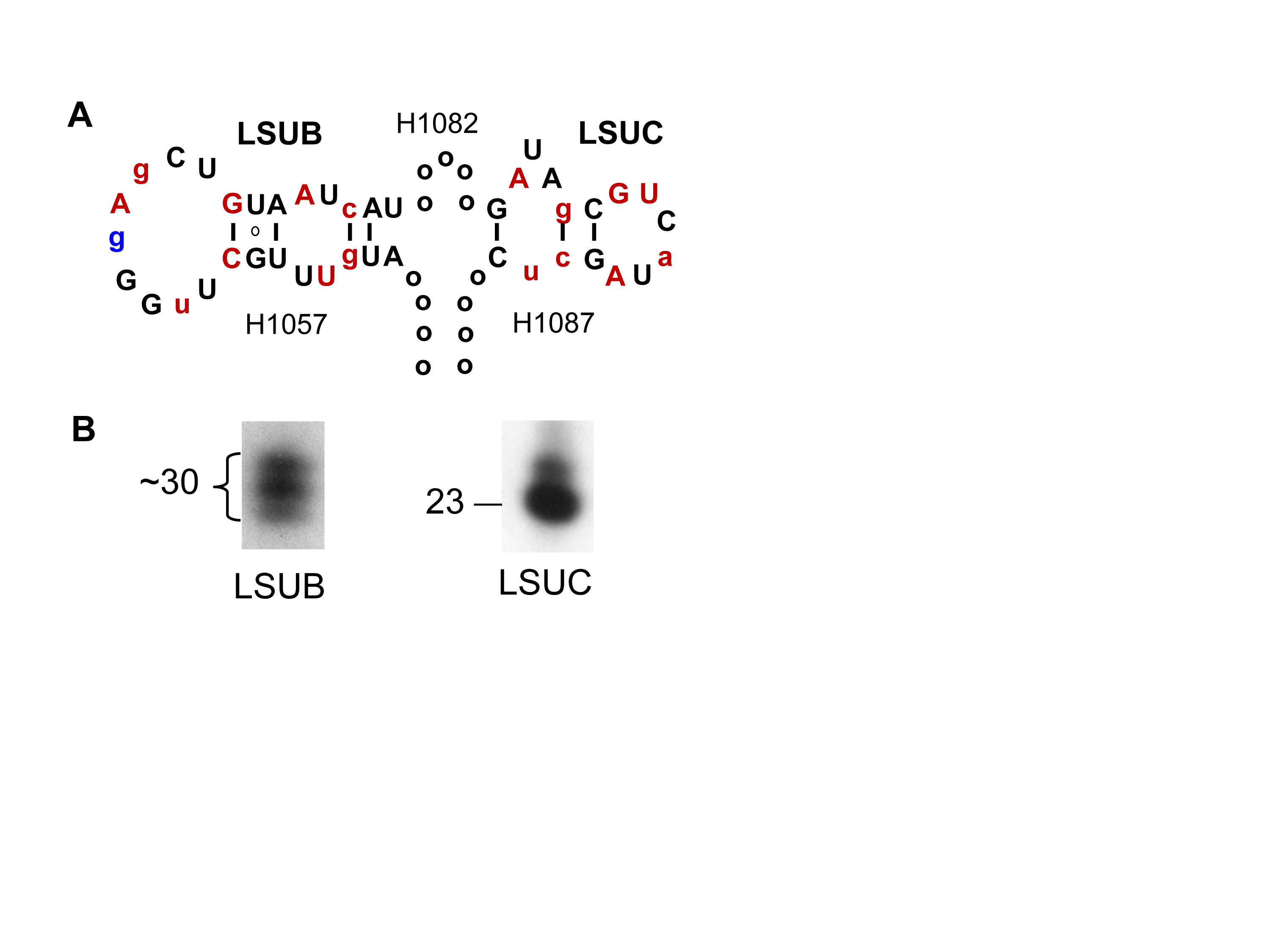

Supplement: Figure S2 — Identification of P. falciparum mitochondrial GTPase center rRNAs. (A) The predicted secondary structures of the most conserved portions of the P. falciparum mt sequence corresponding to the GTPase center are shown. Strongly conserved nt are shown in color, with lower case indicating 90%+ and upper case indicating 95%+ conservation among three domains and two organelles. Red nt in the P. falciparum sequence match the consensus and the blue nt differs from consensus. Open circles are placeholders to indicate the structure of a conventional GTPase center. These sequences are identical among 26 different species of Plasmodium, with the exception of the single position in LSUB that varies from overall consensus. (B) Total P. falciparum RNA was electrophoresed on denaturing 7M urea, 20% acrylamide gels, electrophoretically transferred to nylon membrane, and probed with radiolabeled oligonucleotides complementary to LSUB and LSUC (Table S4). Transcript sizes were determined using a ladder of small in vitro transcripts as markers. (TIF) [file pone.0038320.s002.tif]

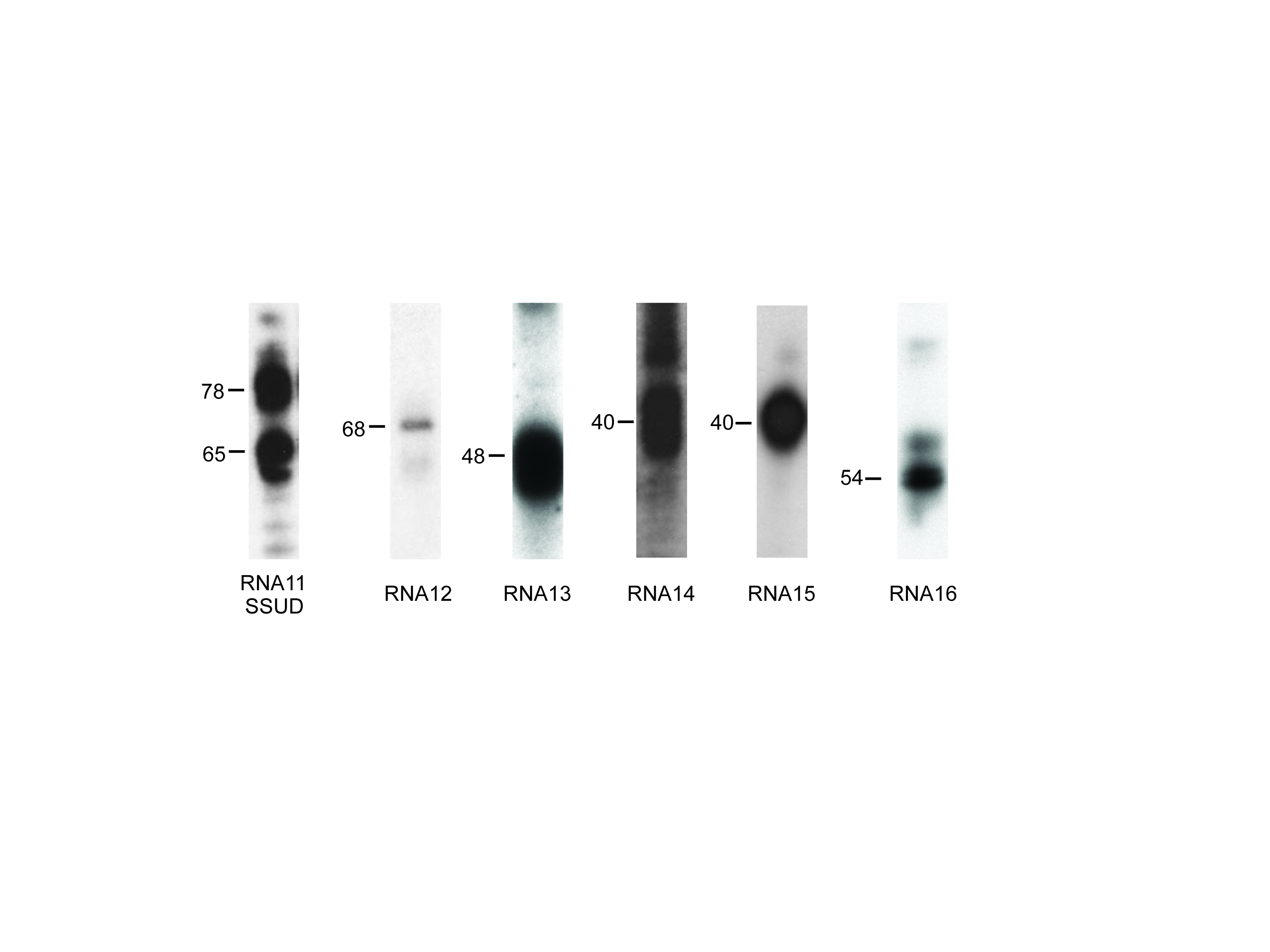

Supplement: Figure S3 — Small mitochondrial RNAs of P. falciparum . Total P. falciparum RNA was electrophoresed on denaturing 7M urea, 12% acrylamide gels, electrophoretically transferred to nylon membrane, and probed with radiolabeled sequences (Table S4) complementary to rDNA regions from the P. falciparum mt genome. Transcript sizes were determined using a ladder of small in vitro transcripts as markers. The identities of transcripts shown on the same panel (RNA11, SSUD) were separately established with oligonucleotide probes. Abbreviations are as shown for Figure 1. (TIF) [file pone.0038320.s003.tif]

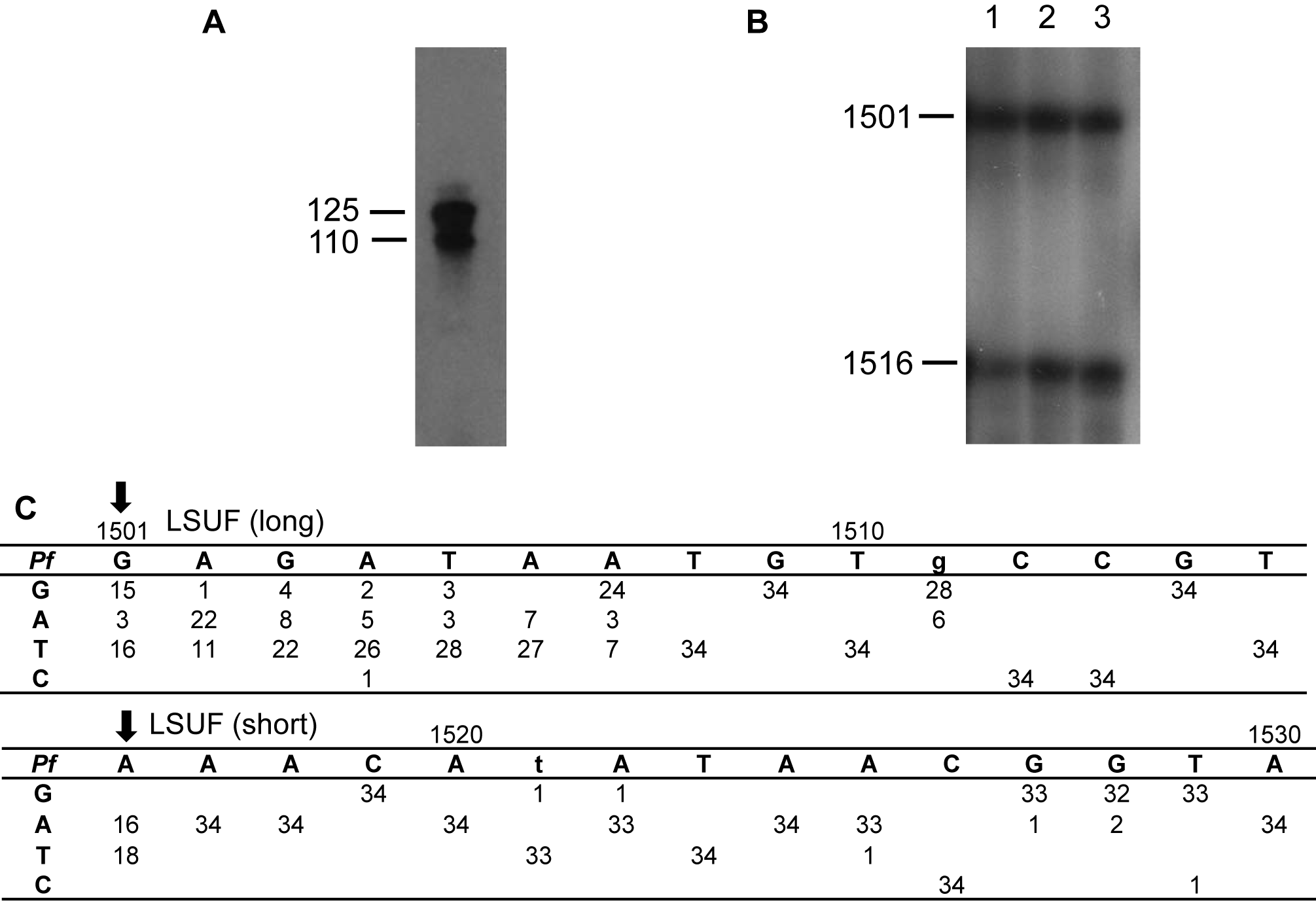

Supplement: Figure S4 — LSUF analysis. (A) Total P. falciparum RNA was electrophoresed on denaturing 7M urea, 12% acrylamide gels, electrophoretically transferred to nylon membrane, and probed with a radiolabeled in vitro transcript complementary to LSUF. Transcript sizes, given in nt, were determined using a ladder of small in vitro transcripts as markers. (B) Total P. falciparum RNA (10 ng/lane) was incubated with an unlabeled oligonucleotide primer and Superscript reverse transcriptase (Invitrogen) in the presence of [α-32P] dATP. The products were electrophoresed on 7M urea, 12% acrylamide gels and exposed to film. Lanes 1–3, extensions were performed at 50°C, 55°C, and 60°C, respectively. The position of the transcript ends is indicated relative to the P. falciparum mt genome (Genbank M76611). (C) The P. falciparum mt sequence for nt 1501–1530 is shown. Vertical arrows indicate the two mapped 5′ ends for LSUF. Lower case letters indicate positions that vary among 200 P. falciparum sequences. The number of hemosporidian species exhibiting the specific nt at each position is given. (TIF) [file pone.0038320.s004.tif]

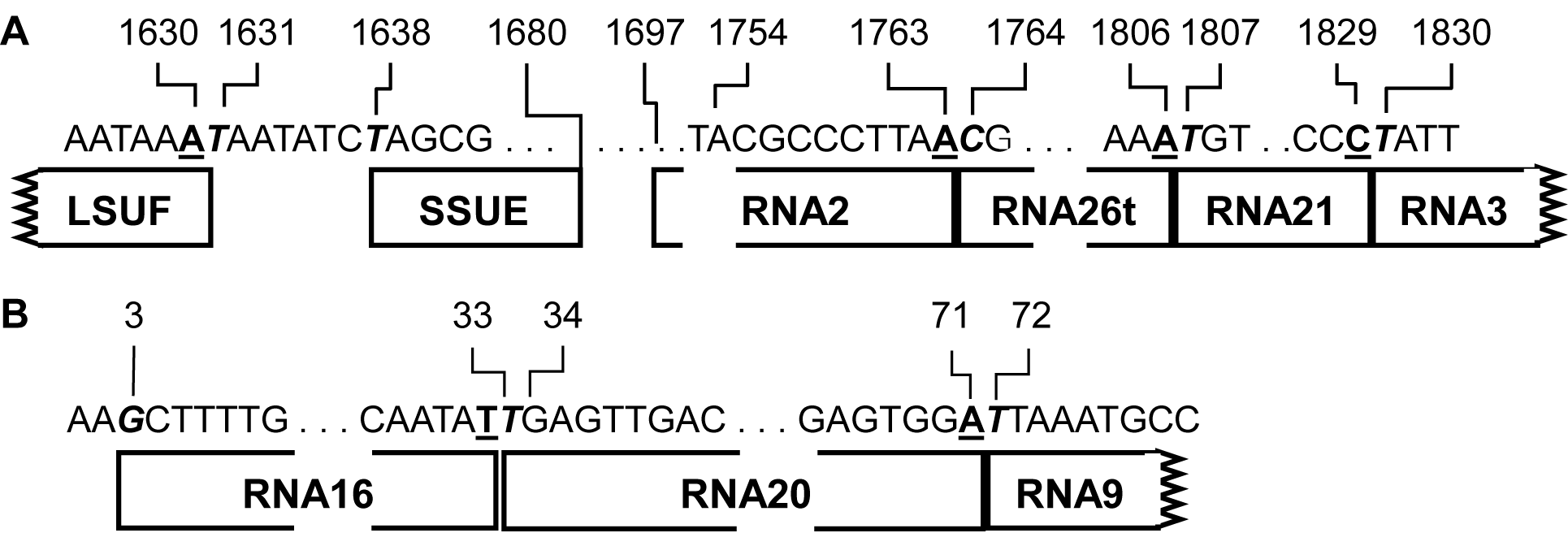

Supplement: Figure S5 — Mapping P. falciparum mt RNAs. Schematic representations are shown for two regions of the P. falciparum mt genome, with mapped RNAs indicated by boxes and the DNA sequence for transcript ends shown above the boxes. Transcript termini are in bold, with the 5′ nt italicized and the 3′ nt underlined. The nt position in the genome is located above each terminus. (TIF) [file pone.0038320.s005.tif]

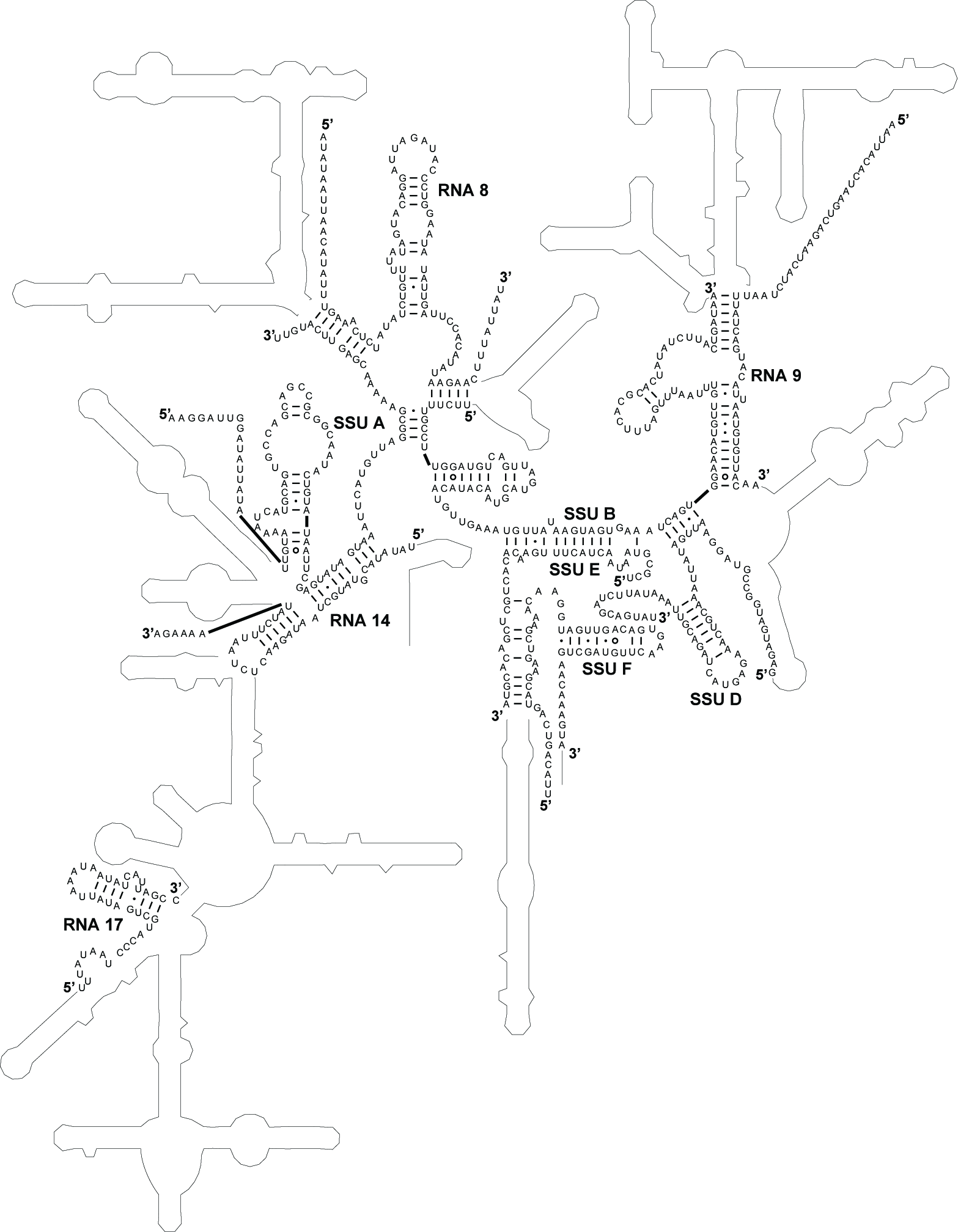

Supplement: Figure S6 — Proposed secondary structure for T. parva mt SSU rRNA. The T. parva mt SSU rRNA secondary structure model is superimposed onto the E. coli SSU rRNA secondary structure model diagram. The transcript ends shown are artificial, based on the extent of sequence similarity between the mapped P. falciparum RNAs, and the T. parva mt DNA sequence, or the capacity to form expected secondary structure. (TIF) [file pone.0038320.s006.tif]

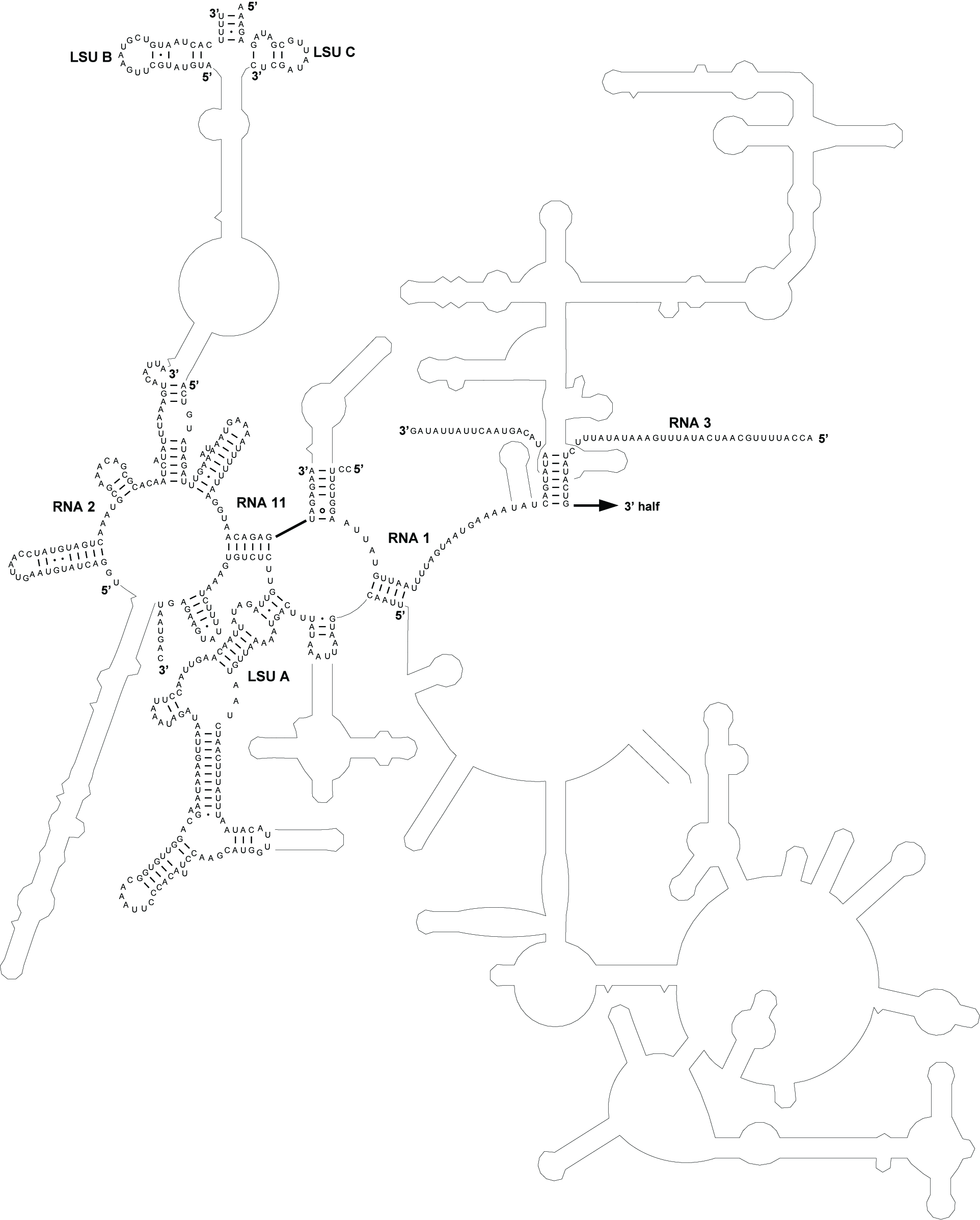

Supplement: Figure S7 — Proposed secondary structure for T. parva mt LSU rRNA (5′ half). The T. parva mt LSU rRNA (5′ half) secondary structure model is superimposed onto the E. coli LSU rRNA (5′ half) secondary structure model diagram. The transcript ends shown are artificial, based on the extent of sequence similarity between the mapped P. falciparum RNAs, and the T. parva mt DNA sequence, or the capacity to form expected secondary structure. (TIF) [file pone.0038320.s007.tif]

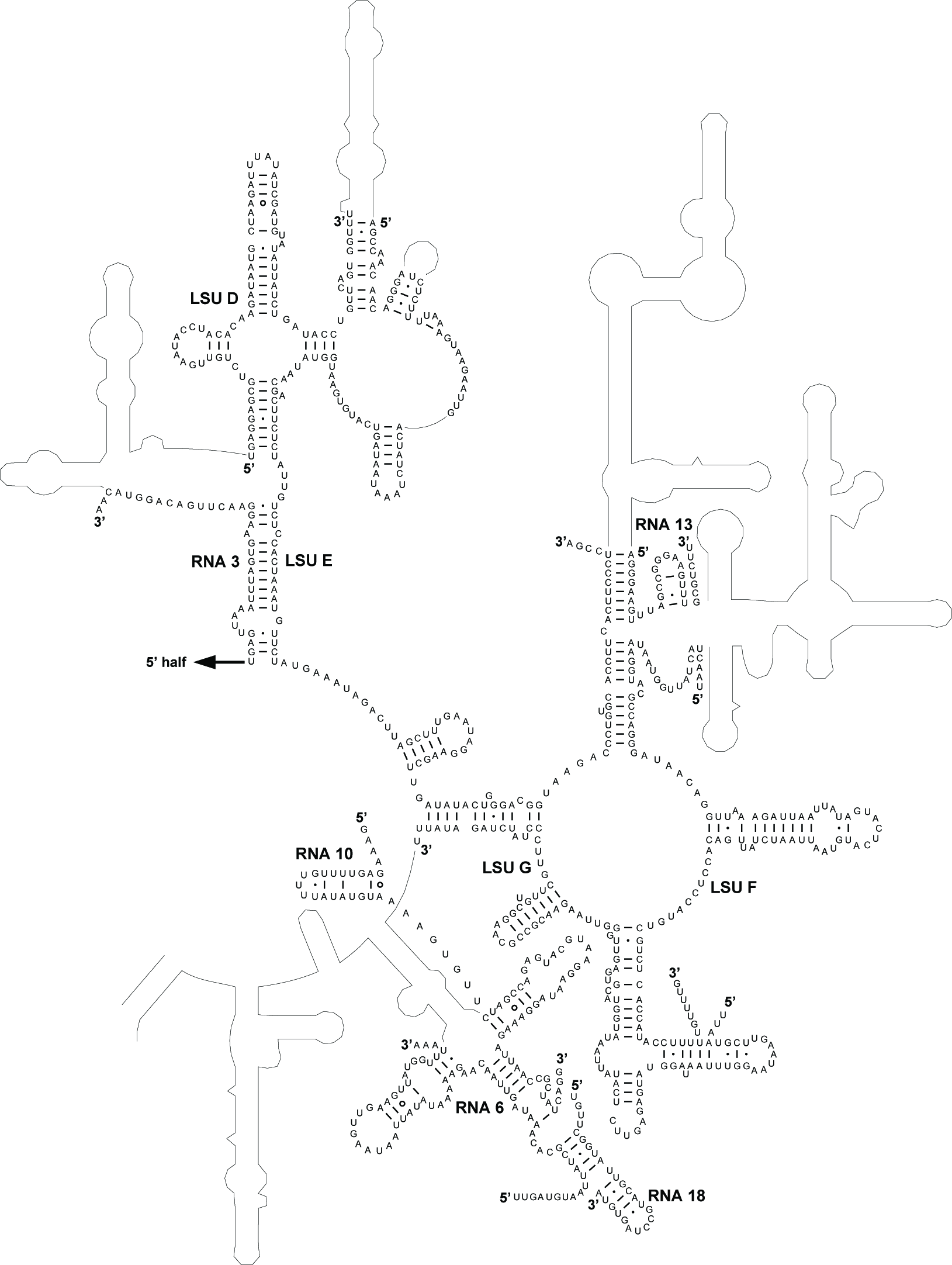

Supplement: Figure S8 — Proposed secondary structure for T. parva mt LSU rRNA (3′ half). The T. parva mt LSU rRNA (3′ half) secondary structure model is superimposed onto the E. coli LSU rRNA (3′ half) secondary structure model diagram. The transcript ends shown are artificial, based on the extent of sequence similarity between the mapped P. falciparum RNAs, and the T. parva mt DNA sequence, or the capacity to form expected secondary structure. (TIF) [file pone.0038320.s008.tif]

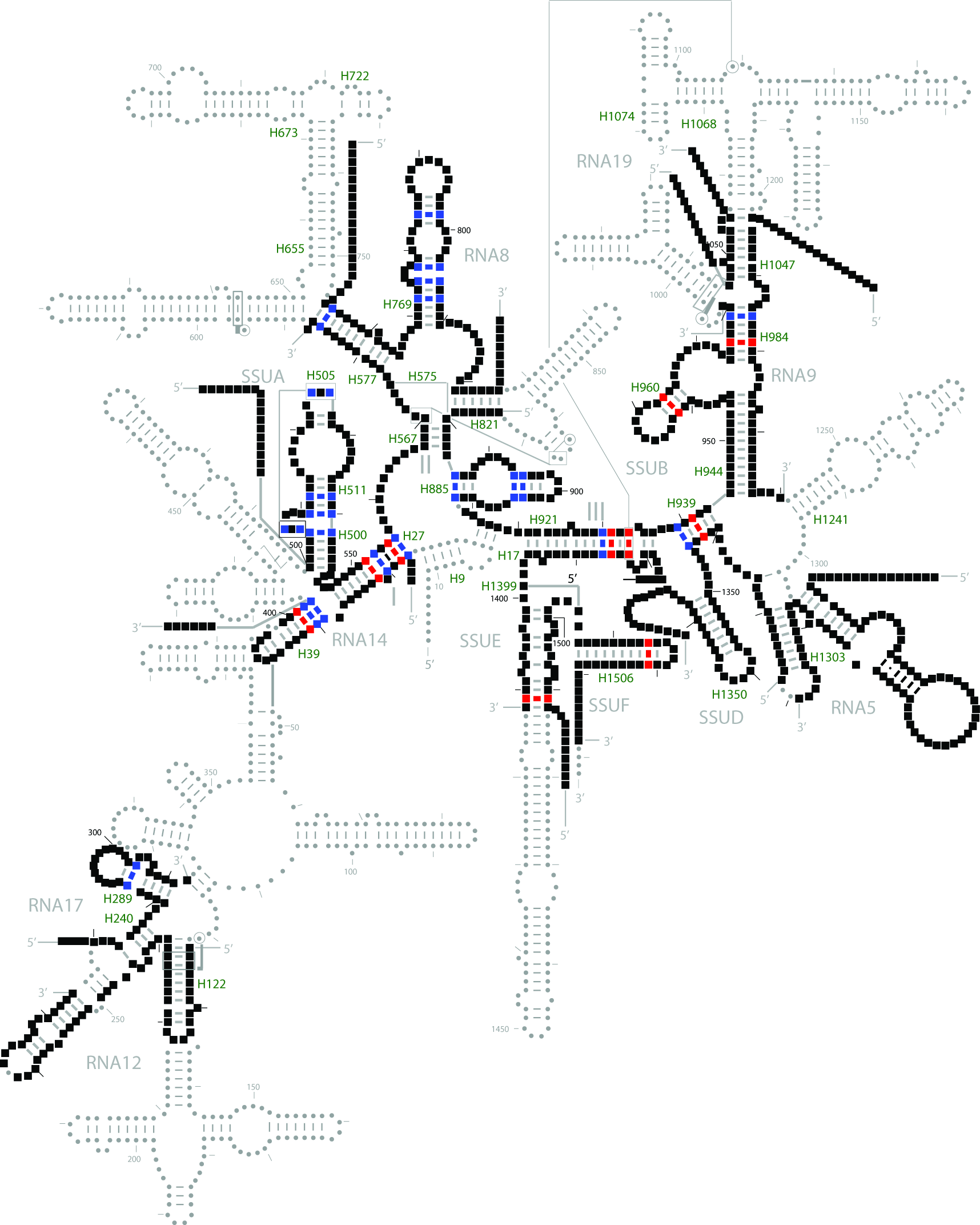

Supplement: Figure S9 — Location of type 1 and 2 covariations in SSU rRNA. The P. falciparum mt SSU rRNA secondary structure model, shown in thick gray lines, is superimposed onto the E. coli SSU rRNA secondary structure model diagram. Regions where P. falciparum has no structure equivalent to E. coli are shown using gray circles and lines. Category 1 covarying pairs are indicated with red and category 2 with blue. (TIF) [file pone.0038320.s009.tif]

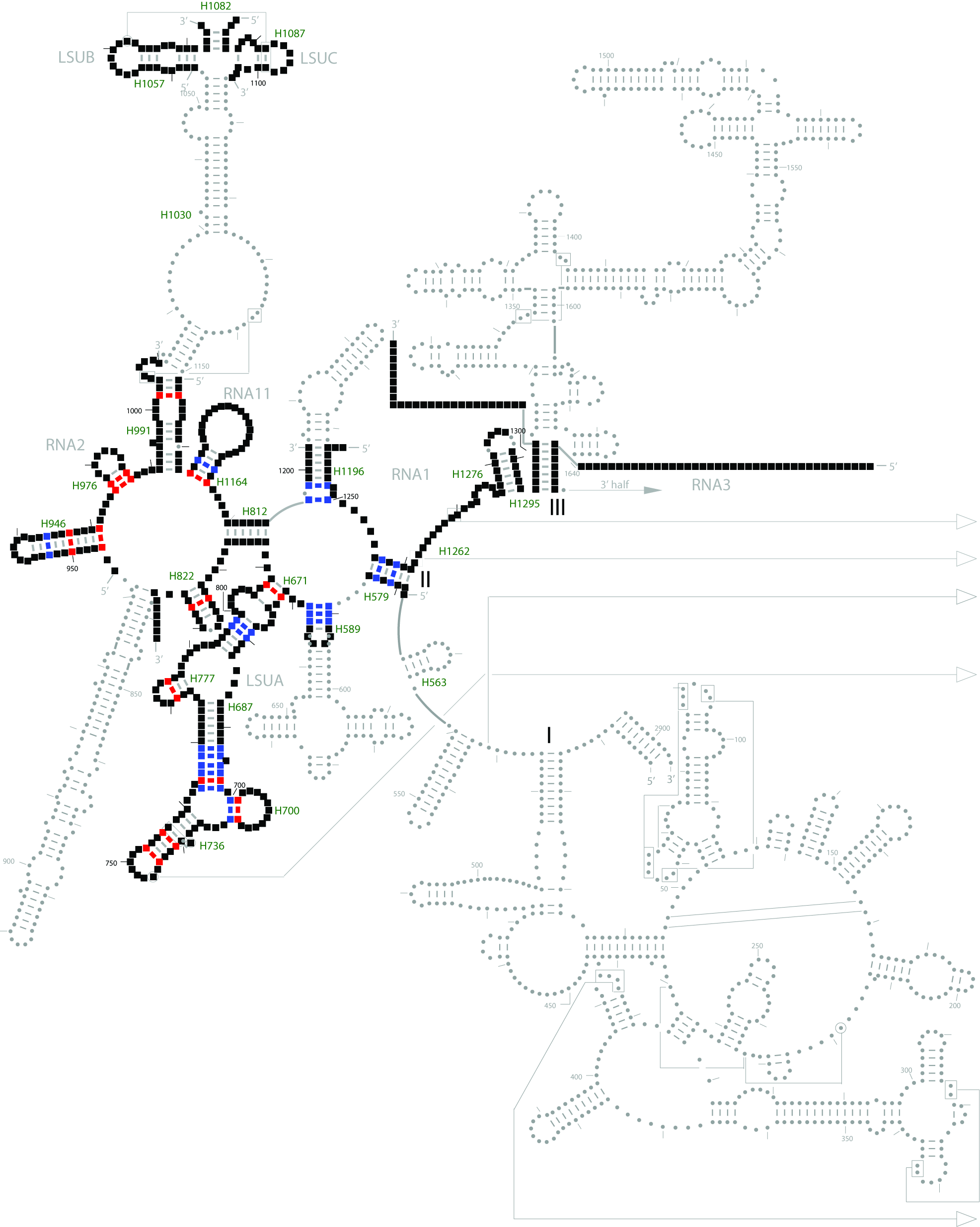

Supplement: Figure S10 — Location of type 1 and 2 covariations in LSU rRNA (5′ half). The P. falciparum mt LSU rRNA (5′ half) secondary structure model, shown in thick gray lines, is superimposed onto the E. coli LSU rRNA (5′ half) secondary structure model diagram. Regions where P. falciparum has no structure equivalent to E. coli are shown using gray circles and lines. Category 1 covarying pairs are indicated with red and category 2 with blue. (TIF) [file pone.0038320.s010.tif]

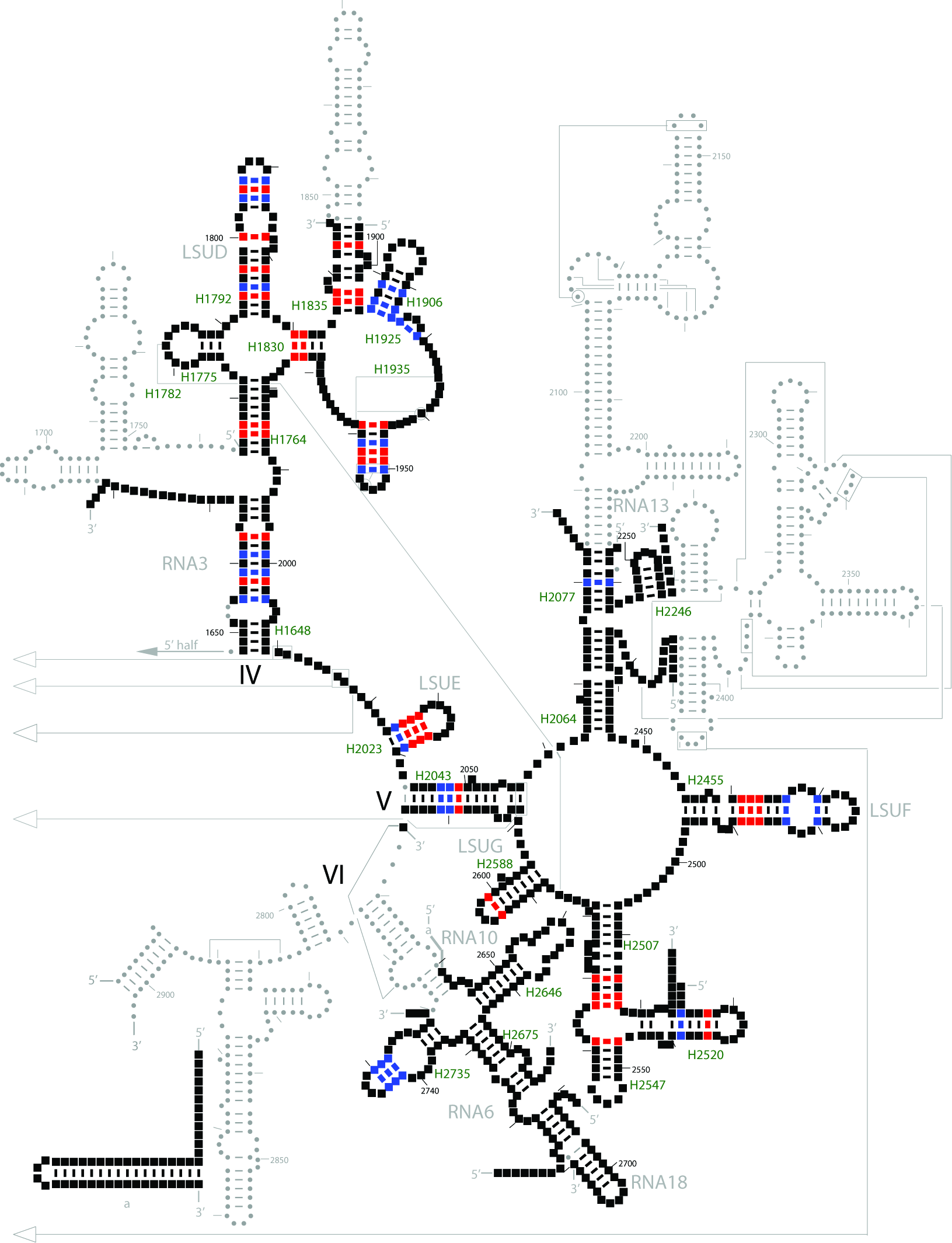

Supplement: Figure S11 — Location of type 1 and 2 covariations in LSU rRNA (3′ half). The P. falciparum mt LSU rRNA (3′ half) secondary structure model, shown in thick gray lines, is superimposed onto the E. coli LSU rRNA (3′ half) secondary structure model diagram. Regions where P. falciparum has no structure equivalent to E. coli are shown using gray circles and lines. Category 1 covarying pairs are indicated with red and category 2 with blue. (TIF) [file pone.0038320.s011.tif]

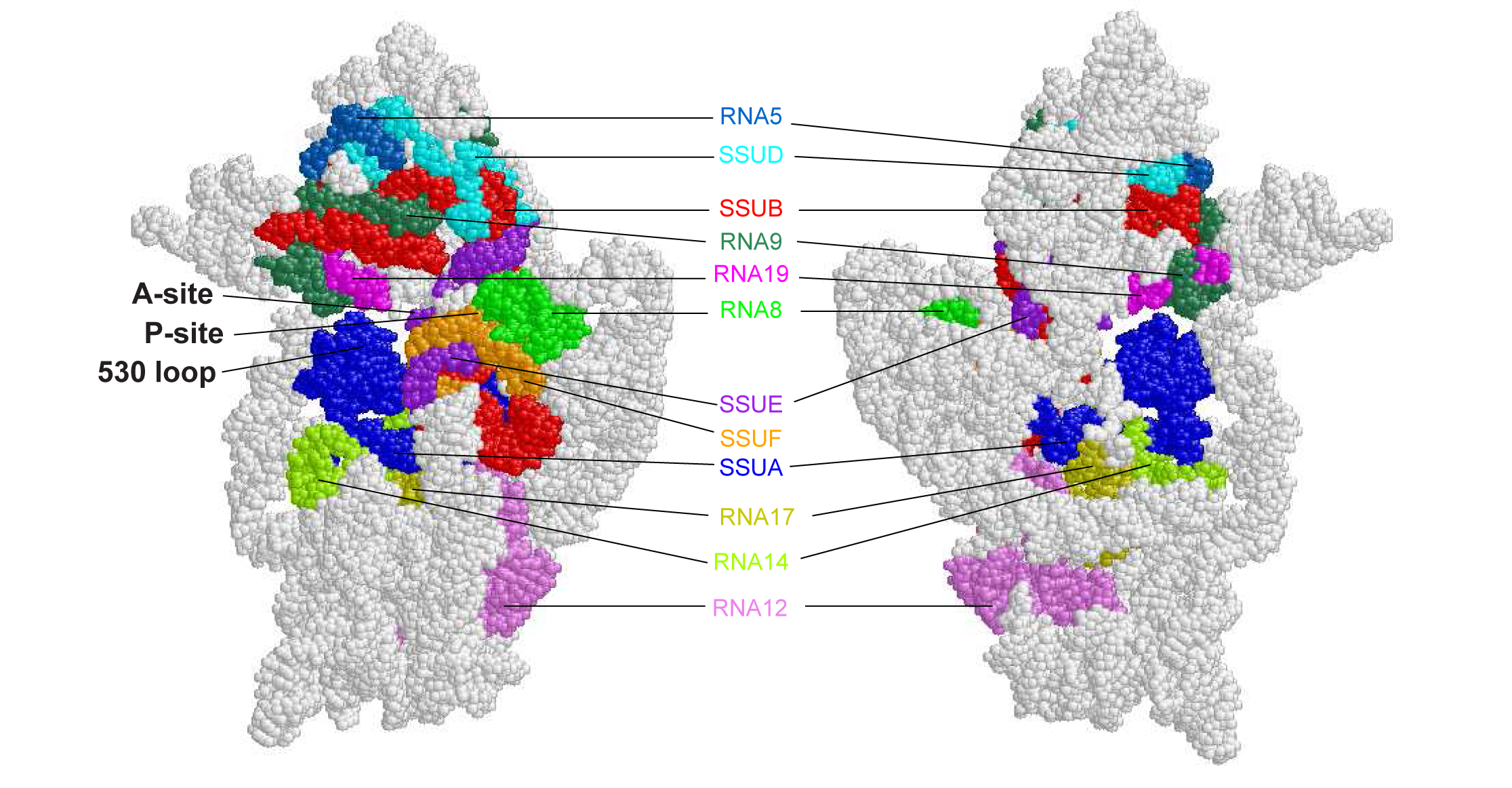

Supplement: Figure S12 — SSU rRNA tertiary structure. The P. falciparum mt SSU rRNAs are superimposed on a space-filling model of the three dimensional rRNA structure of Thermus thermophilus (PDB ID 1J5E; left = front/interface side, right = back). Each individual P. falciparum rRNA fragment is colored and labeled; regions of the model with no P. falciparum equivalent are colored gray. Functional regions of the rRNAs are labeled in black. (Full page version of Figure 5A.) (TIF) [file pone.0038320.s012.tif]

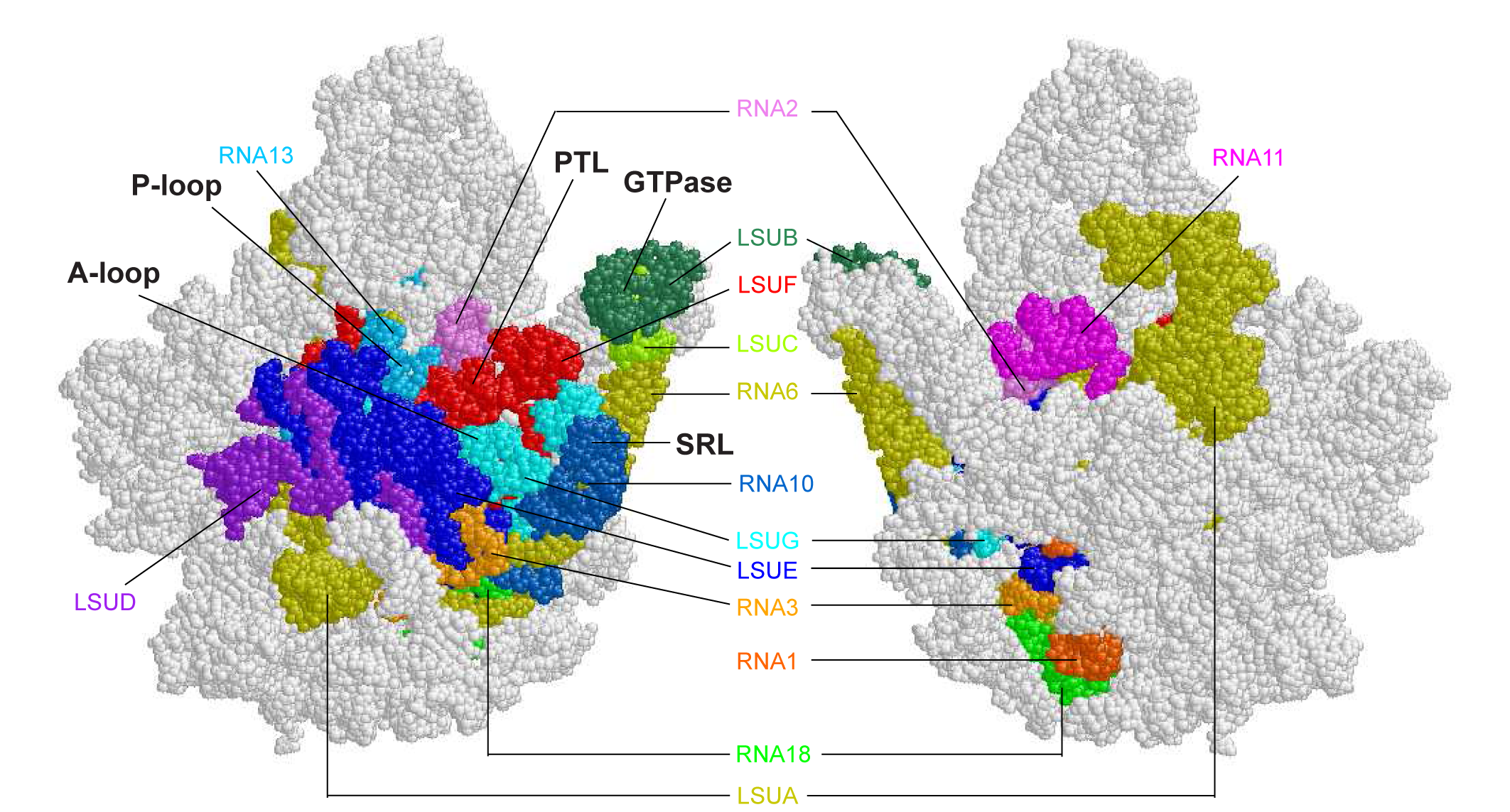

Supplement: Figure S13 — LSU rRNA tertiary structure. The P. falciparum LSU rRNA is superimposed on a space-filling model of the three dimensional rRNA structure of Haloarcula marismortui (PDB ID 1S72; left = crown/interface side, right = back). Each individual P. falciparum rRNA fragment is colored and labeled; regions of the model with no P. falciparum equivalent are colored gray. Functional regions of the rRNAs are labeled in black. (Full page version of Figure 5B.) (TIF) [file pone.0038320.s013.tif]

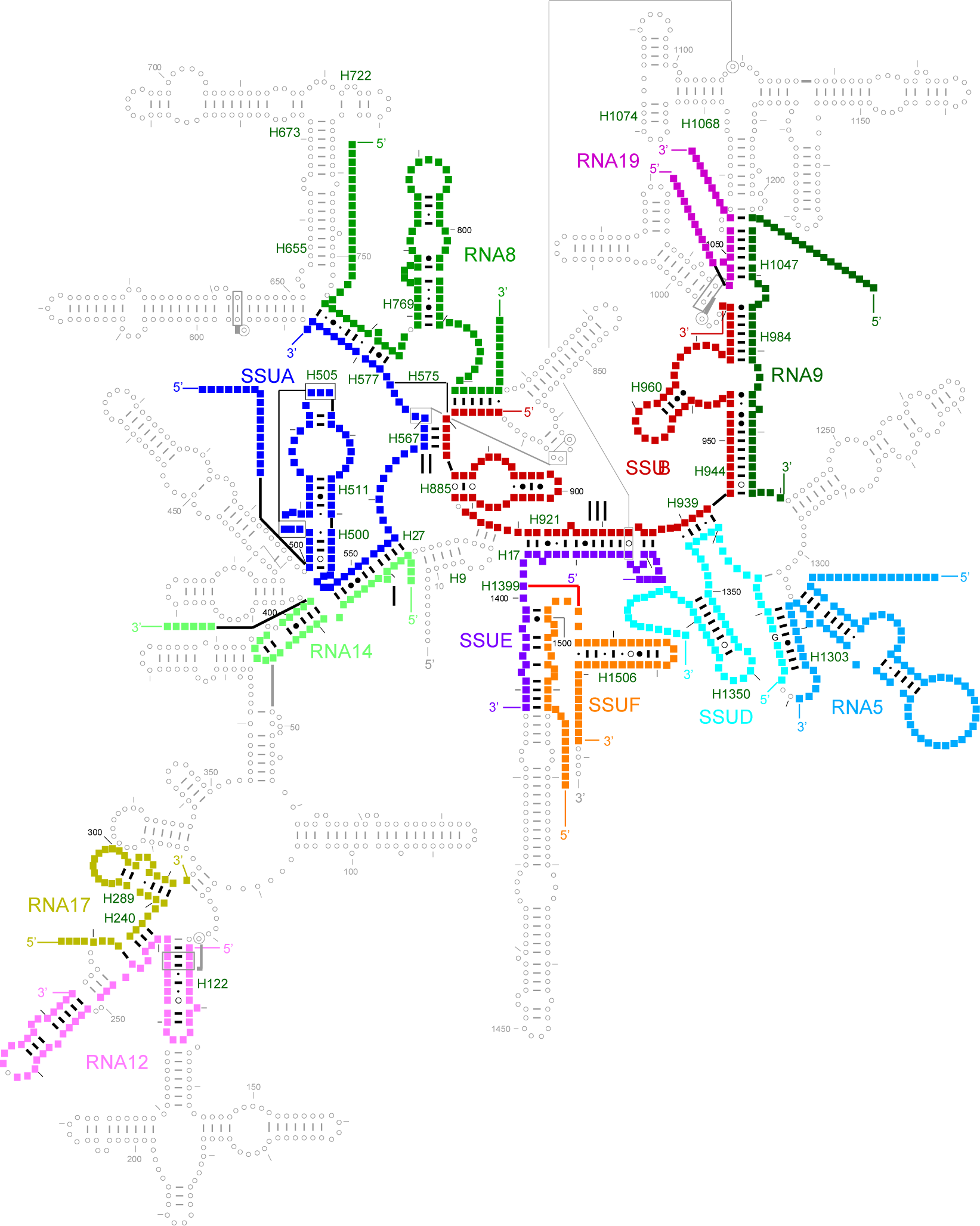

Supplement: Figure S14 — P. falciparum mt SSU rRNAs color-coded to tertiary structure. Secondary structure diagram for P. falciparum mt SSU rRNA with each P. falciparum mt rRNA fragment color-coordinated with the fragment colors in the Thermus thermophilus three-dimensional structure (Figure S12). (Full page version of Figure 5C.) (TIF) [file pone.0038320.s014.tif]

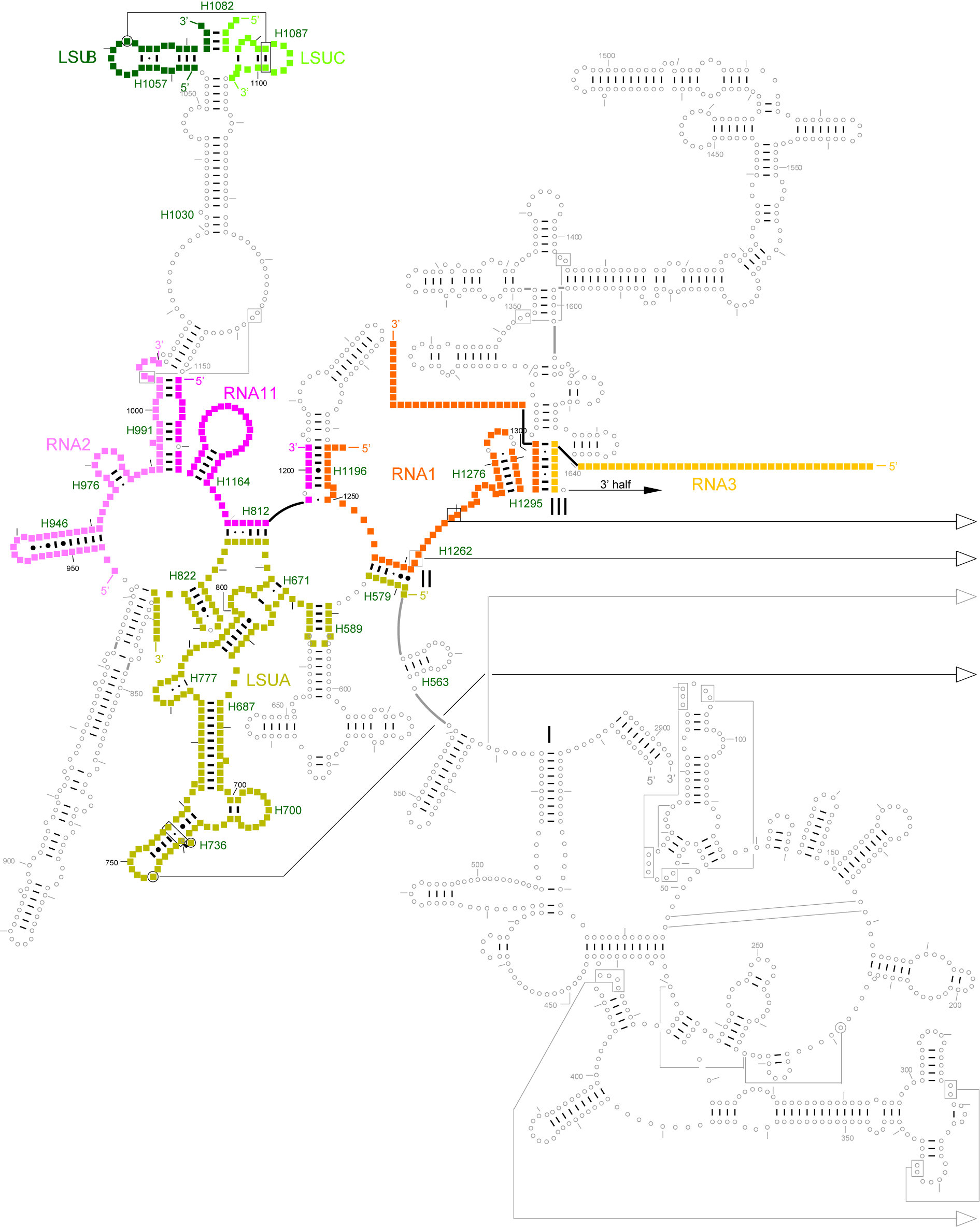

Supplement: Figure S15 — P. falciparum mt LSU rRNAs (5′ half) color-coded to tertiary structure. Secondary structure diagram for P. falciparum mt LSU rRNA (5′ half) with each P. falciparum mt rRNA fragment color-coordinated with the fragment colors in the Haloarcula marismortui three-dimensional structure (Figure S13). (Full page version of Figure 5D.) (TIF) [file pone.0038320.s015.tif]

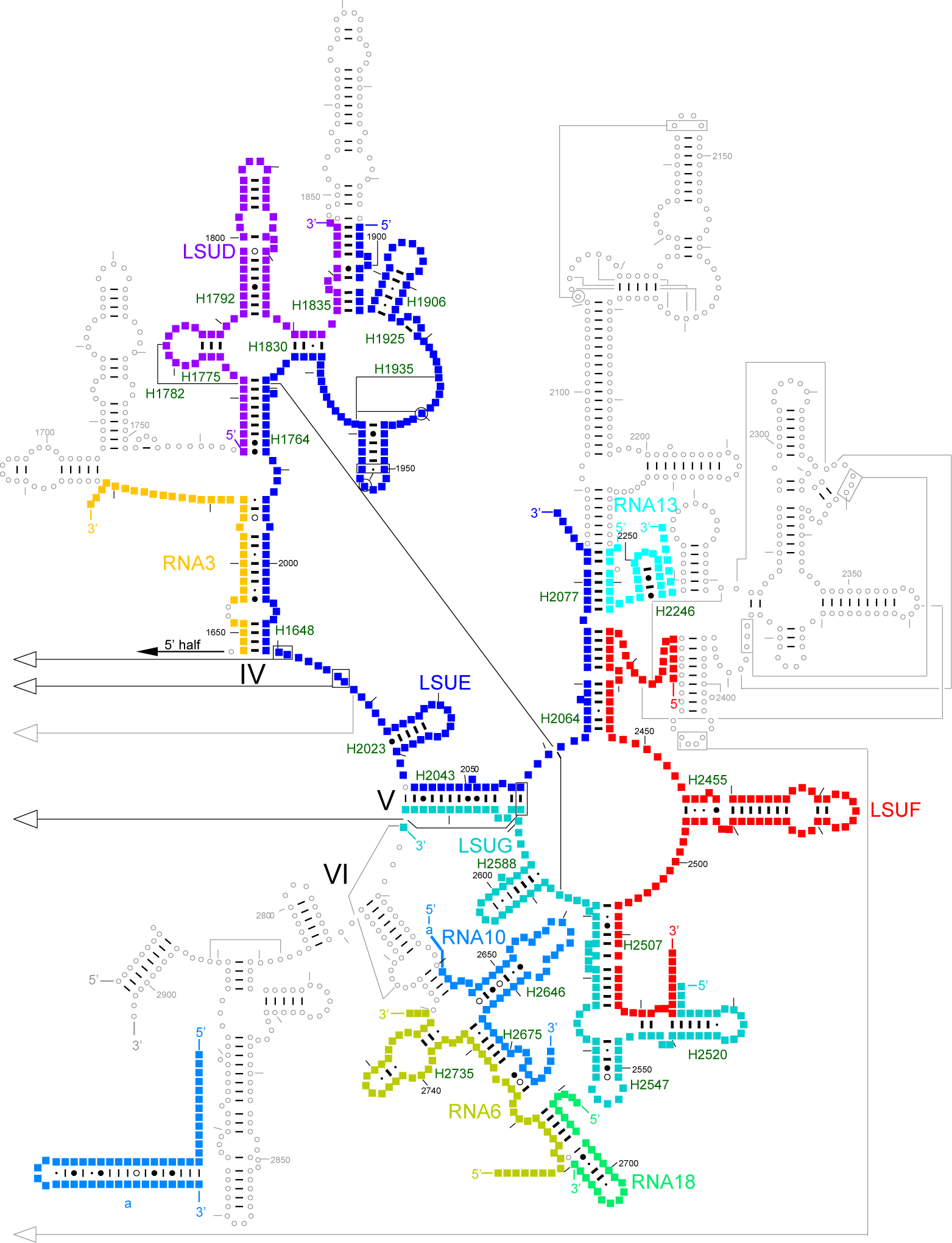

Supplement: Figure S16 — P. falciparum mt LSU rRNAs (3′ half) color-coded to tertiary structure. Secondary structure diagrams for P. falciparum mt LSU rRNA (3′ half) with each P. falciparum mt rRNA fragment color-coordinated with the fragment colors in the Haloarcula marismortui three-dimensional structure (Figure S13). (Full page version of Figure 5E.) (TIF) [file pone.0038320.s016.tif]
