# Supplementary material for: The Fragmented Mitochondrial Ribosomal RNAs of Plasmodium falciparum
Source: PLoS One. 2012 Jun 22;7(6):e38320. doi: 10.1371/journal.pone.0038320 (PMC3382252; doi:10.1371/journal.pone.0038320)
Supplement: Table S1 — Mt genomes from Plasmodium species and related apicomplexans. (PDF) [file pone.0038320.s017.pdf]

Table S1. Mt genomes from *Plasmodium* species and related apicomplexans.

Organism[1] (Host[2])	Mt DNA size (nt)[3]	identity to M76611	Initial Set[4]	Accession Data
***Plasmodium* (Haemosporida)**				
P. berghei (rodent)	5957	88.3%	Y	AB558173 (contig 5406, Sanger)
	5961	88.0%		AF014115
P. billbrayi (chimpanzee)	5945	91.8%		GQ355468
	5949	91.7%	Y	GQ355469
	5864	90.3%		GQ355470
	5865	90.5%		GQ355471
P. billcollinsi (chimpanzee)	5864	92.7%		GQ355477
	5864	92.8%		GQ355478
	5950	94.2%	Y	GQ355479
P. chabaudi (rodent)	5949	88.1%		AB379663
	5949	88.2%		AB379664
	5949	88.2%		AB379665
	5949	88.1%		AB379666
	5949	88.1%		AB379667
	5949	88.2%		AB379668
	5949	88.2%		AB379669
	5949	88.2%		AB379670
	5949	88.2%		AB379671
	5948	88.2%	Y	AF014116
P. coatneyi (OW monkey, Asian)	5976	88.5%	Y	AB354575
P. cynomolgi (OW monkey, Asian)	5983	88.8%		AB434919
	5982	88.8%		AB444121
	5985	88.8%		AB444122
	5986	88.9%		AB444123
	5983	88.8%		AB444124
	5983	88.8%		AB444125
	5993	88.9%		AB444126
	5986	88.9%		AB444127
	5986	88.9%		AB444128
	5991	88.8%		AB444129
	5984	88.8%		AB444130
	5984	88.8%		AB444131
	5884*	87.3%	Y	AY800108

Organism[1] (Host[2])	Mt DNA size (nt)[3]	identity to M76611	Initial Set[4]	Accession Data
P. falciparum (human)	5967	(self)	Y	M76611
	5967	99.9%		AJ276844 (NC_002375)
	5949	99.9%		AY282924
	5949	99.9%		AY282925
	5949	99.9%		AY282926
	5949	99.9%		AY282927
	5949	99.9%		AY282928
	5949	99.9%		AY282929
	5949	99.9%		AY282930
	5949	99.9%		AY282931
	5949	99.9%		AY282932
	5949	99.9%		AY282933
	5949	99.9%		AY282934
	5949	99.9%		AY282935
	5949	99.9%		AY282936
	5949	99.9%		AY282937
	5949	99.9%		AY282938
	5949	99.9%		AY282939
	5949	99.9%		AY282940
	5949	99.9%		AY282941
	5949	99.9%		AY282942
	5949	99.9%		AY282943
	5949	99.9%		AY282944
	5949	99.9%		AY282945
	5949	99.9%		AY282946.
	5949	99.9%		AY282947
	5949	99.9%		AY282948
	5949	99.9%		AY282949
	5949	99.9%		AY282950
	5949	100.0%		AY282951
	5949	100.0%		AY282952
	5949	99.9%		AY282953
	5949	99.9%		AY282954
	5949	99.9%		AY282955
	5949	99.9%		AY282956
	5949	99.9%		AY282957
	5949	99.9%		AY282958

Organism[1] (Host[2])	Mt DNA size (nt)[3]	identity to M76611	Initial Set[4]	Accession Data
	5949	99.9%		AY282959
	5949	100.0%		AY282960
	5949	99.9%		AY282961
	5949	99.9%		AY282962
	5949	99.9%		AY282963
	5949	99.9%		AY282964
	5949	99.9%		AY282965
	5949	99.9%		AY282966
	5949	99.9%		AY282967
	5949	99.9%		AY282968
	5949	99.9%		AY282969
	5949	100.0%		AY282970
	5949	100.0%		AY282971
	5949	99.9%		AY282972
	5953	99.9%		AY282973
	5949	99.9%		AY282974
	5949	99.9%		AY282975
	5949	99.9%		AY282976
	5949	99.9%		AY282977
	5949	99.9%		AY282978
	5949	99.9%		AY282979
	5949	99.9%		AY282980
	5949	99.9%		AY282981
	5949	99.9%		AY282982
	5949	100.0%		AY282983
	5949	99.9%		AY282984
	5949	100.0%		AY282985
	5949	99.9%		AY282986
	5949	100.0%		AY282987
	5949	100.0%		AY282988
	5949	99.9%		AY282989
	5949	99.9%		AY282990
	5949	99.9%		AY282991
	5949	99.9%		AY282992
	5949	99.9%		AY282993
	5949	99.9%		AY282994
	5949	99.9%		AY282995

Organism[1] (Host[2])	Mt DNA size (nt)[3]	identity to M76611	Initial Set[4]	Accession Data
	5949	99.9%		AY282996
	5949	99.9%		AY282997
	5949	99.9%		AY282998
	5949	99.9%		AY282999
	5949	99.9%		AY283000
	5949	99.9%		AY283001
	5949	99.9%		AY283002
	5949	99.9%		AY283003
	5949	99.9%		AY283004
	5949	99.9%		AY283005
	5949	99.9%		AY283006
	5949	99.9%		AY283007
	5949	99.9%		AY283008
	5949	99.9%		AY283009
	5949	99.9%		AY283010
	5949	99.9%		AY283011
	5949	99.9%		AY283012
	5949	100.0%		AY283013
	5949	100.0%		AY283014
	5949	100.0%		AY283015
	5949	99.9%		AY283016
	5949	99.9%		AY283017
	5949	100.0%		AY283018
	5967	99.8%		DQ642845
P. fieldi (OW monkey, Asian)	5983	89.0%	Y	AB354574
	5988	89.0%		AB444132
	5987	89.0%		AB444133
P. floridense (lizard)	6002	88.3%	Y	EF079654 (NC_009961)
P. fragile (OW monkey, Asian)	5977	88.7%		AB444134
	5978	88.8%		AB444135
	5977	88.7%		AB444136
	5977	88.7%	Y	AY722799 (NC_012369)
P. gallinaceum (bird)	6003	88.3%	Y	AB250690 (NC_008288)
	6002	88.1%		AB599930
P. gonderi (OW monkey, African)	5989	89.1%		AB434918
	5900*	87.7%	Y	AY800111
P. hylobati (OW monkey, Asian)	5973	88.3%	Y	AB354573

Organism[1] (Host[2])	Mt DNA size (nt)[3]	identity to M76611	Initial Set[4]	Accession Data
P. inui (OW monkey, Asian)	5972	88.0%		AB354572
	5976	88.5%		AB444109
	5981	88.4%		AB444110
	5976	88.2%		AB444111
	5980	88.4%		AB444112
	5976	88.2%		AB444113
	5981	88.2%		AB444114
	5971	88.0%		AB444115
	5973	87.9%		AB444116
	5976	88.2%		AB444117
	5972	87.9%		AB444118
	5979	88.4%		AB444119
	5976	88.5%		AB444120
	5972	87.9%	Y	HM032052
P. juxtanucleare (bird)	6014	87.8%	Y	AB250415
P. knowlesi (OW monkey, Asian; human[5])	5957	88.4%		AB444106
	5958	88.4%		AB444107
	5958	88.3%		AB444108
	5957	88.4%	Y	AY722797 (NC_007232)
P. malariae (human)	5968	88.8%	Y	AB354570
	5969	88.7%		AB489192
	5969	88.7%		AB489193
	5968	88.7%		AB489194
	5856	87.0%		GQ355485
	5856	87.0%		GQ355486
P. mexicanum (lizard)	5991	87.9%		AB375765
	5991	87.9%	Y	EF079653 (NC_009960)
P. ovale (human)	5974	89.1%	Y	AB354571
P. reichenowi (chimpanzee)	5966	97.4%	Y	AJ251941 (NC_002235)
P. relictum (bird)	5996	88.4%		AY733088 (NC_012426)
	6003	87.8%		AY733089
	5997	88.8%	Y	AY733090
P. simiovale (OW monkey, Asian)	5987	89.0%		AB434920
	5898*	87.6%	Y	AY800109
P. simium (NW monkey)	5990	88.9%	Y	AY722798 (NC_007233)
	5899	87.5%		AY800110
P. vinckei vinckei (rodent)	5948	87.5%		AB599931

Organism[1] (Host[2])	Mt DNA size (nt)[3]	identity to M76611	Initial Set[4]	Accession Data
P. sp. DAJ-2004 (OW monkey, African)	5896*	87.3%	Y	AY800112
P. vivax (human)	5991	88.9%		AY598035
	5990	88.9%		AY598036
	5991	88.9%		AY598037
	5991	88.9%		AY598038
	5991	88.9%		AY598039
	5991	88.9%		AY598040
	5994	88.9%		AY598041
	5989	88.9%		AY598042
	5990	88.9%		AY598043
	5991	88.9%		AY598044
	5991	88.9%		AY598045
	5989	88.9%		AY598046
	5989	88.9%		AY598047
	5992	88.9%		AY598048
	5991	88.9%		AY598049
	5989	88.9%		AY598050
	5990	88.9%	Y	AY598140 (NC_007243)
P. yoelii (rodent)	5956	88.4%	Y	M29000 M21313 M33978 (MALPY00209 (TIGR))
**Other Haemosporida**				
H. sp. jb1.JA27 (bird)	5970	86.6%		AY733086 (NC_012423)
H. sp. jb2.SEW5141 (bird)	5970	86.8%	Y	AY733087 (NC_012425)
H. columbae (bird)	5988	83.3%	Y	FJ168562 (NC_012448)
Hepatocystis sp. (bat)	6259	86.3%	Y	FJ168565
L. caulleryi (bird)	5959	85.9%	Y	AB302215
L. fringillarium (bird)	5992	83.2%	Y	FJ168564 (NC_012451)
L. majoris (bird)	6684	83.5%	Y	FJ168563 (NC_012450)
L. sabrasezi (bird)	5935	82.6%	Y	AB299369 (NC_009336)
Parahaemoproteus vireonis (bird)	5893*	86.6%	Y	FJ168561 (NC_012447)
***Eimeria* (Coccidia)**				
Eimeria tenella (bird)	6213	NA		AB564272
***Babesia* and *Theileria* (Piroplasmida)**				
B. bovis (cow)	5970	NA		AB499088
	6005	NA		EU075182
B. caballi (horse)	5847	NA		AB499086
B. gibsoni (dog)	5865	NA		AB499087
T. annulata (cow)	5905	NA		CR940346 (NT_167255)

Organism[1] (Host[2])	Mt DNA size (nt)[3]	identity to M76611	Initial Set[4]	Accession Data
T. equi (horse)	8246	NA		AB499091
T. orientalis (cow)	5957	NA		AB499090
T. parva (cow)	5924	NA		AB499089
	5895	NA		Z23263 (011005)

[1] *P.*, *Plasmodium*; *H.*, *Haemoproteus*; *L.*, *Leucocytozoon*; *B.*, *Babesia*; *T.*, *Theileria*.

[2] OW, Old World; NW, New World.

[3] * mt genome sequence incomplete; presumed to reflect PCR primer choice.

[4] Complete (or nearly complete) mitochondrial genomes of 25 *Plasmodium* species, one *Plasmodium* isolate, and eight related hemosporidian species used for initial alignment.

[5] *P. knowlesi*, previously considered only a monkey parasite, has recently been found to cause human infections as well [86].
