# Supplementary material for: The Fragmented Mitochondrial Ribosomal RNAs of Plasmodium falciparum
Source: PLoS One. 2012 Jun 22;7(6):e38320. doi: 10.1371/journal.pone.0038320 (PMC3382252; doi:10.1371/journal.pone.0038320)
Supplement: Table S2 — Intergenic size variation among hemosporidian mt genomes. (PDF) [file pone.0038320.s018.pdf]

Table S2. Intergenic size variation among hemosporidian mt genomes.

Organism[a]	RNA23t> <LSUC[b]	RNA10< <cox3[c]	cox3< >LSUF	LSUF> >SSUE	SSUE> >RNA2	RNA3> <SSUA	SSUA< >cox1	cox1[c]> >cob[c]	RNA4> >RNA5	RNA5> >RNA6	RNA12> >RNA18	RNA8< >RNA16
P. falciparum	0	9	28	7	16	5	13	20	19	5	18	15
P. reichenowi	0	9	28	7	16	5	13	20	19	5	18	15
P. billcollinsi	1	9	34	7	16	5	13	20	19	5	18	15
P. billbrayi	0	9	38	7	15	5	13	20	19	4	18	14
P. gallinaceum	1	10	32	10	17	5	17	14	19	7	25	15
P. relictum	0	10	32	10	14	5	17	14	19	7	25	15
P. juxtanucleare	7	11	31	15	18	5	18	14	18	7	24	15
P. floridense	0	10	31	9	17	5	17	15	19	0	25	18
P. mexicanum	1	10	33	12	16	5	13	14	19	0	18	18
P. ovale	1	9	30	11	18	5	17	7	19	4	20	9
P. malariae	1	9	31	7	18	5	19	14	19	4	20	9
P. gonderi	0	8	30	11	18	5	28	27	19	1	19	16
***P.* sp. DAJ-2004**	2	6	30	11	17	5	29	24	19	4	19	15
P. fragile	2	6	30	11	18	5	26	21	20	4	20	10
P. knowlesi	2	6	30	11	18	5	14	18	19	4	20	6
P. coatneyi	2	6	30	11	18	5	32	21	18	3	20	6
P. inui	2	6	30	11	17	5	17	20	29	4	20	11
P. hylobati	2	6	31	11	17	5	24	21	29	4	20	9
P. vivax	2	12	30	11	18	5	24	21	19	4	20	11
P. simium	2	12	30	11	18	5	31	21	19	4	20	11
P. simiovale	2	8	30	11	18	5	28	17	19	4	20	11
P. fieldi	2	8	32	11	18	5	25	13	19	4	20	11
P. cynomolgi	2	10	30	11	18	5	23	15	19	4	20	15
P. yoelii	1	6	30	11	16	5	17	19	19	0	22	4
P. berghei	1	6	30	11	16	5	17	19	19	0	22	4

Organism[a]	RNA23t> <LSUC[b]	RNA10< <cox3[c]	cox3< >LSUF	LSUF> >SSUE	SSUE> >RNA2	RNA3> <SSUA	SSUA< >cox1	cox1[c]> >cob[c]	RNA4> >RNA5	RNA5> >RNA6	RNA12> >RNA18	RNA8< >RNA16
P. chabaudi	1	6	30	11	15	5	17	17	19	0	21	4
***Haemoproteus* sp.**	4	11	32	7	17	5	13	15	0	0	25	16
**Parahaemoproteus**	4	12	32	7	17	5	17	15	12	0	25	NA
L. caulleryi	2	4	32	4	17	5	14	16	11	0	25	13
L. sabrasezi	2	10	32	4	4	2	13	13	11	0	27	17
L. majoris	4	11	32	6	17	3	17	15	11	4	27	14
L. fringillarium	4	11	33	6	17	9	17	15	11	10	27	14
H. columbae	3	10	31	7	17	5	18	19	8	0	24	17
***Hepatocystis* sp.**	20	19	62	16	18	18	46	94	24	0	0	28

[a] Organisms arranged by relatedness of mt genomes from Clustal W alignment, to assist pattern identification. *P*, *Plasmodium*; *L*, *Leucocytozoon*; *H*, *Haemoproteus*.

[b] Gene pairs flanking a gap of 10 nt or more are shown with carets indicating the direction of transcription. The number of nt in the gap is given, based on the 5' or 3' end of the flanking transcripts, as appropriate.

[c] For these genes, the gap is calculated from the end of the ORF, not the transcript end. This was done because using the transcript end would have required negative numbers for some gaps.
