# Supplementary material for: The Fragmented Mitochondrial Ribosomal RNAs of Plasmodium falciparum
Source: PLoS One. 2012 Jun 22;7(6):e38320. doi: 10.1371/journal.pone.0038320 (PMC3382252; doi:10.1371/journal.pone.0038320)
Supplement: Table S3 — Similarity of individual mt genes from Plasmodium species relative to P. falciparum. (PDF) [file pone.0038320.s019.pdf]

Table S3. Similarity of individual mt genes from *Plasmodium* species relative to *P. falciparum*.

gene	rRNA order[a]	size (nt)	*berghei*	*billbrayi*	*billcollinsi*	*chabaudi*	*coatneyi*	*cynomolgi*	*fieldi*	*floridense*	*fragile*	*gallinaceum*	*gonderi*	*hylobati*	*inui*	*juxtanucleare*	*knowlesi*	*malariae*	mandrill[b]	*mexicanum*	*ovale*	*reichenowi*	*relictum*	*simiovale*	*simium*	*vivax*	*yoeli*	average
RNA14	S1	39	100.0%	97.4%	100.0%	100.0%	100.0%	100.0%	100.0%	100.0%	100.0%	100.0%	100.0%	100.0%	92.3%	100.0%	97.4%	97.4%	100.0%	100.0%	100.0%	100.0%	100.0%	100.0%	100.0%	100.0%	100.0%	99.5%
RNA12	S2	59	89.8%	98.5%	100.0%	86.4%	93.2%	91.5%	93.2%	93.2%	91.5%	96.6%	96.6%	93.2%	89.8%	94.9%	89.8%	91.5%	91.5%	93.2%	93.2%	100.0%	94.9%	91.5%	89.8%	89.8%	89.8%	92.7%
RNA17	S3	40	97.5%	97.6%	97.5%	97.5%	97.5%	100.0%	100.0%	100.0%	95.0%	100.0%	100.0%	100.0%	100.0%	100.0%	100.0%	95.0%	100.0%	100.0%	100.0%	97.5%	100.0%	100.0%	100.0%	100.0%	95.0%	98.9%
SSUA	S4	108	92.6%	99.1%	99.1%	92.6%	93.5%	93.5%	94.4%	94.4%	94.4%	93.5%	93.5%	94.4%	93.5%	93.5%	93.5%	93.5%	93.5%	94.4%	93.5%	100.0%	93.5%	93.5%	93.5%	93.5%	92.6%	94.3%
RNA8	S5	100	94.0%	99.1%	99.0%	95.0%	96.0%	95.0%	96.0%	91.0%	95.0%	94.0%	97.0%	96.0%	95.0%	94.0%	95.0%	96.0%	96.0%	95.0%	95.0%	99.0%	93.0%	94.0%	95.0%	95.0%	93.0%	95.3%
SSUB	S6	116	97.4%	99.2%	99.1%	97.4%	99.1%	97.4%	99.1%	96.6%	96.6%	97.4%	97.4%	99.1%	97.4%	97.4%	97.4%	97.4%	97.4%	97.4%	99.1%	99.1%	97.4%	97.4%	97.4%	97.4%	97.4%	97.8%
RNA19	S7	30	100.0%	96.7%	100.0%	100.0%	100.0%	100.0%	100.0%	100.0%	100.0%	100.0%	100.0%	100.0%	100.0%	100.0%	100.0%	100.0%	100.0%	100.0%	100.0%	100.0%	100.0%	100.0%	100.0%	100.0%	100.0%	100.0%
RNA9	S8	54	94.4%	98.3%	98.1%	94.4%	92.6%	92.6%	94.4%	92.6%	92.6%	94.4%	94.4%	94.4%	90.7%	94.4%	92.6%	96.3%	94.4%	100.0%	94.4%	98.1%	96.3%	96.3%	92.6%	90.7%	98.1%	94.7%
RNA5	S9	87	89.7%	99.0%	95.4%	87.4%	90.8%	88.5%	89.7%	87.4%	88.5%	86.2%	87.4%	88.5%	87.4%	86.2%	89.7%	88.5%	89.7%	85.1%	87.4%	98.9%	87.4%	88.5%	87.4%	89.7%	90.8%	89.2%
SSUD	S10	68	98.5%	98.6%	100.0%	98.5%	95.6%	95.6%	95.6%	95.6%	95.6%	92.6%	95.6%	97.1%	94.1%	94.1%	95.6%	97.1%	95.6%	94.1%	97.1%	100.0%	94.1%	95.6%	95.6%	95.6%	97.1%	96.1%
SSUE	S11	42	100.0%	97.6%	100.0%	100.0%	100.0%	100.0%	100.0%	97.6%	100.0%	100.0%	100.0%	100.0%	100.0%	100.0%	100.0%	100.0%	100.0%	97.6%	100.0%	100.0%	100.0%	100.0%	100.0%	100.0%	100.0%	99.8%
SSUF	S12	61	98.4%	98.4%	100.0%	98.4%	100.0%	100.0%	100.0%	100.0%	100.0%	98.4%	100.0%	100.0%	100.0%	100.0%	100.0%	100.0%	100.0%	100.0%	100.0%	98.4%	100.0%	100.0%	100.0%	100.0%	98.4%	99.5%
**average SSU rRNA**		**804**	**95.3%**	**97.8%**	**94.9%**	**96.0%**	**98.9%**	**95.4%**	**96.3%**	**94.8%**	**95.1%**	**96.3%**	**95.1%**	**96.0%**	**94.5%**	**95.3%**	**95.3%**	**95.5%**	**95.8%**	**95.5%**	**95.9%**	**99.3%**	**95.4%**	**95.5%**	**95.1%**	**95.3%**	**95.3%**	**95.8%**
LSUA	L1	176	97.7%	99.4%	99.3%	97.2%	96.6%	85.2%	97.7%	98.9%	97.7%	97.7%	85.2%	98.3%	97.2%	98.9%	97.2%	98.3%	99.3%	97.7%	98.3%	97.7%	98.9%	85.2%	97.2%	97.2%	97.7%	98.0%
RNA2	L2	67	92.5%	98.6%	98.5%	94.0%	94.0%	92.5%	95.5%	92.5%	94.0%	94.0%	92.5%	95.5%	92.5%	92.5%	91.0%	95.5%	92.5%	92.5%	95.5%	100.0%	92.5%	94.0%	94.0%	92.5%	92.5%	94.1%
LSUB	L3	29	100.0%	96.6%	100.0%	100.0%	100.0%	100.0%	100.0%	100.0%	100.0%	100.0%	100.0%	96.6%	96.6%	93.1%	100.0%	96.6%	100.0%	96.6%	100.0%	100.0%	100.0%	100.0%	100.0%	100.0%	100.0%	99.2%
LSUC	L4	22	95.5%	95.7%	90.9%	95.5%	95.5%	95.5%	95.5%	86.4%	86.4%	90.9%	95.5%	95.5%	100.0%	90.9%	90.9%	100.0%	100.0%	90.9%	90.9%	100.0%	90.9%	95.5%	95.5%	95.5%	95.5%	94.2%
RNA11	L5	57	91.2%	98.4%	98.2%	94.7%	89.5%	91.2%	93.0%	91.2%	91.2%	93.0%	93.0%	91.2%	91.2%	100.0%	89.5%	91.2%	89.5%	94.7%	93.0%	98.2%	91.2%	87.7%	87.7%	91.2%	91.2%	92.5%
RNA1	L6	88	96.6%	98.9%	98.9%	96.6%	98.9%	96.6%	97.7%	93.2%	96.6%	95.5%	96.6%	98.9%	95.5%	98.9%	97.7%	95.5%	94.3%	95.5%	96.6%	100.0%	98.9%	96.6%	96.6%	96.6%	95.5%	96.9%
RNA3	L7	81	95.1%	98.8%	100.0%	93.8%	95.1%	96.3%	96.3%	93.8%	93.8%	97.5%	96.3%	95.1%	95.1%	97.5%	96.3%	96.3%	95.1%	95.1%	96.3%	100.0%	97.5%	96.3%	96.3%	96.3%	95.1%	96.1%
LSUD	L8	83	97.6%	98.8%	100.0%	97.6%	97.6%	97.6%	97.6%	97.6%	97.6%	97.6%	98.8%	97.6%	97.6%	97.6%	98.8%	97.6%	98.8%	97.6%	97.6%	100.0%	97.6%	97.6%	97.6%	97.6%	97.6%	98.0%
LSUE	L9	195	98.5%	99.5%	100.0%	99.5%	98.5%	98.5%	99.0%	97.4%	99.0%	97.9%	99.0%	99.0%	98.5%	97.9%	99.0%	98.5%	99.0%	96.9%	99.0%	100.0%	97.4%	98.5%	98.5%	99.0%	99.0%	98.7%
RNA13	L10	30	96.7%	96.8%	100.0%	96.7%	100.0%	100.0%	100.0%	96.7%	96.7%	96.7%	96.7%	100.0%	100.0%	100.0%	96.7%	100.0%	96.7%	96.7%	100.0%	100.0%	96.7%	96.7%	100.0%	96.7%	96.7%	98.3%
LSUF	L11	115	98.3%	99.1%	100.0%	97.4%	99.1%	98.3%	99.1%	93.9%	98.3%	96.5%	99.1%	98.3%	98.3%	97.4%	98.3%	97.4%	100.0%	97.4%	98.3%	100.0%	97.4%	98.3%	98.3%	98.3%	98.3%	98.2%
LSUG	L12	107	98.1%	99.1%	99.1%	97.2%	100.0%	100.0%	100.0%	100.0%	100.0%	100.0%	100.0%	100.0%	100.0%	100.0%	100.0%	100.0%	100.0%	100.0%	100.0%	100.0%	100.0%	100.0%	100.0%	100.0%	98.1%	99.7%
RNA10	L13	100	82.0%	99.2%	100.0%	79.0%	87.0%	83.0%	90.0%	85.0%	78.0%	83.0%	82.0%	91.0%	88.0%	82.0%	78.0%	85.0%	88.0%	86.0%	85.0%	98.0%	83.0%	83.0%	83.0%	83.0%	82.0%	89.1%
RNA18	L14	25	100.0%	96.0%	100.0%	100.0%	100.0%	100.0%	100.0%	100.0%	100.0%	100.0%	100.0%	100.0%	100.0%	100.0%	100.0%	100.0%	100.0%	100.0%	100.0%	100.0%	100.0%	100.0%	100.0%	100.0%	100.0%	100.0%
RNA6	L15	58	96.6%	98.3%	100.0%	96.6%	96.6%	96.6%	98.3%	100.0%	98.3%	98.3%	98.3%	98.3%	98.3%	96.6%	98.3%	98.3%	98.3%	98.3%	98.3%	100.0%	100.0%	98.3%	96.6%	96.6%	96.6%	98.1%
**average LSU rRNA**		**1233**	**96.3%**	**99.2%**	**96.2%**	**96.5%**	**99.4%**	**96.8%**	**97.3%**	**95.5%**	**96.4%**	**97.2%**	**96.4%**	**97.1%**	**96.4%**	**97.1%**	**96.3%**	**96.5%**	**96.9%**	**95.9%**	**96.8%**	**99.5%**	**96.6%**	**96.8%**	**96.6%**	**96.7%**	**96.3%**	**96.9%**
**average mt rRNAs**		**2037**	**95.9%**	**98.6%**	**95.7%**	**96.3%**	**99.2%**	**96.2%**	**96.9%**	**95.2%**	**95.9%**	**96.9%**	**95.9%**	**96.7%**	**95.7%**	**96.4%**	**95.9%**	**96.1%**	**96.5%**	**95.8%**	**96.4%**	**99.4%**	**96.1%**	**96.3%**	**96.0%**	**96.1%**	**95.9%**	**96.5%**
RNA4	unassigned	72	86.1%	96.9%	98.6%	83.3%	87.5%	86.1%	86.1%	90.3%	84.7%	90.3%	86.1%	86.1%	84.7%	88.9%	76.4%	84.7%	86.1%	88.9%	86.1%	100.0%	90.3%	87.5%	84.7%	84.7%	86.1%	87.7%
RNA7	unassigned	82	97.6%	97.6%	100.0%	96.3%	96.3%	ND[c]	97.6%	96.3%	97.6%	96.3%	ND[c]	97.6%	96.3%	96.3%	96.3%	97.6%	ND[c]	95.1%	96.3%	100.0%	96.3%	ND[c]	98.8%	98.8%	97.6%	97.3%
RNA15	unassigned	31	100.0%	97.3%	100.0%	100.0%	87.1%	100.0%	87.1%	96.8%	100.0%	100.0%	100.0%	87.1%	100.0%	100.0%	100.0%	83.9%	100.0%	100.0%	87.1%	100.0%	100.0%	100.0%	100.0%	100.0%	100.0%	97.2%
RNA16	unassigned	31	96.8%	97.4%	100.0%	96.8%	96.8%	96.8%	96.8%	96.8%	93.5%	96.8%	96.8%	96.8%	96.8%	96.8%	96.8%	96.8%	96.8%	*90.3%*	96.8%	100.0%	96.8%	96.8%	96.8%	96.8%	96.8%	96.8%
RNA20	unassigned	38	92.1%	100.0%	100.0%	92.1%	97.4%	97.4%	97.4%	100.0%	97.4%	97.4%	97.4%	97.4%	100.0%	97.4%	97.4%	97.4%	100.0%	100.0%	94.7%	100.0%	97.4%	97.4%	97.4%	97.4%	92.1%	97.4%
RNA21	unassigned	36	97.2%	100.0%	100.0%	97.2%	100.0%	97.2%	100.0%	97.2%	100.0%	94.4%	97.2%	100.0%	100.0%	91.7%	94.4%	100.0%	100.0%	97.2%	100.0%	100.0%	94.4%	97.2%	97.2%	97.2%	97.2%	97.9%
RNA22	unassigned	38	97.4%	100.0%	97.4%	94.7%	94.7%	94.7%	94.7%	94.7%	92.1%	94.7%	92.1%	94.7%	94.7%	92.1%	94.7%	92.1%	94.7%	100.0%	100.0%	97.4%	100.0%	94.7%	94.7%	94.7%	97.4%	95.5%
**total unassigned RNAs**		**328**	**94.8%**	**99.1%**	**93.9%**	**93.9%**	**99.4%**	**95.4%**	**93.9%**	**95.4%**	**95.1%**	**93.9%**	**96.3%**	**96.3%**	**94.8%**	**96.0%**	**93.3%**	**93.0%**	**96.0%**	**95.1%**	**93.9%**	**99.7%**	**96.3%**	**96.6%**	**95.7%**	**94.8%**	**94.8%**	**95.5%**
**total small mt RNAs**		**2365**	**95.7%**	**98.7%**	**95.4%**	**96.0%**	**99.2%**	**96.1%**	**96.5%**	**95.2%**	**95.8%**	**96.4%**	**95.9%**	**96.6%**	**95.6%**	**96.3%**	**95.5%**	**95.7%**	**96.4%**	**95.7%**	**96.1%**	**99.5%**	**96.2%**	**96.4%**	**96.0%**	**95.9%**	**95.7%**	**96.3%**
COB[d]	identical	376	89%	98%	99%	90%	91%	91%	91%	83%	91%	83%	91%	91%	88%	83%	91%	91%	90%	82%	91%	100%	83%	91%	91%	91%	89%	90%
	conserved		96%	99%	100%	97%	97%	97%	97%	91%	97%	92%	97%	97%	96%	91%	97%	97%	97%	92%	96%	100%	92%	97%	97%	97%	96%	96%
COX1[d]	identical	477[e]	93%	96%	98%	92%	94%	94%	94%	94%	94%	94%	93%	94%	93%	94%	94%	92%	93%	94%	95%	99%	94%	94%	94%	94%	92%	94%
	conserved		98%	99%	99%	97%	98%	98%	98%	98%	98%	97%	98%	98%	98%	98%	98%	98%	98%	98%	99%	100%	98%	98%	98%	98%	98%	98%
COX3[d]	identical	250[e]	84%	86%	88%	83%	84%	84%	86%	84%	85%	84%	84%	83%	84%	82%	85%	84%	83%	83%	81%	94%	84%	86%	84%	84%	84%	85%
	conserved		91%	96%	97%	91%	92%	92%	92%	92%	93%	93%	92%	92%	92%	92%	92%	92%	92%	92%	91%	98%	93%	93%	93%	93%	91%	93%

[a] Linear order of rRNA fragments, based on similarity to conventional rRNAs.

[b] Sample collected from a mandrill, species not identified.

[c] ND, not done. RNA7 similarity was not calculated for species from which its sequence is largely missing, presumably due to placement of PCR primers.

[d] Amino acid sequence similarity is shown. We calculated similarity on the basis of identity, and also allowing for conservative amino acid replacements.

[e] *P. falciparum cox1* and *cox3* lack conventional initiation codons and are presumed to use non-standard intiation codons [1] , common for mitochondrial proteins.
