# Supplementary material for: The Fragmented Mitochondrial Ribosomal RNAs of Plasmodium falciparum
Source: PLoS One. 2012 Jun 22;7(6):e38320. doi: 10.1371/journal.pone.0038320 (PMC3382252; doi:10.1371/journal.pone.0038320)
Supplement: Table S4 — Oligonucleotides used in this study. (PDF) [file pone.0038320.s020.pdf]

Table S4. Oligonucleotides used in this study.

Analysis	Gene	RACE	Primer Sequence	Position[a]
**RACE Analysis**	SSUB	5’ cDNA	GTGTTCCACCGCTAGTGTTTGC	431-452
		5’ PCR	CCACTTGCTTATAACTGTATGGACG	463-487
	SSUE	5’	CCAACAACATAACATTTTTTAGTCCCA	1794-1820
	RNA1	3’	GCTGACTTGAGTAATGATA	559-578 rc
	RNA4	3’	CATTTCTGAGTATTGAGCGGAAC	4647-4669
	RNA6	5’ cDNA	CTTGCCAACTCCCTATCATGTC	4839-4860 rc
		5’ PCR	GTCTTGCTAACGGCTTGTACGG	4820-4841 rc
		3’	CCGTACAAGCCGTTAGCAAGACA	4820-4843
	RNA9	5’	CAATCAAATTGGATGGTGTTGGC	83-105rc
		3’	CCTAATTTACGGGTCGGTTGTGG	69-91 rc
	RNA10	3’	GTACGAATAGACAATTGTGTTCATAGCTA	664-691
	RNA11	5’	TTCAATTCGTACTTCCACTACCAG	5279-5302
		3’	CATCGATATACGGATTTCTCCTG	5348-5370 rc
	RNA12	3’	GGGATATTTGTAGTACACCTTGATTGG	4911-4937
	RNA13	3’	GGGAAGTTTAGCCAGGAAGTCAGC	5001-5024 rc
	RNA14	5’	CGAGTCGATCAGGAAGGTTTC	5519-5539
		3’	GATGAAACCTTCCTGATCGACTCG	5519-5542 rc
	RNA15	3’	CACACTTCCCTTCTCGCC	606-623
	RNA16	5’ cDNA	GGATGGTGTTGGCTGGGC	78-95 rc
		5’ PCR	GGTATCTCGTAATGTAGAACAA	9-30
		3’	AAGCTTTTGGTATCTCGTAATGTAG	1-25
	RNA17	3’	CTGTGTTACAAATTTTTGATCCCAGG	114-139
	RNA18	3’	CGGTATTGCATGCCTGGTG	4968-4986
	RNA19	3’	GTTCTTATGTGTTGGCATGG	5557-5576 rc
	RNA20	5’ cDNA	GGATGGTGTTGGCTGGGC	78-95 rc
		5’ PCR	GAAAAGGATTTGACGGTCAACTC	35-57 rc
		3’	TGAGTTGACCGTCAAATCCTTTTCA	34-58
	RNA21[b]	3’	GCATGGGACTAAAAAATGTTATGTTGTTG	1791-1820
		5’	CCAACAACATAACATTTTTTAGTCCCA	1794-1821 rc
	RNA22	5’	CAGGAGAAATCCGTATATCGATG	5348-5370
		3’	CATCGATATACGGATTTCTCCTG	5348-5370 rc
**Primer Extension**	LSUB		TGATTACAGCTCCCAAGCAAAC	4594-4615
	LSUC		GAGCTATGACGCTATC	206-221
	LSUF		GAGCTCTATATATACTATAACC	1553-1574 rc
	RNA6		GTCTTGCTAACGGCTTGTACGG	4820-4842 rc
	RNA11		CAGGAGAAATCCGTATATCGATG	5348-5370

Analysis	Gene	RACE	Primer Sequence	Position[a]
	RNA12		CAATCAAGGTGTACTACAAATATC	4913-4936 rc
	RNA13		GACGCTGACTTCCTGGCT	4998-5015
	RNA14/RNA15		GTTTCTTTTACCTCACGAGTCGATC	5504-5528
**RNA Blots**	LSUB		TGATTACAGCTCCCAAGCAAAC	4594-4615
	LSUC		GAGCTATGACGCTATC	206-221
	LSUF		GAGCTCTATATATACTATAACC	1553-1574 rc
	RNA12		CAATCAAGGTGTACTACAAATATC	4913-4936 rc
	RNA13		GACGCTGACTTCCTGGCT	4998-5015
	RNA14		GTTTCTTTTACCTCACGAGTCGATC	5504-5528
	RNA15		TTCTATGGAAACACACTTC	595-613
	RNA16		GTTCTACATTACGAGATACCAAAAGC	3-28 rc

[a] Position is given per Genbank entry M76611. rc, reverse complement.

[b] The 5’ RACE primer for RNA21 overlapped the transcript start but generated products which correspond to the ends of upstream RNAs.
