# Supplementary material for: The Fragmented Mitochondrial Ribosomal RNAs of Plasmodium falciparum
Source: PLoS One. 2012 Jun 22;7(6):e38320. doi: 10.1371/journal.pone.0038320 (PMC3382252; doi:10.1371/journal.pone.0038320)
Supplement: Table S5 — P. falciparum mt rRNA 3′ end sequences. (PDF) [file pone.0038320.s021.pdf]

Table S5. ***Plasmodium falciparum*** **mt rRNA 3'end sequences**

Fragment	Sequence
SSUA	***GTATTATCCA TCCATGTCAG GCGTTAAAAG CGTTCGTTCT TATAGTGTAG***
	GUAUUAUCCA UCCAUGUCAG GCGUUAAAAG CGUUCGUUCU AAAAAAA
	GUAUUAUCCA UCCAUGUCAG GCGUUAAAAG CGUUCGUUCU AAAAAAAAAAAAA (2)
	GUAUUAUCCA UCCAUGUCAG GCGUUAAAAG CGUUCGUUCU AAAAAAAAAAAAAAAAAA
SSUB	***TTGTTTCATT TGATAGTAAA CACTATACCT TACCAATCTA TTTGAACTTG***
	UUGUUUCAUU UGAUAGUAAA CACUAUACCU UACCAAUCUA (4)
SSUD	***GACGTCAGGA AGTCCTGGAC GTTGAATCCA ATAGCATTGA TTAAAAGACA***
	GACGUCAGGA AGUCCUGGAC GUUGAAUCCA AUAGCAUUGA AAA
	GACGUCAGGA AGUCCUGGAC GUUGAAUCCA AUAGCAUUGA AA
	GACGUCAGGA AGUCCUGGAC GUUGAAUCCA AUAGCAUUGA
SSUF	***AACATGGTAG TTGACAGTGA ACTTGTAGCT GAACCAAAAA TGGCTGCTGG***
	AACAUGGUAG UUGACAGUGA ACUUGUAGCU GAACCAAAAA
	AACAUGGUAG UUGACAGUGA ACUUGUAGCU GAACCAAAAA AAA
	AACAUGGUAG UUGACAGUGA ACUUGUAGCU GAACCAAAAA AAAAAA (2)
LSUA	***TAGACGGTTT TCTGCGAAAT CTATTTGGAA GATATATCAT TGGGAAGTTT***
	UAGACGGUUU UCUGCGAAAU CUAUUUGGAA GAUAUAUCAU AAAAAAAAAAAAA
LSUD	***TGATGAATAT TTCAAGTTAC TGACATCTGC CCGGCATCAA TGATAAACGG***
	UGAUGAAUAU UUCAAGUUAC UGACAUCUGC CCGGCAUCAA AA
	UGAUGAAUAU UUCAAGUUAC UGACAUCUGC CCGGCAUCAA AAA
LSUE	***GCCCGACGGT AAGACCCTGA GCACCTTAAC TTCCCTAAAA GTTCTTATGT***
	GCCCGACGGU AAGACCCUGA GCACCUUAAC UUCCCUAAAA AAAAAAAAAAAAAAA
LSUF	***CTATTGGCAC CTCCATGTCG TCTCATCGCA GCCTTGCAAT AAATAATATC***
	CUAUUGGCAC CUCCAUGUCG UCUCAUCGCA GCCUUGCAAU AAAAAA
	CUAUUGGCAC CUCCAUGUCG UCUCAUCGCA GCCUUGCAAU AAAAAAAA
	CUAUUGGCAC CUCCAUGUCG UCUCAUCGCA GCCUUGCAAU AAAAAAAAA
LSUG	***AGAACGTCTT GAGGCAGTTT GTTCCCTATC TACCGTTTTA TCTTTGCATG***
	AGAACGUCUU GAGGCAGUUU GUUCCCUAUC UACCGUUUUA AAAAAAAAAAA
	AGAACGUCUU GAGGCAGUUU GUUCCCUAUC UACCGUUUUA AAAAAAAAAAAA
RNA1	***AGCTATCCAT AGTTAATTGA TTCCGTTTTG ACCGGTCATT TTCTTTGCCT***
	AGCUAUCCAU AGUUAAUUGA UUCCGUUUUG ACCGGUCAUU AAAA
RNA2	***TGAGATGGAA ACAGCCGGAA AGGTAATTTT ACGCCCTTAA CGTAAAGATC***
	UGAGAUGGAA ACAGCCGGAA AGGUAAUUUU ACGCCCUUAA AAAAAAAAAA
	UGAGAUGGAA ACAGCCGGAA AGGUAAUUUU ACGCCCUUAA AAAAAAAAAAAAAAA
	UGAGAUGGAA ACAGCCGGAA AGGUAAUUUU ACGCCCUUAA AAAAAAAAAAAAAAAAAAAAA
RNA3	***CTGTGAGGAA ACTACATTAA AGGAACTCGA CTGGCCTACA CTATAAGAAG***
	CUGUGAGGAA ACUACAUUAA AGGAACUCGA CUGGCCUACA AAAAAA (2)

Fragment	Sequence
	CUGUGAGGAA ACUACAUUAA AGGAACUCGA CUGGCCUACA AAAAAAAA (2)
RNA4	***GATTGAGCGG AACAAATCAG ACCGTAAGGT TATAATTATG TACTATGATT***
	GAUUGAGCGG AACAAAUCAG ACCGUAAGGU UAUAAUUAUG AAA (2)
RNA5	***TTACAGTATC AATCGGATTT ACATGCTCAG CCGCCAAAAA CTATAACGAT***
	UUACAGUAUC AAUCGGAUUU ACAUGCUCAG CCGCCAAAAA AAAAAAAAAA
	UUACAGUAUC AAUCGGAUUU ACAUGCUCAG CCGCCAAAAA AAAAAA
RNA6	***AAGCCGTTAG CAAGACATGA TAGGGAGTTG GCAAGTTAAA GAAGTTCTG***
	AAGCCGUUAG CAAGACAUGA UAGGGAGUUG GCAAGUUAAA A
	AAGCCGUUAG CAAGACAUGA UAGGGAGUUG GCAAGUUAAA AA (2)
RNA7	***AATCGAGAGA GATTCCATTA GTTGTCTCTA TGAATAGTGG TTATAGCCAT***
	AAUCGAGAGA GAUUCCAUUA GUUGUCUCUA UGAAUAGUGG AAAAAAAAAA
	AAUCGAGAGA GAUUCCAUUA GUUGUCUCUA UGAAUAGUGG AAAAAAAAAAAAAAA (2)
RNA8	***CCCGGGAAAC CGGCGCTTCC ATTTATAAGA AGTTAAATTA CTGGAAGCGT***
	CCCGGGAAAC CGGCGCUUCC AUUUAUAAGA AGUUAAAUUA AAAAAAA
	CCCGGGAAAC CGGCGCUUCC AUUUAUAAGA AGUUAAAUUA AAAAAAAAAAAAA
	CCCGGGAAAC CGGCGCUUCC AUUUAUAAGA AGUUAAAUUA AAAAAAAAAAAAAAAA (3)
	CCCGGGAAAC CGGCGCUUCC AUUUAUAAGA AGUUAAAUUA AAAAAAAAAAAAAAAAAA
	CCCGGGAAAC CGGCGCUUCC AUUUAUAAGA AGUUAAAUUA AAAAAAAAAAAAAAAAAAA
RNA9	***AACACCATCC AATTTGATTG GGAATTATCT GTGTTACAAA TTTTTGATCC***
	AACACCAUCC AAUUUGAUUG GGAAUUAUCU GUGUUACAAA
	AACACCAUCC AAUUUGAUUG GGAAUUAUCU GUGUUACAAA A
	AACACCAUCC AAUUUGAUUG GGAAUUAUCU GUGUUACAAA AAA
	AACACCAUCC AAUUUGAUUG GGAAUUAUCU GUGUUACAAA AAAAAA
	AACACCAUCC AAUUUGAUUG GGAAUUAUCU GUGUUACAAA AAAAAAAAAA
	AACACCAUCC AAUUUGAUUG GGAAUUAUCU GUGUUACAAA AAAAAAAAAAAA
RNA10	***TAGAGTACGT AAGGAAAAGG AAAGGTTAAC CGCTATCAAA TGGCGAGAAG***
	UAGAGUACGU AAGGAAAAGG AAAGGUUAAC CGCUAUCAAA AAAAAAAAA
	UAGAGUACGU AAGGAAAAGG AAAGGUUAAC CGCUAUCAAA AAAAAAAA
RNA11	***TTTAGAACAG GAGAGTATAT TCTGGTAGTG GAAGTACGAA TTGAAGTGGA***
	UUUAGAACAG GAGAGUAUAU UCUGGUAGUG GAAGUACGAA AAAAAAAAA
RNA12	***TGTATGGGAT ATTTGTAGTA CACCTTGATT GGTTTTACTA TTTATACTTA***
	UGUAUGGGAU AUUUGUAGUA CACCUUGAUU GGUUUUACUA AAAAAAAAAAAA (2)
	UGUAUGGGAU AUUUGUAGUA CACCUUGAUU GGUUUUACUA AAAAAAAAAAAAA
	UGUAUGGGAU AUUUGUAGUA CACCUUGAUU GGUUUUACUA AAAAGAAAAAAAAAAAAA
	UGUAUGGGAU AUUUGUAGUA CACCUUGAUU GGUUUUACUA AAAAAAAAAAAAAAAAAAAAAAAAA
RNA13	***GATATATCAT TGGGAAGTTT AGCCAGGAAG TCAGCGTCTA TATTAAAAA***
	5′ UGGGAAGUUU AGCCAGGAAG UCAGCGUCUA AAAAAAAAAAAAAA

Fragment	Sequence
RNA14	***TTAAGGATGA AACCTTCCTG ATCGACTCGT GAGGTAAAAG AAACAGTCCG***
	5′ UUAAGGAUGA AACCUUCCUG AUCGACUCGU GAGGUAAAAG AA
	UUAAGGAUGA AACCUUCCUG AUCGACUCGU GAGGUAAAAG AAA
	UUAAGGAUGA AACCUUCCUG AUCGACUCGU GAGGUAAAAG AAAAAAAA
	UUAAGGAUGA AACCUUCCUG AUCGACUCGU GAGGUAAAAG AAAAAAAAAA
	UUAAGGAUGA AACCUUCCUG AUCGACUCGU GAGGUAAAAG AAAAAAAAAAAAAAA
RNA15	***GCTATCAAAT GGCGAGAAGG GAAGTGTGTT TCCATAGAAA CCTTGTATAT***
	5′ U GGCGAGAAGG GAAGUGUGUU UCCAUAGAAA AAAAAAAAA
RNA16	***TATAATAAAG CTTTTGGTAT CTCGTAATGT AGAACAATAT TGAGTTGACC***
	5′ G CUUUUGGUAU CUCGUAAUGU AGAACAAUAU A
	G CUUUUGGUAU CUCGUAAUGU AGAACAAUAU AA (3)
	G CUUUUGGUAU CUCGUAAUGU AGAACAAUAU AAAA (3)
	G CUUUUGGUAU CUCGUAAUGU AGAACAAUAU AAAAAAAAAAAAAA
RNA17	***TTTTTGATCC CAGGCTGGTA AAAAATGTAA ACTTTTAGCC CATAAGAATA***
	UUUUUGAUCC CAGGCUGGUA AAAAAUGUAA ACUUUUAGCC AAAAAAA
RNA18	***ATACTTATCG ATAAATGTTC GGTATTGCAT GCCTGGTGTT TTTAATATAG***
	5′ UGUUC GGUAUUGCAU GCCUGGUGUU AAAAAAAAA (2)
	UGUUC GGUAUUGCAU GCCUGGUGUU AAAAAAAAAAA
	UGUUC GGUAUUGCAU GCCUGGUGUU AAAAAAAAAAAA
	UGUUC GGUAUUGCAU GCCUGGUGUU AAAAAAAAAAAAAAA (2)
RNA19	***TTCCCTAAAA GTTCTTATGT GTTGGCATGG TTACGAGATT AAGGATGTTT***
	5′ GUUCUUAUGU GUUGGCAUGG UUACGAGAUU AAAAAAAA
	GUUCUUAUGU GUUGGCAUGG UUACGAGAUU AAAAAAAAA
	GUUCUUAUGU GUUGGCAUGG UUACGAGAUU AAAAAAAAAAAAA
	GUUCUUAUGU GUUGGCAUGG UUACGAGAUU AAAAAAAAAAAAAAA
	GUUCUUAUGU GUUGGCAUGG UUACGAGAUU AAAAAAAAAAAAAAAAAA (2)
RNA20	***ATTGAGTTGA CCGTCAAATC CTTTTCATTA AAAGAGTGGA TTAAATGCCC***
	5′ UGAGUUGA CCGUCAAAUC CUUUUCAUUA AAAGAGUGGA AAAAAAAAAAA
	UGAGUUGA CCGUCAAAUC CUUUUCAUUA AAAGAGUGGA AAAAAAAAAAAAAAAAAAAA (2)
RNA21	***AGCATGGGAC TAAAAAATGT TATGTTGTTG GTTTAAGCCC TATTACCATA***
	AGCATGGGAC TAAAAAATGT TATGTTGTTG GTTTAAGCCC AAAAAA
	AGCATGGGAC TAAAAAATGT TATGTTGTTG GTTTAAGCCC AAAAAAA (2)
	AGCATGGGAC TAAAAAATGT TATGTTGTTG GTTTAAGCCC AAAAAAAA
RNA22	***GATTAAAAGA CATCGATATA CGGATTTCTC CTGAAAAAAC GCGAAAAACC***
	5′ UUAAAAGA CAUCGAUAUA CGGAUUUCUC CUGAAAAAAC AA

The top sequence (in bold italics) for each set of RACE data corresponds to the genomic DNA sequence. Numbers in parentheses indicate how often that size of tail was found.
