# Supplementary material for: The Fragmented Mitochondrial Ribosomal RNAs of Plasmodium falciparum
Source: PLoS One. 2012 Jun 22;7(6):e38320. doi: 10.1371/journal.pone.0038320 (PMC3382252; doi:10.1371/journal.pone.0038320)
Supplement: Table S6 — Abutted P. falciparum mt gene sets. (PDF) [file pone.0038320.s022.pdf]

Table S6. Abutted *P. falciparum* mt gene sets

Description	Members	Size (nt)
RNA16 to RNA17	4	163
RNA8 to RNA13	12	959
cox3 to LSUG	6[a]	1205
cox1 to RNA4	3	2660

[a] If RNA23t through RNA27t are included, this run is *cox3* to RNA23t, 10 members, 1317 nt.
