# Supplementary material for: The Fragmented Mitochondrial Ribosomal RNAs of Plasmodium falciparum
Source: PLoS One. 2012 Jun 22;7(6):e38320. doi: 10.1371/journal.pone.0038320 (PMC3382252; doi:10.1371/journal.pone.0038320)
Supplement: Table S7 — Fragment placement in rRNA alignments for covariation analysis. (PDF) [file pone.0038320.s023.pdf]

## Table S7. Fragment placement in rRNA alignments for covariation analysis

| Fragment[a] | rRNA[b] | Hemosporidians[c] | Coccidian[d] | Piroplasms[e] |
|---|---|---|---|---|
| **SSUA** | SSU | +[f] | + | + |
| **SSUB** | SSU | + | + | + |
| **SSUD** | SSU | + | + | + |
| **SSUE** | SSU | + | + | + |
| **SSUF** | SSU | + | + | + |
| **LSUA** | LSU | + | + | + |
| **LSUB** | LSU | + | + | + |
| **LSUC** | LSU | + | + | + |
| **LSUD** | LSU | + | + | + |
| **LSUE** | LSU | + | + | + |
| **LSUF** | LSU | + | + | + |
| **LSUG** | LSU | + | + | + |
| **RNA1** | LSU | + | + | + |
| **RNA2** | LSU | + | + | + |
| **RNA3** | LSU | + | + | + |
| **RNA5** | SSU | + | | |
| **RNA6** | LSU | + | | + |
| **RNA8** | SSU | + | + | + |
| **RNA9** | SSU | + | | + |
| **RNA10** | LSU | + | + | + |
| **RNA11** | LSU | + | | + |
| **RNA12** | SSU | + | | |
| **RNA13** | LSU | + | + | + |
| **RNA14** | SSU | + | | + |
| **RNA17** | SSU | + | + | + |
| **RNA18** | LSU | + | + | + |
| **RNA19** | SSU | + | | |
| **COUNTS** | | 27 | 20 | 24 |

[a] Gene names are as defined in Table 1.

[b] LSU, large subunit rRNA; SSU, small subunit rRNA. Fragments not assigned: RNA4, RNA7, RNA15, RNA16, RNA20, RNA21, RNA22, RNA23t, RNA24t, RNA25t, RNA26t, RNA27t.

[c] RNAs from *Plasmodium* and related species that were used for co-variation analysis.

[d] RNAs from *Eimeria tenella* that were used for co-variation analysis.

[e] RNAs from *Babesia* and *Theileria* that were used for co-variation analysis.

[f] +, present; blank if absent.
