# Supplementary material for: The Fragmented Mitochondrial Ribosomal RNAs of Plasmodium falciparum
Source: PLoS One. 2012 Jun 22;7(6):e38320. doi: 10.1371/journal.pone.0038320 (PMC3382252; doi:10.1371/journal.pone.0038320)
Supplement: Table S8 — Prospective covariations found in fragmented apicomplexan mt rRNA genes. (PDF) [file pone.0038320.s024.pdf]

Table S8. Prospective covariations found in fragmented apicomplexan mt rRNA genes.

rRNA[a]	Pair[b] (Fragments[c])	Category[d]	Hemosporidian[e]	+ Coccidian[f]	+ Piroplasm[g]
LSU	581,1259<br>(LSUA,RNA1)	2	ug=(16, 16.00, 94.1%) [h, i]<br>ag=(1, 1.00, 5.9%)	ug=(16, 16.00, 94.1%) [j]<br>ag=(1, 1.00, 5.9%)	ug=(16, 12.36, 72.7%)<br>aa=(5, 1.36, 22.7%)<br>ag=(1, 4.64, 4.5%)
LSU	583,1257<br>(LSUA,RNA1)	2	ac=(192, 192.00, 97.0%)<br>gc=(6, 6.00, 3.0%)	ac=(192, 192.00, 97.0%)<br>gc=(6, 6.00, 3.0%)	ac=(192, 184.54, 93.2%)<br>uu=(8, 0.31, 3.9%)<br>gc=(6, 5.77, 2.9%)
LSU	589,668<br>(LSUA)	2	cg=(198, 198.00, 100.0%)	cg=(199, 199.00, 100.0%)	cg=(199, 191.31, 96.1%)<br>au=(5, 0.19, 2.4%)<br>gu=(3, 0.12, 1.4%)
LSU	590,667<br>(LSUA)	2	au=(198, 198.00, 100.0%)	au=(198, 198.00, 99.5%)<br>aa=(1, 1.00, 0.5%)	au=(198, 190.35, 95.7%)<br>ua=(8, 0.35, 3.9%)<br>aa=(1, 8.65, 0.5%)
LSU	591,666<br>(LSUA)	2	ua=(198, 198.00, 100.0%)	ua=(198, 198.00, 99.5%)<br>aa=(1, 1.00, 0.5%)	ua=(198, 190.35, 95.7%)<br>au=(8, 0.35, 3.9%)<br>aa=(1, 8.65, 0.5%)
LSU	672,808<br>(LSUA)	1	ug=(198, 198.00, 100.0%)	ug=(199, 199.00, 100.0%)	ug=(199, 191.31, 96.1%)<br>au=(8, 0.31, 3.9%)
LSU	679,798<br>(LSUA)	2	cg=(200, 200.00, 98.5%)<br>-g=(3, 3.00, 1.5%)	cg=(201, 201.00, 98.5%)<br>-g=(3, 3.00, 1.5%)	cg=(201, 193.42, 94.8%)<br>aa=(8, 0.30, 3.8%)<br>-g=(3, 2.89, 1.4%)
LSU	680,797<br>(LSUA)	2	au=(200, 200.00, 98.5%)<br>-u=(3, 3.00, 1.5%)	au=(201, 201.00, 98.5%)<br>-u=(3, 3.00, 1.5%)	au=(201, 193.42, 94.8%)<br>ua=(8, 0.30, 3.8%)<br>-u=(3, 2.89, 1.4%)
LSU	693,769<br>(LSUA)	2	au=(200, 200.00, 98.5%)<br>-u=(3, 3.00, 1.5%)	au=(200, 200.00, 98.0%)<br>-u=(3, 3.00, 1.5%)<br>uu=(1, 1.00, 0.5%)	au=(200, 192.45, 94.3%)<br>ua=(8, 0.34, 3.8%)<br>-u=(3, 2.89, 1.4%)<br>uu=(1, 8.66, 0.5%)
LSU	694,768<br>(LSUA)	2	cg=(200, 200.00, 98.5%)<br>-g=(3, 3.00, 1.5%)	cg=(200, 200.00, 98.0%)<br>-g=(3, 3.00, 1.5%)<br>ag=(1, 1.00, 0.5%)	cg=(200, 192.45, 94.3%)<br>ua=(8, 0.30, 3.8%)<br>-g=(3, 2.89, 1.4%)<br>ag=(1, 0.96, 0.5%)

rRNA[a]	Pair[b] (Fragments[c])	Category[d]	Hemosporidian[e]	+ Coccidian[f]	+ Piroplasm[g]
LSU	695,767 (LSUA)	2	gc=(200, 200.00, 98.5%)<br>-c=(3, 3.00, 1.5%)	gc=(200, 200.00, 98.0%)<br>-c=(3, 3.00, 1.5%)<br>ac=(1, 1.00, 0.5%)	gc=(200, 192.45, 94.3%)<br>au=(8, 0.34, 3.8%)<br>-c=(3, 2.89, 1.4%)<br>ac=(1, 8.66, 0.5%)
LSU	696,766 (LSUA)	2	au=(200, 200.00, 98.5%)<br>-u=(3, 3.00, 1.5%)	au=(201, 201.00, 98.5%)<br>-u=(3, 3.00, 1.5%)	au=(201, 193.42, 94.8%)<br>ua=(8, 0.30, 3.8%)<br>-u=(3, 2.89, 1.4%)
LSU	697,765 (LSUA)	1	au=(203, 203.00, 100.0%)	au=(204, 204.00, 100.0%)	au=(204, 196.30, 96.2%)<br>ua=(8, 0.30, 3.8%)
LSU	698,763 (LSUA)	2	cg=(203, 203.00, 97.1%)<br>ug=(6, 6.00, 2.9%)	cg=(204, 204.00, 97.1%)<br>ug=(6, 6.00, 2.9%)	cg=(204, 198.39, 93.6%)<br>ug=(8, 8.75, 3.7%)<br>au=(5, 0.11, 2.3%)<br>ua=(1, 0.04, 0.5%)
LSU	700,732 (LSUA)	2	gc=(209, 209.00, 100.0%)	gc=(210, 210.00, 100.0%)	gc=(210, 205.19, 96.3%)<br>au=(5, 0.19, 2.3%)<br>ac=(2, 6.81, 0.9%)<br>gu=(1, 5.81, 0.5%)
LSU	701,731 (LSUA)	1	gc=(209, 209.00, 100.0%)	gc=(210, 210.00, 100.0%)	gc=(210, 202.29, 96.3%)<br>ua=(8, 0.29, 3.7%)
LSU	741,756 (LSUA)	1	gc=(209, 209.00, 100.0%)	gc=(210, 210.00, 100.0%)	gc=(210, 202.29, 96.3%)<br>cg=(8, 0.29, 3.7%)
LSU	744,753 (LSUA)	1	ua=(209, 209.00, 100.0%)	ua=(210, 210.00, 100.0%)	ua=(210, 202.29, 96.3%)<br>cg=(8, 0.29, 3.7%)
LSU	779,785 (LSUA)	1	ug=(209, 209.00, 100.0%)	ug=(210, 210.00, 100.0%)	ug=(210, 202.29, 96.3%)<br>au=(8, 0.29, 3.7%)
LSU	824,833 (LSUA)	1	ua=(208, 207.00, 99.5%)<br>au=(1, 0.00, 0.5%)	ua=(209, 208.00, 99.5%)<br>au=(1, 0.00, 0.5%)	ua=(217, 216.00, 99.5%)<br>au=(1, 0.00, 0.5%)
LSU	946,971 (RNA2)	1	au=(209, 209.00, 100.0%)	au=(209, 208.00, 99.5%)<br>gc=(1, 0.00, 0.5%)	au=(209, 200.37, 95.9%)<br>gc=(9, 0.37, 4.1%)

rRNA[a]	Pair[b] (Fragments[c])	Category[d]	Hemosporidian[e]	+ Coccidian[f]	+ Piroplasm[g]
LSU	950,967 (RNA2)	1	cg=(208, 207.00, 99.5%)<br>ua=(1, 0.00, 0.5%)	cg=(209, 208.00, 99.5%)<br>ua=(1, 0.00, 0.5%)	cg=(209, 200.37, 95.9%)<br>au=(8, 0.29, 3.7%)<br>ua=(1, 0.00, 0.5%)
LSU	953,964 (RNA2)	2	gg=(209, 209.00, 100.0%)	gg=(209, 209.00, 99.5%)<br>ug=(1, 1.00, 0.5%)	gg=(209, 201.33, 95.9%)<br>ua=(8, 0.33, 3.7%)<br>ug=(1, 8.67, 0.5%)
LSU	976,987 (RNA2)	1	ug=(209, 209.00, 100.0%)	ug=(210, 210.00, 100.0%)	ug=(210, 202.29, 96.3%)<br>au=(6, 0.17, 2.8%)<br>gc=(2, 0.02, 0.9%)
LSU	977,986 (RNA2)	1	gc=(209, 209.00, 100.0%)	gc=(210, 210.00, 100.0%)	gc=(210, 202.29, 96.3%)<br>cg=(8, 0.29, 3.7%)
LSU	1002,1153 (RNA2,RNA11)	1	cg=(209, 209.00, 100.0%)	cg=(209, 209.00, 100.0%)	cg=(209, 201.29, 96.3%)<br>aa=(8, 0.29, 3.7%)
LSU	1164,1185 (RNA11)	1	au=(209, 209.00, 100.0%)	au=(209, 209.00, 100.0%)	au=(209, 201.29, 96.3%)<br>ua=(8, 0.29, 3.7%)
LSU	1166,1183 (RNA11)	2	au=(209, 209.00, 100.0%)	au=(209, 209.00, 100.0%)	au=(209, 203.22, 96.3%)<br>gc=(6, 0.22, 2.8%)<br>gu=(2, 7.78, 0.9%)
LSU	1196,1250 (RNA11,RNA1)	2	gu=(207, 207.00, 99.0%)<br>uu=(2, 2.00, 1.0%)	gu=(207, 207.00, 99.0%)<br>uu=(2, 2.00, 1.0%)	gu=(207, 199.37, 95.4%)<br>ua=(8, 0.37, 3.7%)<br>uu=(2, 9.63, 0.9%)
LSU	1198,1247 (RNA11,RNA1)	2	ua=(208, 208.00, 99.5%)<br>ug=(1, 1.00, 0.5%)	ua=(208, 208.00, 99.5%)<br>ug=(1, 1.00, 0.5%)	ua=(208, 200.33, 95.9%)<br>au=(8, 0.29, 3.7%)<br>ug=(1, 0.96, 0.5%)
LSU	1656,2004 (RNA3,LSUE)	2	ca=(202, 202.00, 96.7%)<br>ua=(6, 6.00, 2.9%)<br>aa=(1, 1.00, 0.5%)	ca=(203, 203.00, 96.7%)<br>ua=(6, 6.00, 2.9%)<br>aa=(1, 1.00, 0.5%)	ca=(203, 195.55, 93.1%)<br>au=(8, 0.33, 3.7%)<br>ua=(6, 5.78, 2.8%)<br>aa=(1, 8.67, 0.5%)
LSU	1658,2002 (RNA3,LSUE)	1	au=(209, 209.00, 100.0%)	au=(210, 210.00, 100.0%)	au=(210, 202.29, 96.3%)<br>ua=(8, 0.29, 3.7%)

rRNA[a]	Pair[b] (Fragments[c])	Category[d]	Hemosporidian[e]	+ Coccidian[f]	+ Piroplasm[g]
LSU	1659,2001 (RNA3,LSUE)	2	cg=(198, 189.47, 94.7%)<br>ua=(4, 0.11, 1.9%)<br>au=(4, 0.08, 1.9%)<br>ug=(2, 5.74, 1.0%)<br>gc=(1, 0.00, 0.5%)	cg=(199, 190.47, 94.8%)<br>ua=(4, 0.11, 1.9%)<br>au=(4, 0.08, 1.9%)<br>ug=(2, 5.74, 1.0%)<br>gc=(1, 0.00, 0.5%)	cg=(199, 183.48, 91.3%)<br>ua=(12, 0.77, 5.5%)<br>au=(4, 0.07, 1.8%)<br>ug=(2, 12.91, 0.9%)<br>gc=(1, 0.00, 0.5%)
LSU	1661,1999 (RNA3,LSUE)	2	ug=(209, 209.00, 100.0%)	ug=(209, 209.00, 99.5%)<br>ua=(1, 1.00, 0.5%)	ug=(209, 201.33, 95.9%)<br>gc=(8, 0.29, 3.7%)<br>ua=(1, 0.96, 0.5%)
LSU	1663,1997 (RNA3,LSUE)	1	au=(209, 209.00, 100.0%)	au=(210, 210.00, 100.0%)	au=(210, 202.29, 96.3%)<br>gc=(8, 0.29, 3.7%)
LSU	1766,1986 (LSUD,LSUE)	1	gc=(209, 209.00, 100.0%)	gc=(210, 210.00, 100.0%)	gc=(210, 202.29, 96.3%)<br>au=(8, 0.29, 3.7%)
LSU	1767,1985 (LSUD,LSUE)	1	gc=(208, 207.00, 99.5%)<br>au=(1, 0.00, 0.5%)	gc=(208, 206.02, 99.0%)<br>au=(2, 0.02, 1.0%)	gc=(216, 214.02, 99.1%)<br>au=(2, 0.02, 0.9%)
LSU	1794,1825 (LSUD)	1	ua=(209, 209.00, 100.0%)	ua=(209, 208.00, 99.5%)<br>au=(1, 0.00, 0.5%)	ua=(209, 200.37, 95.9%)<br>au=(9, 0.37, 4.1%)
LSU	1795,1824 (LSUD)	2	cu=(208, 208.00, 99.5%)<br>uu=(1, 1.00, 0.5%)	cu=(209, 209.00, 99.5%)<br>uu=(1, 1.00, 0.5%)	cu=(213, 209.09, 97.7%)<br>ua=(3, 0.07, 1.4%)<br>uc=(1, 0.02, 0.5%)<br>uu=(1, 4.91, 0.5%)
LSU	1797,1822 (LSUD)	1	cg=(209, 209.00, 100.0%)	cg=(210, 210.00, 100.0%)	cg=(210, 202.29, 96.3%)<br>au=(8, 0.29, 3.7%)
LSU	1800,1817 (LSUD)	1	au=(209, 209.00, 100.0%)	au=(209, 208.00, 99.5%)<br>cg=(1, 0.00, 0.5%)	au=(209, 200.37, 95.9%)<br>cg=(9, 0.37, 4.1%)
LSU	1804,1813 (LSUD)	2	ua=(202, 201.03, 96.7%)<br>ca=(6, 6.97, 2.9%)<br>cg=(1, 0.03, 0.5%)	ua=(202, 200.08, 96.2%)<br>ca=(6, 7.92, 2.9%)<br>cg=(2, 0.08, 1.0%)	ua=(202, 192.73, 92.7%)<br>gc=(8, 0.29, 3.7%)<br>ca=(6, 7.63, 2.8%)<br>cg=(2, 0.07, 0.9%)
LSU	1805,1812 (LSUD)	1	ua=(209, 209.00, 100.0%)	ua=(210, 210.00, 100.0%)	ua=(210, 202.29, 96.3%)<br>au=(8, 0.29, 3.7%)

rRNA[a]	Pair[b] (Fragments[c])	Category[d]	Hemosporidian[e]	+ Coccidian[f]	+ Piroplasm[g]
LSU	1806,1811 (LSUD)	2	cg=(208, 207.00, 99.5%)<br>ua=(1, 0.00, 0.5%)	cg=(208, 207.01, 99.0%)<br>ua=(1, 0.01, 0.5%)<br>ug=(1, 1.99, 0.5%)	cg=(208, 199.41, 95.4%)<br>ua=(9, 0.41, 4.1%)<br>ug=(1, 9.59, 0.5%)
LSU	1830,1975 (LSUD,LSUE)	1	cg=(209, 209.00, 100.0%)	cg=(210, 210.00, 100.0%)	cg=(211, 204.22, 96.8%)<br>ua=(7, 0.22, 3.2%)
LSU	1831,1974 (LSUD,LSUE)	1	au=(209, 209.00, 100.0%)	au=(210, 210.00, 100.0%)	au=(217, 216.00, 99.5%)<br>gc=(1, 0.00, 0.5%)
LSU	1836,1904 (LSUD,LSUE)	1	cg=(209, 209.00, 100.0%)	cg=(210, 210.00, 100.0%)	cg=(210, 206.07, 98.1%)<br>uu=(4, 0.07, 1.9%)
LSU	1837,1903 (LSUD,LSUE)	1	cg=(209, 209.00, 100.0%)	cg=(210, 210.00, 100.0%)	cg=(210, 206.07, 98.1%)<br>uu=(4, 0.07, 1.9%)
LSU	1843,1897 (LSUD,LSUE)	1	ua=(209, 209.00, 100.0%)	ua=(209, 208.00, 99.5%)<br>cg=(1, 0.00, 0.5%)	ua=(209, 208.00, 99.5%)<br>cg=(1, 0.00, 0.5%)
LSU	1906,1924 (LSUE)	2	gc=(209, 209.00, 100.0%)	gc=(210, 210.00, 100.0%)	gc=(210, 206.07, 98.1%)<br>a-=(2, 0.02, 0.9%)<br>ua=(2, 0.02, 0.9%)
LSU	1907,1923 (LSUE)	2	gu=(209, 209.00, 100.0%)	gu=(210, 210.00, 100.0%)	gu=(210, 205.12, 97.7%)<br>cg=(2, 0.05, 0.9%)<br>ug=(2, 0.05, 0.9%)<br>ag=(1, 0.02, 0.5%)
LSU	1909,1921 (LSUE)	2	ug=(209, 209.00, 100.0%)	ug=(210, 210.00, 100.0%)	ug=(211, 204.22, 96.8%)<br>au=(2, 0.05, 0.9%)<br>a-=(2, 0.05, 0.9%)<br>ca=(2, 0.03, 0.9%)<br>aa=(1, 0.07, 0.5%)
LSU	1925,1929 (LSUE)	2	cg=(209, 209.00, 100.0%)	cg=(210, 210.00, 100.0%)	cg=(210, 202.29, 96.3%)<br>ac=(3, 0.04, 1.4%)<br>uu=(3, 0.07, 1.4%)<br>ua=(2, 0.05, 0.9%)
LSU	1945,1961 (LSUE)	1	gc=(209, 209.00, 100.0%)	gc=(210, 210.00, 100.0%)	gc=(210, 202.29, 96.3%)<br>au=(8, 0.29, 3.7%)

rRNA[a]	Pair[b] (Fragments[c])	Category[d]	Hemosporidian[e]	+ Coccidian[f]	+ Piroplasm[g]
LSU	1947,1959 (LSUE)	2	cg=(209, 209.00, 100.0%)	cg=(210, 210.00, 100.0%)	cg=(210, 203.26, 96.3%)<br>ua=(7, 0.26, 3.2%)<br>ug=(1, 7.74, 0.5%)
LSU	1948,1958 (LSUE)	1	gc=(209, 209.00, 100.0%)	gc=(210, 210.00, 100.0%)	gc=(214, 210.07, 98.2%)<br>au=(4, 0.07, 1.8%)
LSU	1949,1957 (LSUE)	1	gc=(208, 207.00, 99.5%)<br>au=(1, 0.00, 0.5%)	gc=(209, 208.00, 99.5%)<br>au=(1, 0.00, 0.5%)	gc=(209, 200.37, 95.9%)<br>ua=(8, 0.29, 3.7%)<br>au=(1, 0.00, 0.5%)
LSU	1950,1956 (LSUE)	2	gu=(206, 204.03, 98.6%)<br>aa=(2, 0.03, 1.0%)<br>ga=(1, 2.97, 0.5%)	gu=(207, 205.03, 98.6%)<br>aa=(2, 0.03, 1.0%)<br>ga=(1, 2.97, 0.5%)	gu=(207, 197.50, 95.0%)<br>ca=(8, 0.40, 3.7%)<br>aa=(2, 0.10, 0.9%)<br>ga=(1, 10.50, 0.5%)
LSU	2024,2039 (LSUE)	2	ua=(208, 208.00, 99.5%)<br>ug=(1, 1.00, 0.5%)	ua=(209, 209.00, 99.5%)<br>ug=(1, 1.00, 0.5%)	ua=(209, 201.33, 95.9%)<br>gc=(8, 0.29, 3.7%)<br>ug=(1, 0.96, 0.5%)
LSU	2025,2038 (LSUE)	1	cg=(209, 209.00, 100.0%)	cg=(210, 210.00, 100.0%)	cg=(213, 208.11, 97.7%)<br>au=(5, 0.11, 2.3%)
LSU	2026,2037 (LSUE)	1	cg=(209, 209.00, 100.0%)	cg=(210, 210.00, 100.0%)	cg=(210, 202.29, 96.3%)<br>ua=(8, 0.29, 3.7%)
LSU	2027,2036 (LSUE)	1	au=(209, 209.00, 100.0%)	au=(209, 208.00, 99.5%)<br>ua=(1, 0.00, 0.5%)	au=(209, 200.37, 95.9%)<br>ua=(9, 0.37, 4.1%)
LSU	2047,2621 (LSUE,LSUG)	2	cg=(208, 207.00, 99.5%)<br>ua=(1, 0.00, 0.5%)	cg=(208, 206.02, 99.0%)<br>ua=(2, 0.02, 1.0%)	cg=(208, 198.46, 95.4%)<br>ua=(9, 0.41, 4.1%)<br>uu=(1, 0.05, 0.5%)
LSU	2048,2620 (LSUE,LSUG)	2	gc=(209, 209.00, 100.0%)	gc=(210, 210.00, 100.0%)	gc=(210, 202.29, 96.3%)<br>au=(7, 0.26, 3.2%)<br>aa=(1, 0.04, 0.5%)
LSU	2049,2619 (LSUE,LSUG)	1	gc=(209, 209.00, 100.0%)	gc=(210, 210.00, 100.0%)	gc=(210, 202.29, 96.3%)<br>cg=(8, 0.29, 3.7%)
LSU	2080,2240 (LSUE,RNA13)	2	ua=(209, 209.00, 100.0%)	ua=(210, 210.00, 100.0%)	ua=(216, 215.01, 99.1%)<br>cg=(1, 0.01, 0.5%)<br>ug=(1, 1.99, 0.5%)

rRNA[a]	Pair[b] (Fragments[c])	Category[d]	Hemosporidian[e]	+ Coccidian[f]	+ Piroplasm[g]
LSU	2461,2489 (LSUF)	1	gc=(209, 209.00, 100.0%)	gc=(210, 210.00, 100.0%)	gc=(213, 208.11, 97.7%)<br>au=(5, 0.11, 2.3%)
LSU	2462,2488 (LSUF)	1	ua=(206, 203.04, 98.6%)<br>au=(3, 0.04, 1.4%)	ua=(206, 202.08, 98.1%)<br>au=(4, 0.08, 1.9%)	ua=(206, 194.66, 94.5%)<br>au=(12, 0.66, 5.5%)
LSU	2463,2487 (LSUF)	1	au=(209, 209.00, 100.0%)	au=(210, 210.00, 100.0%)	au=(210, 202.09, 96.3%)<br>ua=(8, 0.29, 3.7%)
LSU	2466,2484 (LSUF)	2	ua=(209, 209.00, 100.0%)	ua=(209, 209.00, 99.5%)<br>ug=(1, 1.00, 0.5%)	ua=(209, 201.33, 95.9%)<br>au=(8, 0.29, 3.7%)<br>ug=(1, 0.96, 0.5%)
LSU	2470,2480 (LSUF)	2	gc=(207, 205.02, 99.0%)<br>au=(2, 0.02, 1.0%)	gc=(208, 206.02, 99.0%)<br>au=(2, 0.02, 1.0%)	gc=(208, 198.46, 95.4%)<br>au=(4, 0.11, 1.8%)<br>ug=(3, 0.06, 1.4%)<br>aa=(2, 0.08, 0.9%)<br>ua=(1, 0.06, 0.5%)
LSU	2514,2570 (LSUF,LSUG)	1	ua=(209, 209.00, 100.0%)	ua=(210, 210.00, 100.0%)	ua=(210, 202.29, 96.3%)<br>cg=(8, 0.29, 3.7%)
LSU	2516,2568 (LSUF,LSUG)	1	gc=(209, 209.00, 100.0%)	gc=(209, 208.00, 99.5%)<br>au=(1, 0.00, 0.5%)	gc=(209, 200.37, 95.9%)<br>au=(9, 0.37, 4.1%)
LSU	2517,2567 (LSUF,LSUG)	1	cg=(209, 209.00, 100.0%)	cg=(210, 210.00, 100.0%)	cg=(210, 202.29, 96.3%)<br>ua=(8, 0.29, 3.7%)
LSU	2524,2539 (LSUG)	2	gc=(208, 208.00, 99.5%)<br>gu=(1, 1.00, 0.5%)	gc=(209, 209.00, 99.5%)<br>gu=(1, 1.00, 0.5%)	gc=(209, 201.33, 95.9%)<br>au=(8, 0.33, 3.7%)<br>gu=(1, 8.67, 0.5%)
LSU	2527,2536 (LSUG)	1	cg=(195, 181.94, 93.3%)<br>ua=(14, 0.94, 6.7%)	cg=(196, 182.93, 93.3%)<br>ua=(14, 0.93, 6.7%)	cg=(204, 190.90, 93.6%)<br>ua=(14, 0.90, 6.4%)
LSU	2547,2561 (LSUG)	1	au=(207, 205.02, 99.0%)<br>ua=(2, 0.02, 1.0%)	au=(208, 206.02, 99.0%)<br>ua=(2, 0.02, 1.0%)	au=(216, 214.02, 99.1%)<br>ua=(2, 0.02, 0.9%)
LSU	2594,2599 (LSUG)	1	cg=(209, 209.00, 100.0%)	cg=(210, 210.00, 100.0%)	cg=(213, 208.11, 97.7%)<br>ua=(5, 0.11, 2.3%)

rRNA[a]	Pair[b] (Fragments[c])	Category[d]	Hemosporidian[e]	+ Coccidian[f]	+ Piroplasm[g]
LSU	2742,2762 (RNA6)	2	ug=(195, 195.00, 93.3%)<br>cg=(14, 14.00, 6.7%)	ug=(195, 195.00, 93.3%)<br>cg=(14, 14.00, 6.7%)	ug=(195, 187.81, 89.9%)<br>cg=(14, 13.48, 6.5%)<br>au=(8, 0.29, 3.7%)
LSU	2743,2761 (RNA6)	2	au=(206, 206.00, 98.6%)<br>uu=(3, 3.00, 1.4%)	au=(206, 206.00, 98.6%)<br>uu=(3, 3.00, 1.4%)	au=(206, 198.41, 94.9%)<br>ug=(8, 0.41, 3.7%)<br>uu=(3, 10.59, 1.4%)
LSU	2744,2760 (RNA6)	2	gu=(209, 209.00, 100.0%)	gu=(209, 209.00, 100.0%)	gu=(209, 201.29, 96.3%)<br>aa=(8, 0.29, 3.7%)
SSU	27,556 (RNA14,SSUA)	2	gc=(208, 208.00, 99.5%)<br>ac=(1, 1.00, 0.5%)	gc=(208, 208.00, 99.5%)<br>ac=(1, 1.00, 0.5%)	gc=(208, 200.33, 95.9%)<br>aa=(8, 0.33, 3.7%)<br>ac=(1, 8.67, 0.5%)
SSU	28,555 (RNA14,SSUA)	1	gc=(209, 209.00, 100.0%)	gc=(209, 209.00, 100.0%)	gc=(209, 201.29, 96.3%)<br>ua=(8, 0.29, 3.7%)
SSU	30,553 (RNA14,SSUA)	2	ua=(209, 209.00, 100.0%)	ua=(209, 209.00, 100.0%)	ua=(209, 202.26, 96.3%)<br>cg=(7, 0.26, 3.2%)<br>ug=(1, 7.74, 0.5%)
SSU	32,552 (RNA14,SSUA)	1	au=(209, 209.00, 100.0%)	au=(209, 209.00, 100.0%)	au=(209, 201.29, 96.3%)<br>ua=(8, 0.29, 3.7%)
SSU	39,403 (RNA14)	2	cg=(205, 205.00, 98.1%)<br>gg=(3, 3.00, 1.4%)<br>ag=(1, 1.00, 0.5%)	cg=(205, 205.00, 98.1%)<br>gg=(3, 3.00, 1.4%)<br>ag=(1, 1.00, 0.5%)	cg=(205, 197.44, 94.5%)<br>gc=(5, 0.18, 2.3%)<br>au=(3, 0.06, 1.4%)<br>gg=(3, 7.71, 1.4%)<br>ag=(1, 3.85, 0.5%)
SSU	40,402 (RNA14)	2	cg=(206, 206.00, 98.6%)<br>ag=(3, 3.00, 1.4%)	cg=(206, 206.00, 98.6%)<br>ag=(3, 3.00, 1.4%)	cg=(206, 198.41, 94.9%)<br>ua=(8, 0.29, 3.7%)<br>ag=(3, 2.89, 1.4%)
SSU	41,401 (RNA14)	1	ua=(209, 209.00, 100.0%)	ua=(209, 209.00, 100.0%)	ua=(214, 211.04, 98.6%)<br>au=(3, 0.04, 1.4%)
SSU	293,304 (RNA17)	2	gc=(209, 209.00, 100.0%)	gc=(209, 209.00, 99.5%)<br>ga=(1, 1.00, 0.5%)	gc=(209, 201.33, 95.9%)<br>au=(8, 0.29, 3.7%)<br>ga=(1, 0.96, 0.5%)

rRNA[a]	Pair[b] (Fragments[c])	Category[d]	Hemosporidian[e]	+ Coccidian[f]	+ Piroplasm[g]
SSU	504,541 (SSUA)	2	ug=(110, 110.00, 52.6%)<br>cg=(98, 98.00, 46.9%)<br><br>gg=(1, 1.00, 0.5%)	ug=(111, 111.00, 52.9%)<br>cg=(98, 98.00, 46.7%)<br><br>gg=(1, 1.00, 0.5%)	ug=(111, 106.93, 50.9%)<br>cg=(98, 94.40, 45.0%)<br>au=(8, 0.29, 3.7%)<br>gg=(1, 0.96, 0.5%)
SSU	505,526 (SSUA)	2	ga=(207, 207.00, 99.0%)<br><br>aa=(2, 2.00, 1.0%)	ga=(208, 208.00, 99.0%)<br><br>aa=(2, 2.00, 1.0%)	ga=(208, 200.37, 95.4%)<br>ac=(8, 0.37, 3.7%)<br>aa=(2, 9.63, 0.9%)
SSU	507,524 (SSUA)	2	ua=(209, 209.00, 100.0%)	ua=(209, 208.00, 99.5%)<br>ag=(1, 0.00, 0.5%)	ua=(209, 200.37, 95.9%)<br>ag=(9, 0.37, 4.1%)
SSU	511,540 (SSUA)	2	ug=(209, 209.00, 100.0%)	ug=(209, 208.00, 99.5%)<br><br>cu=(1, 0.00, 0.5%)	ug=(210, 209.04, 96.3%)<br>ua=(7, 6.97, 3.2%)<br>cu=(1, 0.00, 0.5%)
SSU	513,538 (SSUA)	2	cu=(209, 209.00, 100.0%)	cu=(209, 208.00, 99.5%)<br><br>aa=(1, 0.00, 0.5%)	cu=(209, 208.04, 95.9%)<br>cg=(8, 7.96, 3.7%)<br>aa=(1, 0.00, 0.5%)
SSU	585,756 (SSUA,RNA8)	2	ug=(206, 206.00, 99.0%)<br><br>ua=(2, 2.00, 1.0%)	ug=(207, 207.00, 99.0%)<br><br>ua=(2, 2.00, 1.0%)	ug=(207, 199.37, 95.4%)<br>gu=(8, 0.29, 3.7%)<br>ua=(2, 1.93, 0.9%)
SSU	772,807 (RNA8)	2	ug=(207, 207.00, 99.5%)<br><br><br><br>ua=(1, 1.00, 0.5%)	ug=(207, 207.00, 99.0%)<br><br><br>ag=(1, 1.00, 0.5%)<br>ua=(1, 1.00, 0.5%)	ug=(207, 199.37, 95.4%)<br>gc=(5, 0.18, 2.3%)<br>gu=(3, 0.11, 1.4%)<br>ag=(1, 0.96, 0.5%)<br>ua=(1, 0.96, 0.5%)
SSU	774,805 (RNA8)	2	ac=(208, 208.00, 100.0%)	ac=(208, 208.00, 99.5%)<br><br>uc=(1, 1.00, 0.5%)	ac=(208, 200.33, 95.9%)<br>uu=(8, 0.33, 3.7%)<br>uc=(1, 8.67, 0.5%)
SSU	778,804 (RNA8)	2	gc=(206, 206.00, 99.0%)<br><br>gu=(2, 2.00, 1.0%)	gc=(207, 207.00, 99.0%)<br><br>gu=(2, 2.00, 1.0%)	gc=(207, 199.37, 95.4%)<br>ua=(8, 0.29, 3.7%)<br>gu=(2, 1.93, 0.9%)
SSU	784,798 (RNA8)	2	ug=(201, 201.00, 96.6%)<br><br>ua=(7, 7.00, 3.4%)	ug=(201, 201.00, 96.2%)<br><br>ua=(8, 8.00, 3.8%)	ug=(201, 193.59, 92.6%)<br>au=(8, 0.29, 3.7%)<br>ua=(8, 7.71, 3.7%)

rRNA[a]	Pair[b] (Fragments[c])	Category[d]	Hemosporidian[e]	+ Coccidian[f]	+ Piroplasm[g]
SSU	885,912 (SSUB)	2	gc=(209, 209.00, 100.0%)	gc=(209, 209.00, 99.5%)<br>gu=(1, 1.00, 0.5%)	gc=(209, 201.33, 95.9%)<br>ua=(8, 0.29, 3.7%)<br>gu=(1, 0.96, 0.5%)
SSU	894,905 (SSUB)	2	gu=(208, 208.00, 99.5%)<br>gc=(1, 1.00, 0.5%)	gu=(209, 209.00, 99.5%)<br>gc=(1, 1.00, 0.5%)	gu=(209, 201.33, 95.9%)<br>ua=(8, 0.29, 3.7%)<br>gc=(1, 0.96, 0.5%)
SSU	895,904 (SSUB)	2	ug=(207, 207.00, 99.0%)<br>ua=(2, 2.00, 1.0%)	ug=(208, 208.00, 99.0%)<br>ua=(2, 2.00, 1.0%)	ug=(208, 204.18, 95.4%)<br>ua=(5, 4.91, 2.3%)<br>au=(4, 0.09, 1.8%)<br>uu=(1, 4.91, 0.5%)
SSU	930,1387 (SSUB,SSUD)	2	cg=(208, 208.00, 99.5%)<br>ug=(1, 1.00, 0.5%)	cg=(208, 208.00, 99.0%)<br>ug=(2, 2.00, 1.0%)	cg=(208, 200.37, 95.4%)<br>ua=(8, 0.37, 3.7%)<br>ug=(2, 9.63, 0.9%)
SSU	931,1386 (SSUB,SSUD)	1	au=(209, 209.00, 100.0%)	au=(210, 210.00, 100.0%)	au=(213, 208.11, 97.7%)<br>gc=(5, 0.11, 2.3%)
SSU	933,1384 (SSUB,SSUD)	1	ag=(209, 209.00, 100.0%)	ag=(209, 209.00, 100.0%)	ag=(209, 201.29, 96.3%)<br>ga=(8, 0.29, 3.7%)
SSU	939,1344 (SSUB,SSUD)	2	gu=(208, 208.00, 99.5%)<br>au=(1, 1.00, 0.5%)	gu=(208, 208.00, 99.0%)<br>au=(2, 2.00, 1.0%)	gu=(208, 200.37, 95.4%)<br>ua=(8, 0.29, 3.7%)<br>au=(2, 1.93, 0.9%)
SSU	941,1342 (SSUB,SSUD)	1	gc=(209, 209.00, 100.0%)	gc=(210, 210.00, 100.0%)	gc=(210, 202.29, 96.3%)<br>au=(8, 0.29, 3.7%)
SSU	962,973 (SSUB)	1	ua=(106, 53.76, 50.7%)<br>cg=(103, 50.76, 49.3%)	ua=(106, 53.50, 50.5%)<br>cg=(104, 51.50, 49.5%)	ua=(114, 59.61, 52.3%)<br>cg=(104, 49.61, 47.7%)
SSU	986,1219 (SSUB,RNA9)	1	au=(209, 209.00, 100.0%)	au=(209, 209.00, 100.0%)	au=(209, 201.29, 96.3%)<br>ga=(8, 0.29, 3.7%)
SSU	989,1216 (SSUB,RNA9)	2	cg=(207, 205.02, 99.0%)<br>au=(1, 0.00, 0.5%)<br>ua=(1, 0.00, 0.5%)	cg=(207, 205.02, 99.0%)<br>au=(1, 0.00, 0.5%)<br>ua=(1, 0.00, 0.5%)	cg=(207, 197.46, 95.4%)<br>aa=(8, 0.37, 3.7%)<br>au=(1, 0.04, 0.5%)<br>ua=(1, 0.04, 0.5%)

rRNA[a]	Pair[b] (Fragments[c])	Category[d]	Hemosporidian[e]	+ Coccidian[f]	+ Piroplasm[g]
SSU	1411,1489 (SSUE,SSUF)	1	cg=(209, 209.00, 100.0%)	cg=(210, 210.00, 100.0%)	cg=(210, 202.29, 96.3%)<br>ua=(8, 0.29, 3.7%)
SSU	1514,1521 (SSUF)	1	au=(209, 209.00, 100.0%)	au=(209, 208.00, 99.5%)<br>gc=(1, 0.00, 0.5%)	au=(217, 216.00, 99.5%)<br>gc=(1, 0.00, 0.5%)

[a] LSU, large subunit ribosomal RNA; SSU, small subunit ribosomal RNA.

[b] Base pairs with significant potential covariation are shown. Pairs are named using the *E. coli* rRNA reference structure model numbering.

[c] Fragment containing each position (not repeated if both positions are in the same fragment).

[d] Category 1, strong covariation signal without exceptions (red in Figures S9-S11); 2, covariation signal with exceptions (blue in Figures S9-S11).

[e] 200 sequences from *Plasmodium* and nine related hemosporidians, for a total of 209 sequences.

[f] All sequences from the hemosporidian alignment, plus one coccidian sequence, for a total of 210 sequences.

[g] All sequences from the hemosporidian and coccidian alignment, plus eight piroplasm sequences, for a total of 218 sequences.

[h] Background shading: red, invariant; green, coccidian does not add information to hemosporidian; blue, piroplasm does not add information to coccidian.

[i] Covariation data: <base pair type>=(<number of occurrences observed in dataset>, <number of occurrences expected based upon random distribution of observed nucleotides at the two positions>, <percentage of sequences containing the base pair type>).

[j] Text color: green, coccidian has that combination at that base pair (may overlap with hemosporidian); blue, at least one of the eight piroplasm sequences has that combination at that base pair (may overlap with hemosporidian and/or coccidian).
