# Supplementary material for: The Fragmented Mitochondrial Ribosomal RNAs of Plasmodium falciparum
Source: PLoS One. 2012 Jun 22;7(6):e38320. doi: 10.1371/journal.pone.0038320 (PMC3382252; doi:10.1371/journal.pone.0038320)
Supplement: Table S9 — Correspondence of P. falciparum and C. elegans mt rRNAs. (PDF) [file pone.0038320.s025.pdf]

Table S9. Correspondence of *P. falciparum* and *C. elegans* mt rRNAs.

SSU

**helix**[a]	***P. falciparum***[b]	***C. elegans***[b]
9	***missing***	present
17	***missing***	present
27	RNA14:SSUA	present
39	RNA14	present
122	RNA12	***missing***
240	RNA12	***missing***
289	RNA17	***missing***
500	SSUA	present
505	SSUA	present
511	SSUA	present
567	SSUA:SSUB	present
575	SSUA:SSUB	present
577	SSUA:RNA8	present
655	***missing***	present; short
673	***missing***	present
722	***missing***	present
769	RNA8	present
821	RNA8:SSUB	***missing***
885	SSUB	present
921	SSUB:SSUE	present
939	SSUB:SSUD	present
944	SSUB:RNA9	present
960	SSUB	present
984	SSUB:RNA9	present
1047	RNA19:RNA9; ***short***	present
1068	missing	present
1074	missing	present
1303	SSUD:RNA5	present
1350	SSUD	present
1399	SSUE:SSUF; short	present; short
1506	SSUF	present

5' LSU

**helix**[a]	***P. falciparum***[b]	***C. elegans***[b]
563	***missing***	present
579	LSUA:RNA1	present
589	LSUA; short	present; short
671	LSUA	present
687	LSUA	present
700	LSUA: ***short***	***missing***
736	LSUA	present
777	LSUA	present
812	LSUA	present
822	LSUA	missing
946	RNA2	***missing***
976	RNA2	***missing***
991	RNA2:RNA11	***unstructured***
1030	missing	present, short
1057	LSUB	present
1082	LSUB:LSUC	***missing***
1087	LSUC	present
1164	RNA11	***missing***
1196	RNA11:RNA1, short	present; short
1262	RNA1:LSUE	present
1276	RNA1	missing
1295	RNA1:RNA3	missing

[a] Helix numbers correspond to the first nt in the corresponding helices in *E. coli* rRNA secondary structures

3'LSU

**helix**[a]	***P. falciparum***[b]	***C. elegans***[b]
1648	RNA3:LSUE	missing
1764	LSUD:LSUE	present
1775	LSUD	present
1782	LSUD	present
1792	LSUD	present; short
1830	LSUD:LSUE	present
1835	LSUD:LSUE; short	present; short
1906	LSUE	present
1925	LSUE	present
1935	LSUE	present
2023	LSUE	present
2043	LSUE:LSUG	present
2064	LSUE:LSUF	present
2077	LSUE:RNA13	present
2246	RNA13	present
2455	LSUF	present
2507	LSUF:LSUG	present
2520	LSUF:LSUG	present
2547	LSUG	present
2588	LSUG	present
2646	RNA10	present
2675	RNA10/RNA18:RNA6	present; ***short***
2735	RNA6	present

[b] Significant differences between *P. falciparum* and *C. elegans* rRNA structure (absence or altered size) are in bold italic font
